# Development of SNP genotyping assays for heading date in rice

**DOI:** 10.1270/jsbbs.23093

**Published:** 2024-06-25

**Authors:** Noriyuki Kitazawa, Ayahiko Shomura, Tatsumi Mizubayashi, Tsuyu Ando, Nagao Hayashi, Shiori Yabe, Kazuki Matsubara, Kaworu Ebana, Utako Yamanouchi, Shuichi Fukuoka

**Affiliations:** 1 Institute of Crop Science, National Agriculture and Food Research Organization (NARO), 2-1-2 Kannondai, Tsukuba, Ibaraki 305-8518, Japan; 2 Institute of Agrobiological Sciences, NARO, 2-1-2 Kannondai, Tsukuba, Ibaraki 305-8518, Japan; 3 Genetic Resources Center, NARO, 2-1-2 Kannondai, Tsukuba, Ibaraki 305-8602, Japan

**Keywords:** functional nucleotide polymorphism, genotyping assay, heading date, Japanese cultivar, rice, single nucleotide polymorphism

## Abstract

Heading date (HD) is a crucial agronomic trait, controlled by multiple loci, that conditions a range of geographical and seasonal adaptations in rice (*Oryza sativa* L.). Therefore, information on the HD genotypes of cross parents is essential in marker-assisted breeding programs. Here, we used the Fluidigm 96-plex SNP genotyping platform to develop genotyping assays to determine alleles at 41 HD loci (29 previously characterized genes and 12 quantitative trait loci [QTLs], including a newly detected QTL). The genotyping assays discriminated a total of 144 alleles (defined on the basis of the literature and publicly available databases) and QTLs. Genotyping of 377 cultivars revealed 3.5 alleles per locus on average, a higher diversity of *Hd1*, *Ghd7*, *PRR37*, and *DTH8* than that of the other loci, and the predominance of the reference (‘Nipponbare’) genotype at 30 of the 41 loci. HD prediction models using the data from 200 cultivars showed good correlation (*r* > 0.69, *P* < 0.001) when tested with 22 cultivars not included in the prediction models. Thus, the developed assays provide genotype information on HD and will enable cost-effective breeding.

## Introduction

Heading date (HD) is a major determinant of yield and grain quality of harvested rice (*Oryza sativa* L.) and is determined by genetic and environmental factors, in particular by daylength (Endo-Higashi and Izawa 2011, Itoh and Izawa 2013). In rice breeding programs, the use of a series of cultivars with diverse HDs could enable the expansion of adaptation areas and the diversification of cropping seasons (Fujino *et al.* 2022, Izawa 2007, Takeuchi *et al.* 2006). Therefore, researchers have focused on elucidating the genetic basis of this trait, and more than 70 HD-related genes have been isolated and functionally characterized (Matsubara and Yano 2018, Zhou *et al.* 2021).

Despite progress in our understanding of genes for HD, most genetic studies assess the effect of alleles on a biparental basis. The genotype data of breeding stocks obtained from these studies are limited to alleles with major effects and are scattered in the literature. Therefore, the distinct effects of multiple alleles at certain loci such as *Hd1*, *PRR37*, and *DTH8* have not been summarized, which makes it difficult to predict variation of HD in a breeding population. Although next-generation sequencing has produced vast genomic information, including on HD-related alleles, it remains costly. Restriction-site–associated DNA (RAD) sequencing is cheaper, but it provides SNPs only around the targeted restriction sites, so mutations in target genes are not captured.

We have developed a platform for Fluidigm 96-plex SNP genotyping assays to detect 24 blast resistance alleles at 10 loci in rice (Kitazawa *et al.* 2019). The platform genotypes alleles on the basis of SNPs, distinguishing multiple polymorphisms at the same locus (Kitazawa *et al.* 2019, Kurokawa *et al.* 2016, Qian *et al.* 2017).

The main goal of this study was to establish efficient genotyping assays for HD loci that contribute to HD variation in breeding stocks. In addition to a previously uncharacterized QTL for HD, we developed a set of genotyping assays based on information from previously reported HD loci.

## Materials and Methods

### Plant materials and growth conditions

To design and evaluate the SNP-genotyping assays for HD genes and QTLs, we used the 377 cultivars listed in Supplemental Table 1. The primary validation of the assays used 182 major Japanese cultivars, and then the assays were applied to all 377 cultivars.

To find a previously uncharacterized QTL for HD, we used HD and genotype data of a set of recombinant inbred lines (RILs) derived from a cross between temperate *japonica* ‘Kanto 209’ (K209) and ‘Koshihikari Aichi SBL’ (KoASBL), 138 F_4_ or F_5_ lines (2011_RILs), and 153 F_5_ or F_6_ lines (2012_RILs) from a previous study (Inoue *et al.* 2017). The HD of ‘KoASBL’ is the same as that of ‘Koshihikari’ (Sugiura *et al.* 2004), and that of ‘K209’, later registered as ‘Satojiman’, is about 10 days later than ‘Koshihikari’ (Sato *et al.* 2013). The RILs were transplanted in early June and grown in an experimental field at the Aichi Agricultural Research Center, Mountainous Region Agricultural Research Institute, Toyota (137°51ʹE, 35°21ʹN).

As training data for constructing prediction models based on the developed genotyping assays, we recorded the HDs of 200 out of the 377 cultivars that had marker genotypes (Supplemental Table 2). The cultivars were grown over 11 years (2010–2020) with three sowing dates (mid-April, mid-May, and mid-June) in an experimental paddy field at the Institute of Crop Science, NARO, Tsukuba (140°01ʹE, 35°59ʹN).

To assess the models, we used 22 recently released cultivars and their parents to obtain test data. They were grown over three years (2018–2022; sown in mid-April) in the same field as the 200 cultivars. They were included in the 377 cultivars, but not in the 200 training cultivars (Supplemental Table 3). This partitioning of the training and test data was designed to mimic the application of our models in an actual breeding program, i.e., prediction for newly released cultivars based on information obtained from the existing cultivars.

### Detection and extraction of DNA polymorphisms at HD loci

To detect candidate polymorphims for the SNP genotyping assay for HD, we used three criteria: (1) Functional nucleotide polymorphisms (FNPs) identified using map-based cloning of the causal genes of natural variations of HD were included. FNPs for the QTLs with large effects, such as *Hd6*, *Hd1*, *Ghd7*, *PRR37*, and *DTH8*, and those with small effects, such as *DTH2*, *Hd16*, *Hd17*, and *Hd18*, were collected from previous reports (Supplemental Table 4). (2) Putative FNPs in HD genes, mainly from *japonica* cultivars, were included, obtained from the publicly available SNP databases TASUKE+ (agrigenome.dna.affrc.go.jp/tasuke/ricegenomes/, Kumagai *et al.* 2019), TASUKE+ with genome-wide association studies (GWAS) (https://tasuke-plus.dna.affrc.go.jp/, Yano *et al.* 2016), TASUKE+ for the NARO Genebank Rice Core Collection (World Rice Core collection [WRC]+ Japanese Rice Core collection [JRC]) (https://ricegenome-corecollection.dna.affrc.go.jp/, Tanaka *et al.* 2020, 2021), RiceVarMap v. 2.0 (http://ricevarmap.ncpgr.cn/, Zhao *et al.* 2021), and SNP-Seek (https://snp-seek.irri.org/index.zul, Alexandrov *et al.* 2015). (3) DNA polymorphisms around HD genes or QTLs found by QTL mapping or GWAS in Japanese cultivars, and around QTLs found in the present study were included. We used whole-genome re-sequencing data of cultivars of various geographical origins (mainly from the NCBI SRA database, https://www.ncbi.nlm.nih.gov/sra/, but also our unpublished data) mapped to the ‘Nipponbare’ IRGSP-1.0 reference sequence (Kawahara *et al.* 2013) in CLC Genomics Workbench v. 8.0 software (Qiagen, Hilden, Germany) and DNA polymorphisms reported previously (Yonemaru *et al.* 2012). All extracted polymorphisms were listed according to their physical positions on the ‘Nipponbare’ IRGSP-1.0 reference sequence.

Before designing the SNP genotyping assay, we assessed the list against three criteria to ensure the discriminating ability of the assay: (1) The absence of another polymorphism around the target mutation was inspected by in CLUSTALW v. 2.1 multiple sequence alignment software (http://www.clustal.org/clustal2/). (2) The absence of multi-copy sequences around the target mutation in the ‘Nipponbare’ IRGSP-1.0 reference sequence was inspected by using the BLAST+ 2.8.1 basic local alignment search tool (https://blast.ncbi.nlm.nih.gov/Blast.cgi). (3) From the viewpoint of discriminability of alleles at the HD loci, we prioritized previously reported FNPs, putative FNPs, and non-synonymous substitutions, in this order.

### Development and assessment of Fluidigm SNP genotyping assays

SNP and insertion/deletion (indel) polymorphisms were used for SNP genotyping assays, which included at least 60-bp sequences on both sides of the target polymorphism. These sequences were obtained from the ‘Nipponbare’ IRGSP-1.0 reference genome (Kawahara *et al.* 2013) and were synthesized by Fluidigm Inc (https://www.standardbio.com/). Signal intensity, allelic signal balance, and the discriminating ability of SNP-based alleles in each assay were preliminarily tested on 182 cultivars, and a set of 96-plex SNP genotyping assays was determined. To test the operation of this set, we determined genotypes of all 377 cultivars (Supplemental Table 1), including those of Japanese modern high-yielding cultivars (mainly for animal feed use) and Japanese landraces from JRC (Ebana *et al.* 2008).

### Genotyping

**Fig. 1.**
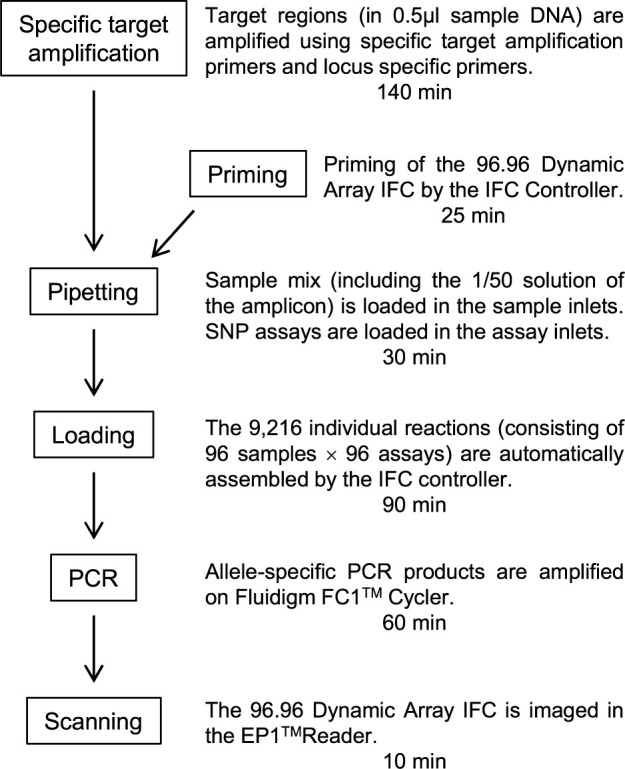
A brief genotyping workflow using the assays.

Total DNA (probably <20 ng/μl) was extracted from small pieces of fresh leaves as described by Kitazawa *et al.* (2019). Genotyping of these DNA samples with SNP genotyping assays was performed on the 96.96 Dynamic Array IFC (96.96 IFC) chip according to the “SNPtype 96×96 v1” protocol supplied by Fluidigm Inc., except that the standard number of additional cycles after touchdown PCR was reduced from 34 to 30. A brief description of the workflow was shown in Fig. 1. To determine the appropriate number of cycles for genotype discrimination, we performed two additional sets of six cycles each (i.e., 36 and 42 cycles in total) and monitored the behavior of the scatter plot based on signal intensity. To improve the call rate of genotyping at low quantity or quality of input DNA, we performed specific target amplification with 14 cycles before the allele-specific reaction. Scanned data obtained on an EP1 reader were analyzed with Fluidigm SNP Genotype Analysis software v. 4.5.1 and converted to scatter plot diagrams and allele-type data. Then, alleles at each locus were classified according to their type, and numbers were assigned to them. To confirm that SNP genotyping assays targeted indel polymorphisms, we used six indel markers (Supplemental Table 5) and confirmed respective genotypes by using standard PCR and agarose gel electrophoresis.

### QTL mapping of HD in RILs

Genotypes of 2011_RILs and 2012_RILs were obtained from a previous study (Inoue *et al.* 2017). For 2012_RILs, the genotypes of nine simple sequence repeat (SSR) markers on chromosome 5 (RM3529, RM4838, RM17896, RM17928, RM17935, RM17990, RM1089, RM18068, and RM4837) (International Rice Genome Sequencing Project and Sasaki 2005, McCouch *et al.* 2002) were also used. QTL analysis was based on the genotypes and days-to-heading (DTH) data (Supplemental Table 4) of all RILs by R/qtl v. 1.47.9 software (Broman *et al.* 2003). Genotype probabilities were calculated with the *calc.genoprob* function with a step size of 2 cM and the *Kosambi map* function. A total of 193 markers for 2011_RILs and 197 markers for 2012_RILs, including the 9 SSR markers listed above, were used. Composite interval mapping (CIM) was performed using the *cim* function with the Haley–Knott regression method, a window size of 10 cM, and three marker covariates. The location of putative major QTLs was estimated by using the genome-wide logarithm of odds (LOD) threshold at the 5% significance level calculated from 1000 permutation tests. QTLs with minor effects were detected by using a fixed LOD threshold of 3.0. The marker closest to the peak of the LOD curve was defined as the estimated QTL. The *makeqtl* and *fitqtl* functions were used to estimate the percentage of phenotypic variance explained (PVE) by each QTL. The interaction between QTLs and additional QTLs was explored by using multiple interval mapping (MIM) on the basis of the putative QTLs detected by CIM. Genotype data were calculated by using the *sim.geno* function with a simulation with 128 replicates. MIM was performed with the *stepwiseqtl* function and was based on the number of QTLs detected using CIM.

### Multiple regression analysis

To assess the effectiveness of the assays, we constructed prediction models by multiple linear regression analyses in R v. 4.3.0 software (R Core Team 2023). A model was constructed for each cropping season (starting in April, May, and June). Alleles were used as explanatory variables and DTH data (mean DTH across 11 years in each of the three cropping seasons) as response variables obtained from the 200 training cultivars. The explanatory variables were arranged as dummy variables, in which the allele of ‘Nipponbare’ was treated as a reference for each marker in the models. The analysis was performed as follows: (1) The *lm* function was used to fit linear models. (2) The *step* function was used to select variables by Akaike’s information criterion with the “both” option. (3) Variables with variance inflation factors ≥10 were excluded from the models to reduce collinearity by using the *vif* function in the DAAG package (v. 1.25.4, Maindonald and Braun 2022). (4) The number of variables was reduced on the basis of the *P*-values (<0.1) for regression coefficients.

The predictability of the model was evaluated by using 22 test cultivars on the basis of Pearson’s correlation between observed and predicted values. Prediction was performed by using model_April for each year’s data because the test cultivars were sown in mid-April.

## Results

### Extraction of HD loci and natural variations

From the exhaustive literature and database survey, we selected 107 HD loci that were identified mostly by physical or chemical mutagenesis (Supplemental Table 6) and excluded the loci without a natural functional variation in public SNP databases or our NGS data. Finally, 40 HD loci with 218 variants were targeted for assay design (Supplemental Table 6), including the *Hd3a* locus, whose 4939-bp insertion in the promoter region does not appear to alter HD (Supplemental Table 4). The average number of polymorphisms extracted per locus was 5.5 (range, 1–29), with 29 at the *Hd1* locus, followed by 18 at *Ghd7*, 16 at *DTH8*, and 12 at *PRR37* (Supplemental Table 6).

### HD QTLs for genotyping assay

To develop assays for the causal genes for HD that have not been cloned, we selected 7 QTLs from 27 publications on QTL mapping or GWAS (Table 1). To search for novel QTLs for HD, we performed QTL mapping for HD using RILs from a cross between ‘KoASBL’ and ‘K209’. Both CIM and MIM detected eight QTLs on five chromosomes (Supplemental Table 7, Supplemental Fig. 1A–1C); four of them were detected around the previously reported HD loci *OsGI*, LOC_Os01g62780, *OsMADS51*, *OsMADS15*, and *Hd18* (Supplemental Table 7). The rest—*qDTH6*, *qDTH7-2*, *qDTH8-2* and *qDTH9* on chromosomes 6, 7, 8, and 9, respectively—were identified in the regions where no QTLs for HD have been reported (Supplemental Table 7). Taking into account their reproducibility and additive effects, we selected only *qDTH7-2* for the genotyping assay design. In total, we selected 12 QTLs (QTL_01–12) for assay setting (Table 1).

### Validation and selection of representative genotyping assays

**Fig. 2.**
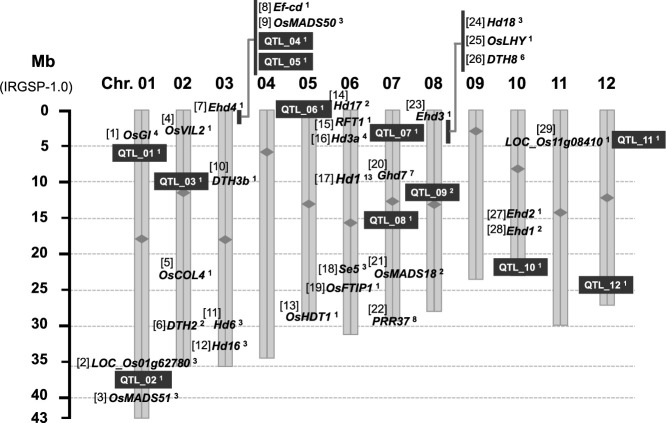
Chromosome locations of the 41 loci (29 genes and 12 QTLs) targeted in rice genotyping assays. Scale in Mb (‘Nipponbare’ IRGSP-1.0) is indicated on the left. The 29 genes are listed under “Gene name” in Table 2. The 12 QTLs are listed under “QTL number” in Table 1. The superscript at the end of each locus label indicates the number of assays used for the locus. Centromeres are indicated by a rhombus on each chromosome.

To confirm the discriminating ability of SNPs, we tested the genotyping assays using genomic DNA from 182 diverse cultivars. On the basis of signal intensity, the allelic signal balance in respective assays, and the discriminating ability of the SNP-based alleles, we selected a set of 96-plex SNP genotyping assays for the Fluidigm 96.96 IFC chip platform and designated them as “Heading date–related loci assays version 1 (HDA1)”. HDA1 covered 41 loci (29 genes and 12 QTLs) on 10 of the 12 rice chromosomes (except chromosomes 4 and 9; Fig. 2) and consisted of 83 assays for the genes (Table 2, Supplemental Fig. 2) and 13 assays for the QTLs (Table 1). HDA1 was expected to discriminate 144 alleles at 41 loci, including 29 genes and 12 QTLs. The design and specification for each assay are shown in Supplemental Table 8, and assay details for the three large indel polymorphisms are shown in Supplemental Fig. 3.

### Application of HDA1 to diverse rice cultivars

**Fig. 3.**
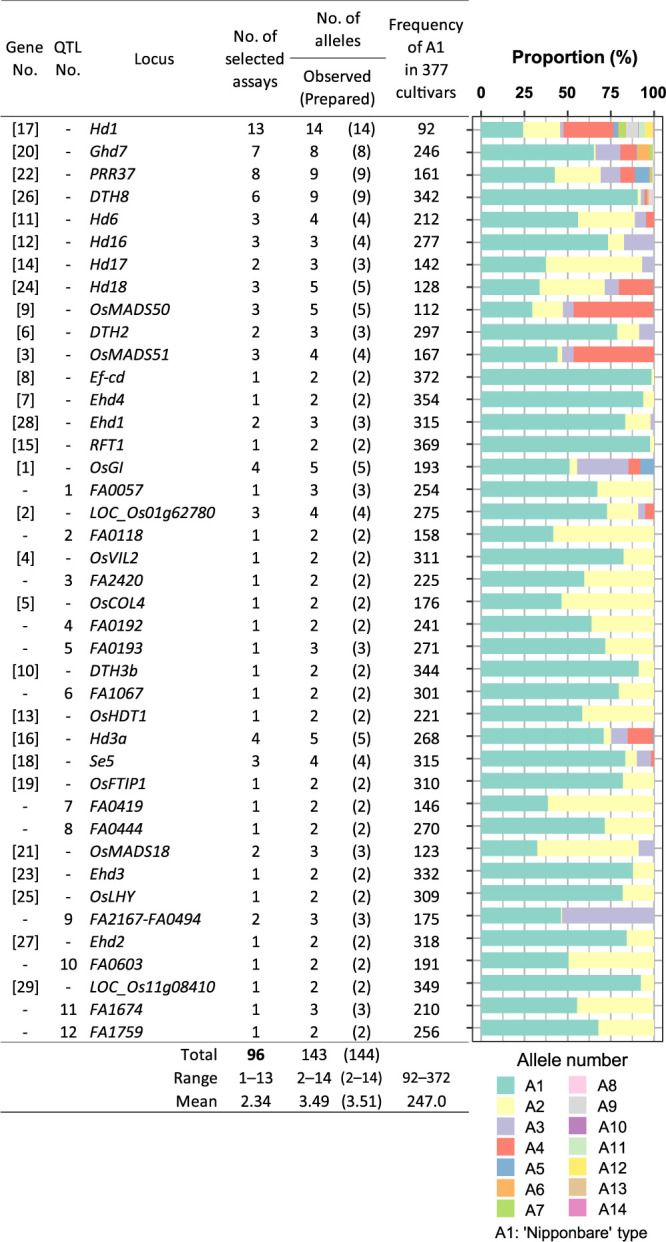
Distribution of alleles at 41 loci in 377 cultivars. The data were obtained in the developed genotyping assays.

To test the selected genotyping assays in diverse rice samples for the Japanese breeding programs, we determined genotypes of 377 cultivars with HDA1 (Supplemental Table 1, Supplemental Fig. 4). A total of 143 alleles could be observed, because one allele at the *Hd16* locus was not included in 377 cultivars (Fig. 3). The average number of alleles in these cultivars was 3.5 per locus and was greatest at *Hd1*, *Ghd7*, *PRR37*, and *DTH8* (Fig. 3). The reference (‘Nipponbare’) alleles were predominant at 30 of 41 loci (Fig. 3).

**Fig. 4.**
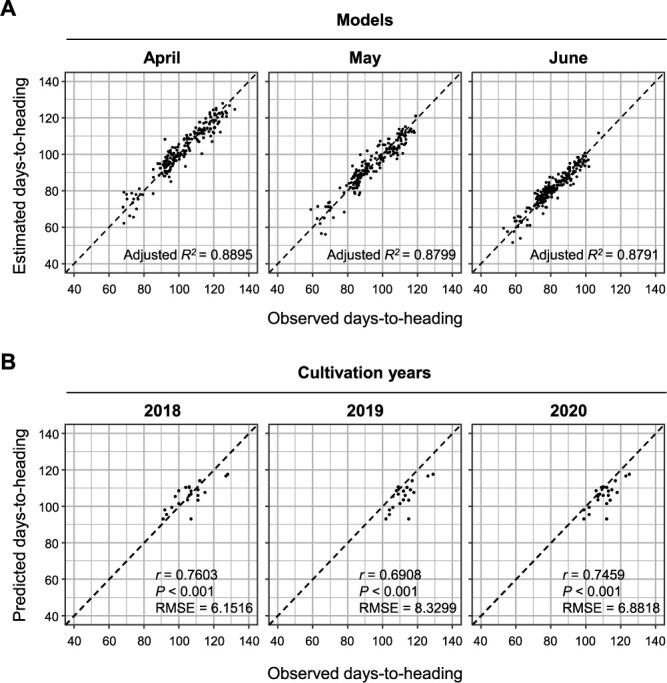
Construction of prediction models for days-to-heading (DTH) by multiple linear regression analyses using the assays. (A) Three prediction models constructed for each sowing time using 200 cultivars. Dashed line, 1:1 relationship. (B) Relationships between observed and predicted DTH. The model (Model_April) was applied to 22 cultivars. Dashed line, 1:1 relationship.

To grasp the utility and limitations of the assays, we constructed three prediction models by conventional multiple regression analysis using 200 cultivars with DTH data for every sowing time (Fig. 4A, Supplemental Table 2). A total of 138 alleles from these 200 cultivars were applied as explanatory variables (Supplemental Table 9). The resultant models included 21 HD loci, 10 of which had regression coefficients of >5 or <–5, despite the predominant contribution of *Hd1*, *Ghd7*, and *Se5* to DTH variation across the three sowing times (Supplemental Table 9). The adjusted *R*^2^ values were 0.8895 in model_April, 0.8799 in model_May, and 0.8791 in model_June. The predictability of model_April constructed with training data of cultivars sown in April was assessed for 22 test cultivars sown in mid-April. The predictability for DTH was statistically significant (Pearson’s correlation coefficient *r* = 0.7603 in 2018, *r* = 0.6908 in 2019, and *r* = 0.7459 in 2020) (Fig. 4B).

## Discussion

Here, we developed genotyping assays, based on a set of 96 SNPs (HDA1), to determine 144 alleles at 41 loci that included 29 genes and 12 QTLs. The genotype data for Japanese breeding stocks obtained in this study and our DNA-based marker system could be applied to comprehensive and rapid identification of HD genotypes at each experimental station.

The use of HDA1 makes it possible to rapidly determine HD genotypes, which would be beneficial for practical breeding programs. Genotyping of 96 samples can be done in about half a week from DNA extraction and costs about 170,000 JPY. If we could determine the genotype at each of the 41 HD loci in a single round of experiment, cross parents could be selected to minimize the number of HD loci that segregate in early generations after crossing. Since the genes to be selected in early generations are limited to a few other than the major genes such as disease resistance (Yamamoto *et al.* 1996), the introduction of HDA1 to the breeding procedures in the initial populations would radically reduce the number of individuals to be tested in the field.

Although rice heading time has been reviewed (Hori *et al.* 2016, Zhou *et al.* 2021), neither of these reviews considers the role of alleles, and breeders have not been able to refer to many cultivars. Accumulation of large-scale resequencing data generated by next-generation sequencing technologies and the availability of genome browsers such as TASUKE+ (https://tasuke.dna.affrc.go.jp/) offer a comprehensive view of DNA sequence variation throughout the genome, but the workflow required to summarize information and make a decision is unfriendly to breeders, and the cultivars listed in genome browsers are not sufficient for breeding. This study is particularly valuable because the use of HDA1 is currently the most sophisticated procedure to determine HD genotypes, including information on QTLs for which no gene has been isolated; however, HDA1 does not take into account all HD variations, so it needs to be updated on the basis of new findings.

We found new alleles, such as the *Hd1* allele No. A14 carried by ‘Jarjan’ and the *DTH8* allele No. A07 carried by ‘Muha’ (Supplemental Table 1), in the WRC but not in the Japanese cultivars. To clarify the value of these alleles in breeding programs in Japan, it will be necessary to characterize their effects on HD and on other agronomic traits such as plant height and grain yield (Liu *et al.* 2020, Xue *et al.* 2008). The HD of chromosome segment substitution lines carrying the *DTH8* allele No. A07 from ‘Muha’ (SL2628, SL2629, and SL2630) was later than that of the recurrent parent and close to that of line SL3029, carrying the ‘Basilanon’ allele No. A03, so these alleles might be functionally similar to each other (Nagata *et al.* 2023). The accumulation of knowledge about the relationship between traits is important in marker-assisted selection for HD. Because epistasis between alleles at different loci often affects rice HD (sometimes leading to extremely late heading; Matsubara *et al.* 2019, Uwatoko *et al.* 2008), genotype selection should be based on allele combinations. When foreign cultivars are used as cross parents, the genotype information obtained by HDA1 should be useful for removing late-heading individuals, although certain limitations may exist.

Our assays’ predictability of HD should be of interest to breeders to develop cultivars with a certain flowering time to meet farmers’ demands. While we used a conventional linear model to evaluate the assays, our results obtained with 22 cultivars that were not included in the prediction models were statistically significant, with fairly high correlation coefficients (Fig. 4B). In practical breeding, high predictability is desired, whereas the HD loci that we used may be insufficient for precise DTH prediction, because root mean squared errors ranged from 6.15 to 8.33. Nevertheless, the advantage of HDA1 is that it can be easily improved by replacing and adding assays, and the prediction model is also adjustable.

Thus, our assays, which discriminate 144 alleles at 41 loci, reveal genotypes involved in HD variation in rice cultivars. The assays will facilitate selection of cross parents and DNA markers, which will reduce the number of individuals in breeding. However, the HD of even the same genotypes can be affected by local cultural practices and climate fluctuations. Because the ideal HD genotypes depend on genetic backgrounds and breeding objectives, we will have to persist in our efforts to update trait data and genotype information on HD. The system of SNP genotyping assays will be applicable not only to HD and rice blast disease, but also to other quantitative traits such as yield and abiotic stress for which related QTLs are increasingly being identified.

## Author Contribution Statement

NK, UY and SF designed the study. NK, AS, TM, TA and SY developed the genotyping assays and performed the genotyping and statistical analyses. NK, SY, KM and SF wrote the manuscript. NH and KE provided the plant materials and HD data. KM and SF have equal and separate responsibilities for this manuscript. They are therefore the corresponding authors. All authors read and approved the final manuscript.

## Supplementary Material

Supplemental Figures

Supplemental Tables

## Figures and Tables

**Table 1.** Twelve heading date–related QTLs selected for assay development in this study

QTL number	Assay	Description of QTLs										
		QTL name	QTL nearest marker or marker interval	Marker type	Chr.*^a^*	Position_Start*^b^* (bp)	Position_End*^b^* (bp)	LOD*^c^*	PVE*^d^* (%)	Known gene	Mapping population	Reference*^e^*
QTL_01	FA0057	*qDTH1-1*	aa01000889–aa01005142	SNP	1	1,842,582	4,838,836	3.05/4.02	3.13/1.75	*OsGI*	Kanto 209/Koshihikari Aichi SBL RILs	This study
		*qDH1*	RM8093	SSR	1	8,773,474	8,773,629	4.2	8.9	*RDD1*	Sakihikari/Nipponbare 188 RILs	Kobayashi and Tomita (2008)
QTL_02	FA0118	*qDTH1*	RM265	SSR	1	35,197,616	35,197,724	2.53	8.1	*LOC_Os01g62780*, *OsMADS51*	Moritawase/Koshihikari 92 RILs	Wada *et al.* (2008)
		*qDTH1-2*	aa01010816	SNP	1	37,507,621	37,507,621	4.75	3.86		Kanto 209/Koshihikari Aichi SBL RILs	This study
QTL_03	FA2420	—	aa02000715	SNP	2	6,104,069	6,104,069	4.80	10.45		Tachisugata/Hokuriku193 191 RILs	Matsubara *et al.* (2016)
		—	RM12921–RM13165	SSR	2	9,502,890	15,563,437	6.2	56.9	*OsUbDKgamma4*	Koshihikari/Khau Mac Kho BC_4_F_2_ and BC_4_F_3_	Hori *et al.* (2015)
		—	B17 (P0237)	SNP	2	10,555,862	10,555,862	6.6	7.6	*OsUbDKgamma4*	Tachisugata/Hokuriku193 402 F_2_	Matsubara *et al.* (2015)
QTL_04	FA0192	—	GBR3003	SSR	3	1,690,591	1,690,866	0.0000^†^	—	*OsMADS50*	Hoshinoyume/Iburiwase F_2_	Fujino and Sekiguchi (2008)
QTL_05	FA0193	—	aa03000455	SNP	3	2,196,417	2,196,417	8.59/5.48	29.6/23.2		Hinohikari/Nikomaru 84 RILs	Kobayashi *et al.* (2015)
		—	RM4352	SSR	3	4,315,168	4,315,266	5.6	76.0		Koshihikari/IR64 BC_4_F_2_ (Koshihikari genetic background)	Nonoue *et al.* (2019)
QTL_06	FA1067	*qHD5b*	RM1248	SSR	5	93,969	94,104	0.0002526^†^	—	*qHD5*	Hokkaido Rice Core Panel (HRCP): 63 landraces and breeding lines in Hokkaido	Fujino *et al.* (2015)
		—	RM1248–RM18055	SSR	5	93,969	5,878,157	4.2	25		Koshihikari/Bei Khe BC_4_F_2_	Hori *et al.* (2015)
QTL_07	FA0419	*qDTH7*	aa07000566– aa07001067	SNP	7	2,072,143	3,654,626	4.20	2.4	*OsMADS15*	Koshihikari/Yamadanishiki 94 F_2_	Okada *et al.* (2017)
		*qDTH7-1*	aa07000615–aa07001067	SNP	7	2,651,062	3,654,626	3.07/3.12	2.79/1.60	*OsMADS15*	Kanto 209/Koshihikari Aichi SBL RILs	This study
		*qDTH7-1*	(by GWAS)	—	7	3,857,839	5,220,414	3.50E–06^†^	—	*OsMADS15*	135 cultivars of *temperate japonica* rice in Japan	Chigira *et al.* (2020)
QTL_08	FA0444	*qDTH7-2*	ac07000440–aa07001934	SNP	7	16,970,876	19,241,445	5.43	2.26		Kanto 209/Koshihikari Aichi SBL RILs	This study
QTL_09	FA2167, FA0494	*qDH8*	RM4085	SSR	8	4,450,273	4,450,405	7.1/7.9/9.4	21.5/26.4/26.4	*DTH8*	Sakihikari/Nipponbare 188 RILs	Kobayashi and Tomita (2008)
		*qDTH8-2*	aa08002627–aa08004016	SNP	8	10,336,136	14,786,388	3.60/8.51	1.74/5.8		Kanto 209/Koshihikari Aichi SBL RILs	This study
		—	aa08002874	SNP	8	13,057,016	13,057,016	3.69	17.4		Hinohikari/Nikomaru 84 RILs	Kobayashi *et al.* (2015)
QTL_10	FA0603	—	RM5620–RM6673	SSR	10	17,474,848	23,082,406	4.3	12.7	*Ehd1*, *JMJ706*	Koshihikari/Muha BC_4_F_2_	Hori *et al.* (2015)
		—	aa10003607	SNP	10	22,389,675	22,389,675	11.52	19.03	*JMJ706*	Tachisugata/Hokuriku193 191 RILs	Matsubara *et al.* (2016)
		—	RM1162	SSR	10	22,430,482	22,430,582	2.6	43.0	*JMJ706*	Koshihikari/IR64 BC_4_F_2_ (IR64 genetic background)	Nonoue *et al.* (2019)
		—	ad10011436	SNP	10	23,205,372	23,205,372	16.4	17.1	*JMJ706*	Tachisugata/Hokuriku193 402 F_2_	Matsubara *et al.* (2015)
QTL_11	FA1674	*qDTH12*	RM2529	SSR	12	7,567,725	7,567,859	2.23/2.54	8.3/12.2		Moritawase/Koshihikari 92 RILs	Wada *et al.* (2008)
QTL_12	FA1759	—	RM28305–RM5479	SSR	12	19,957,847	24,412,682	4.9	62.5	*OsVIL1*, *spl11*	Koshihikari/Tupa 121-3 BC_4_F_2_ and BC_4_F_3_	Hori *et al.* (2015)
		—	—	—	12	Long arm end		—	—		Tentakaku/Koshihikari ILs (Tentakaku genetic background)	Yamaguchi *et al.* (2018)

*^a^* Chromosome number. *^b^* Physical position of the nearest marker in the ‘Nipponbare’ IRGSP-1.0 genome (Kawahara *et al.* 2013). *^c^* Logarithm of odds. ^†^*P*-values. Values separated by a slash represent multi-year results. *^d^* Percentage of total phenotypic variance explained in each QTL. *^e^* For details on the entries labeled “This study”, see Supplemental Table 7.

**Table 2.** Twenty-nine heading date–related genes targeted for assay development in this study

Gene number	Gene name	Synonym	Classical gene symbol*^a^*	*Hd* gene name*^b^*	Chr.*^c^*	Map position (bp)*^d^*		RAP (Os ID)*^e^*	Transcript ID*^f^*	MSU (LOC_Os ID)*^g^*	Reference
[1]	*OsGI*				1	4,329,725	4,336,437	Os01g0182600	Os01t0182600-01	LOC_Os01g08700.1	Hayama *et al.* (2002)
[2]	*LOC_Os01g62780*	*HESO1*			1	36,354,483	36,358,027	Os01g0846450	Os01t0846450-02	LOC_Os01g62780.1	Yano *et al.* (2016)
[3]	*OsMADS51*	*OsMADS65*			1	40,344,510	40,363,942	Os01g0922800	Os01t0922800-01	LOC_Os01g69850.1	Kim *et al.* (2007)
[4]	*OsVIL2*	*LC2*			2	2,877,244	2,882,018	Os02g0152500	Os02t0152500-01	LOC_Os02g05840.1	Yang *et al.* (2013)
[5]	*OsCOL4*	*OsCCT06*, *OsBBX5*			2	23,989,904	23,990,983	Os02g0610500	Os02t0610500-01	LOC_Os02g39710.1	Lee *et al.* (2010)
[6]	*DTH2*	*OsCCT08*, *OsBBX7*		*Hd7*	2	30,096,306	30,098,580	Os02g0724000	Os02t0724000-01	LOC_Os02g49230.1	Wu *et al.* (2013)
[7]	*Ehd4*				3	717,499	720,181	Os03g0112700	Os03t0112700-02	LOC_Os03g02160.1	Gao *et al.* (2013)
[8]	*Ef-cd*				3	1,270,230	1,271,217	Os03g0122500	Os03t0122500-01	None	Fang *et al.* (2019)
[9]	*OsMADS50*	*DTH3*, *OsSOC1*		*Hd9*	3	1,270,568	1,299,956	Os03g0122600	Os03t0122600-01	LOC_Os03g03070.1 LOC_Os03g03100.1	Lee *et al.* (2004)
[10]	*DTH3b*			*Hd8?*	3	10,478,626	10,482,512	Os03g0298800	Os03t0298800-01	LOC_Os03g18720.1	Chen *et al.* (2015)
[11]	*Hd6*	*OsCKA2*, *CK2α*	*E3*	*Hd6*	3	31,509,001	31,514,176	Os03g0762000	Os03t0762000-02	LOC_Os03g55389.1	Takahashi *et al.* (2001)
[12]	*Hd16*	*EL1*, *CK1*		*Hd16*	3	33,000,435	33,006,539	Os03g0793500	Os03t0793500-01	LOC_Os03g57940.1	Hori *et al.* (2013)
[13]	*OsHDT1*	*HDT701*			5	29,753,821	29,756,231	Os05g0597100	Os05t0597100-01	LOC_Os05g51830.1	Li *et al.* (2011)
[14]	*Hd17*	*OsELF3-1*, *Ef7*	*E2*	*Hd3b*, *Hd17*	6	2,234,581	2,239,058	Os06g0142600	Os06t0142600-01	LOC_Os06g05060.1	Matsubara *et al.* (2012)
[15]	*RFT1*				6	2,927,080	2,928,402	Os06g0157500	Os06t0157500-01	LOC_Os06g06300.1	Ogiso-Tanaka *et al.* (2013)
[16]	*Hd3a*			*Hd3a*	6	2,940,156	2,942,297	Os06g0157700	Os06t0157700-01	LOC_Os06g06320.1	Kojima *et al.* (2002)
[17]	*Hd1*	*OsCCT21*, *OsBBX18*	*Se*, *K*, *Lm*, *Se1*	*Hd1*	6	9,336,535	9,338,359	Os06g0275000	Os06t0275000-01	LOC_Os06g16370.1	Yano *et al.* (2000)
[18]	*Se5*	*OsHY1*, *OsHO1*			6	23,853,944	23,857,724	Os06g0603000	Os06t0603000-01	LOC_Os06g40080.1	Izawa *et al.* (2000)
[19]	*OsFTIP1*				6	24,555,499	24,557,973	Os06g0614000	Os06t0614000-01	LOC_Os06g41090.1	Song *et al.* (2017)
[20]	*Ghd7*	*EH7*, *OsCTT26*, *EH7-1*	*E1*, *M*, *m-Ef1*	*Hd4*	7	9,152,402	9,154,820	Os07g0261200	Os07t0261200-01	LOC_Os07g15770.1	Xue *et al.* (2008)
[21]	*OsMADS18*				7	24,788,565	24,793,430	Os07g0605200	Os07t0605200-01	LOC_Os07g41370.1	Fornara *et al.* (2004)
[22]	*PRR37*	*DTH7*, *Ghd7.1*, *EH7-2*, *OsCCT28*		*Hd2*	7	29,617,430	29,628,600	Os07g0695100	Os07t0695100-01	LOC_Os07g49460.1	Koo *et al.* (2013)
[23]	*Ehd3*				8	273,583	276,691	Os08g0105000	Os08t0105000-01	LOC_Os08g01420.1	Matsubara *et al.* (2011)
[24]	*Hd18*			*Hd18*	8	2,385,914	2,389,226	Os08g0143400	Os08t0143400-01	LOC_Os08g04780.1	Shibaya *et al.* (2016)
[25]	*OsLHY*	*OsCCA1*			8	3,366,559	3,373,116	Os08g0157600	Os08t0157600-01	LOC_Os08g06110.2	Ogiso *et al.* (2010)
[26]	*DTH8*	*Ghd8*, *LHD1*, *LH8*, *OsHAP3H*		*Hd5*	8	4,333,846	4,334,739	Os08g0174500	Os08t0174500-01	LOC_Os08g07740.1	Wei *et al.* (2010)
[27]	*Ehd2*	*OsId1*, *RID1*, *Ghd10*			10	14,739,897	14,742,943	Os10g0419200	Os10t0419200-01	LOC_Os10g28330.1	Matsubara *et al.* (2008)
[28]	*Ehd1*		*E*, *Ef1*	*Hd14*	10	17,076,098	17,081,344	Os10g0463400	Os10t0463400-01	LOC_Os10g32600.1	Doi *et al.* (2004)
[29]	*LOC_Os11g08410*	*OsGATA28*			11	4,432,776	4,434,071	Os11g0187200	Os11t0187200-01	LOC_Os11g08410.1	Yano *et al.* (2016)

*^a^* Hori *et al.* (2016). *^b^* *Hd1*–*Hd14*: Yano *et al.* (2001); *Hd3a*–*Hd3b*: Monna *et al.* (2002); *Hd15*: Ogiso-Tanaka *et al.* (2017); *Hd16*: Hori *et al.* (2013); *Hd17*: Matsubara *et al.* (2012); *Hd18*: Shibaya *et al.* (2016). *^c^* Chromosome number. *^d^* Coding sequence positions for the ‘Nipponbare’ allele based on the ‘Nipponbare’ IRGSP-1.0 reference genome (Kawahara *et al.* 2013). ^†^ This range is based on the functional ‘Kasalath’ allele (accession number: AB036786.1), because the ‘Nipponbare’ allele, Os03t0762000-02, has a premature stop codon (Takahashi *et al.* 2001). *^e^* Rice Annotation Project: https://rapdb.dna.affrc.go.jp/ (Sakai *et al.* 2013). *^f^* These transcript variants were used in this study as representative of gene structure. *^g^* Michigan State University Rice Genome Annotation Project (http://rice.uga.edu/).
