# Supplementary material for: Development of SNP genotyping assays for heading date in rice
Source: Breed Sci. 2024 Jun 25;74(3):274–84. doi: 10.1270/jsbbs.23093 (PMC11561416; doi:10.1270/jsbbs.23093)
Supplement: Supplementary file 1 — Supplemental Figures [file 74_274-s1.pdf]

doi: 10.1270/jsbbs.23093

Supplemental Fig. 1. QTL analysis for days-to-heading in recombinant inbred lines (RILs) (2011_RILs and 2012_RILs) derived from a cross between ‘Kanto 209’ (K209) and ‘Koshihikari Aichi SBL’ (KoASBL) in the field in 2011 and 2012.
(A) LOD curves obtained by composite interval mapping (CIM) and LOD peaks above the threshold. SNP markers on each chromosome are plotted along the horizontal axis according to the genetic map (cM). Horizontal dashed lines show the LOD threshold at the 5% significance level calculated from 1000 permutation tests and the fixed LOD threshold of 3. Arrows indicate QTL positions; in 2012_RILs, only *qDTH6* was incorporated into the QTL model by multiple interval mapping (MIM). The gene (red) and QTLs (blue) targeted in this study are indicated with serial numbers. The lower panel shows the details of the neighboring QTL, *qDTH8-2*, which was genetically dissected from the major QTL, *qDTH8-1* (*Hd18* gene). Dotted LOD peaks were determined by MIM. The details of each QTL are described in Supplemental Table 7.
(B) Frequency distributions of days-to-heading for the genotype of aa08000774, the SNP marker closest to the major QTL *qDTH8-1*. Triangles show locations of the means for the KoASBL-homozygous (black), heterozygous (hatched), and K209-homozygous (white) types.
(C) Boxplots of days-to-heading by the genotypes of the aa08000727 and aa08004016 markers closest to the two main QTLs on chromosome 8, *qDTH8-1* and *qDTH8-2*, respectively. +, the mean value. The same lowercase letters indicate no statistically significant differences among the means (Tukey’s HSD at the 5% significance level).

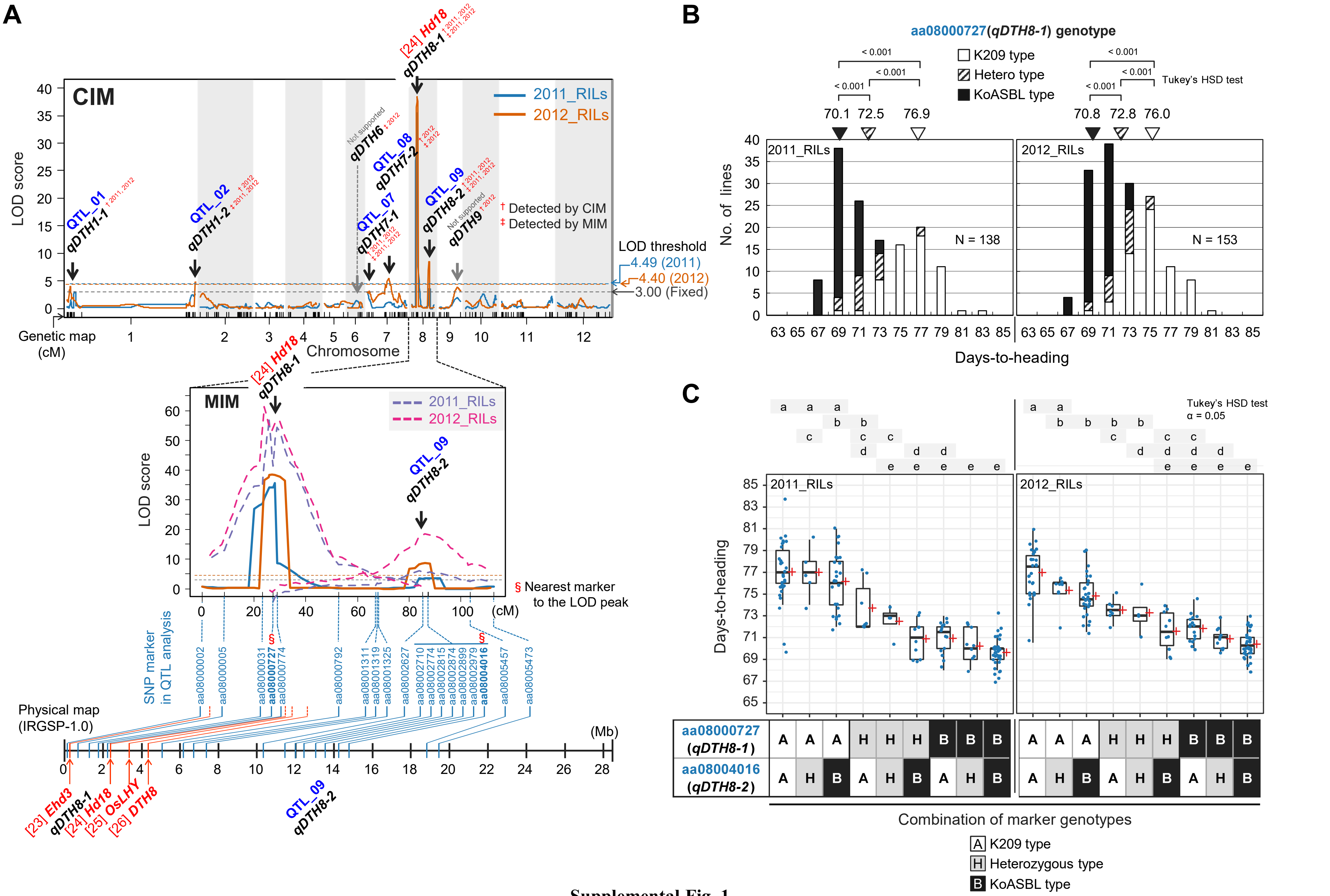

Supplemental Fig. 1

Supplemental Fig. 2. Gene structure, mutation sites used for allele discrimination, and the corresponding assays. Gene names and numbers are as in Table 2. Black boxes, coding sequences (CDSs). Lines between CDSs, introns; lines outside the CDS, the 5′ and 3′ untranslated regions. White boxes with gray outlines in the *Hd6* allele ([11]), the 'Kasalath' CDS with no premature stop codon (functional allele). Start, the translation start site (ATG); stop, stop codon; orange arrows, the orientation of the gene relative to the 'Nipponbare' IRGSP-1.0 reference genome (Kawahara *et al.* 2013, Sakai *et al.* 2013). Above the CDS, information on the mutation targeted for assay development is indicated together with the base number. FNP, mutations with confirmed functionality (also see Supplemental Table 4). Triangles depict ▽ insertions and ▲ deletions (roughly to size). Below the CDS, assays used to discriminate alleles are shown. NA (not available), direct assay for the mutation was unsuccessful.

Supplemental Literature Cited

Kim, S.R., G. Torollo, M.R. Yoon, J. Kwak, C.K. Lee, G.D. Prahalada, I.R. Choi, U.S. Yeo, O.Y. Jeong, K.K. Jena *et al.* (2018) Loss-of-function alleles of *heading date 1* (*Hd1*) are associated with adaptation of temperate *japonica* rice plants to the tropical region. Front Plant Sci 9: 1827.

Yan, W.H., P. Wang, H.X. Chen, H.J. Zhou, Q.P. Li, C.R. Wang, Z.H. Ding, Y.S. Zhang, S.B. Yu, Y.Z. Xing *et al.* (2011) A major QTL, *Ghd8*, plays pleiotropic roles in regulating grain productivity, plant height, and heading date in rice. Mol Plant 4: 319–330.

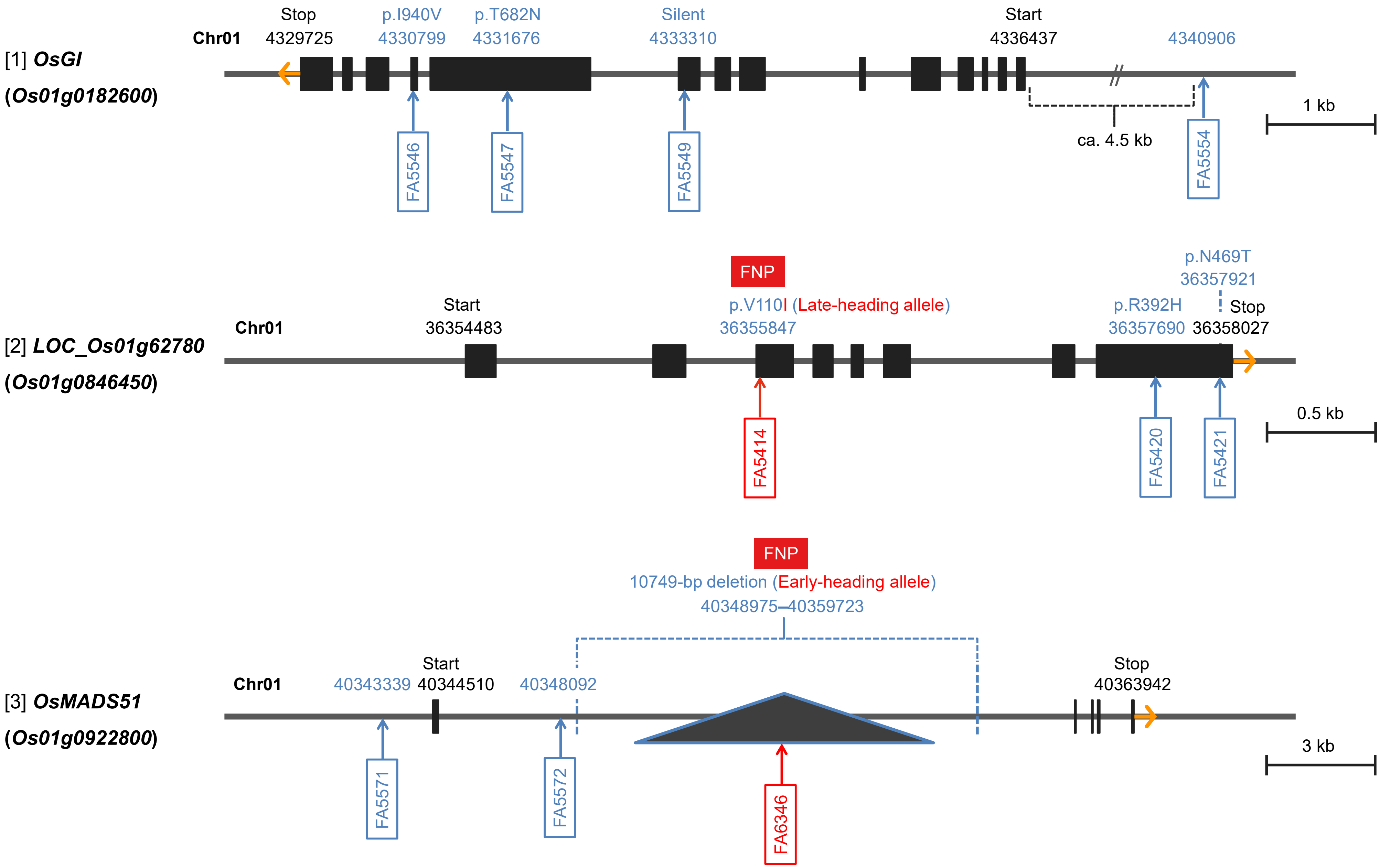

Supplemental Fig. 2

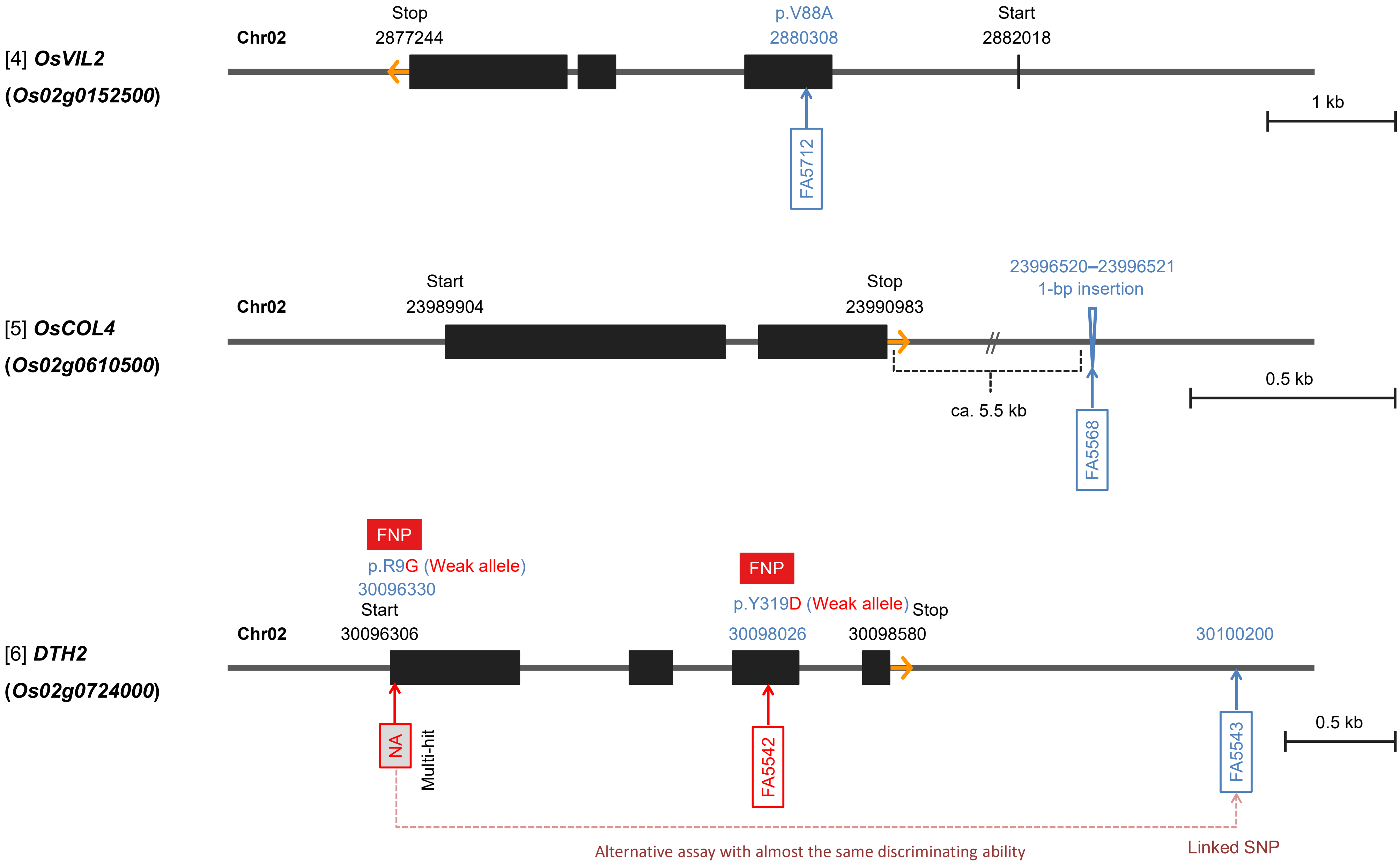

Supplemental Fig. 2 (Continued)

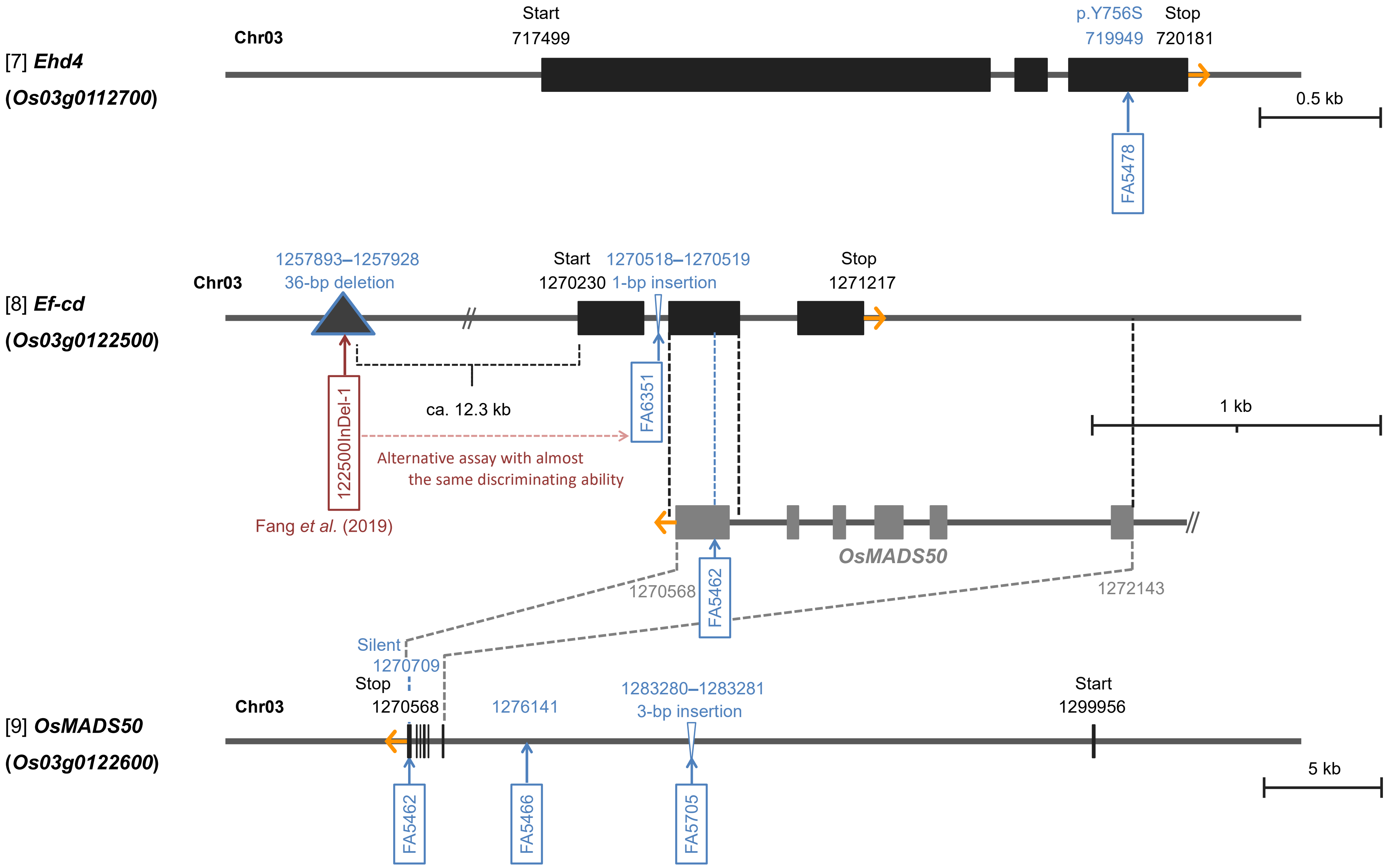

Supplemental Fig. 2 (Continued)

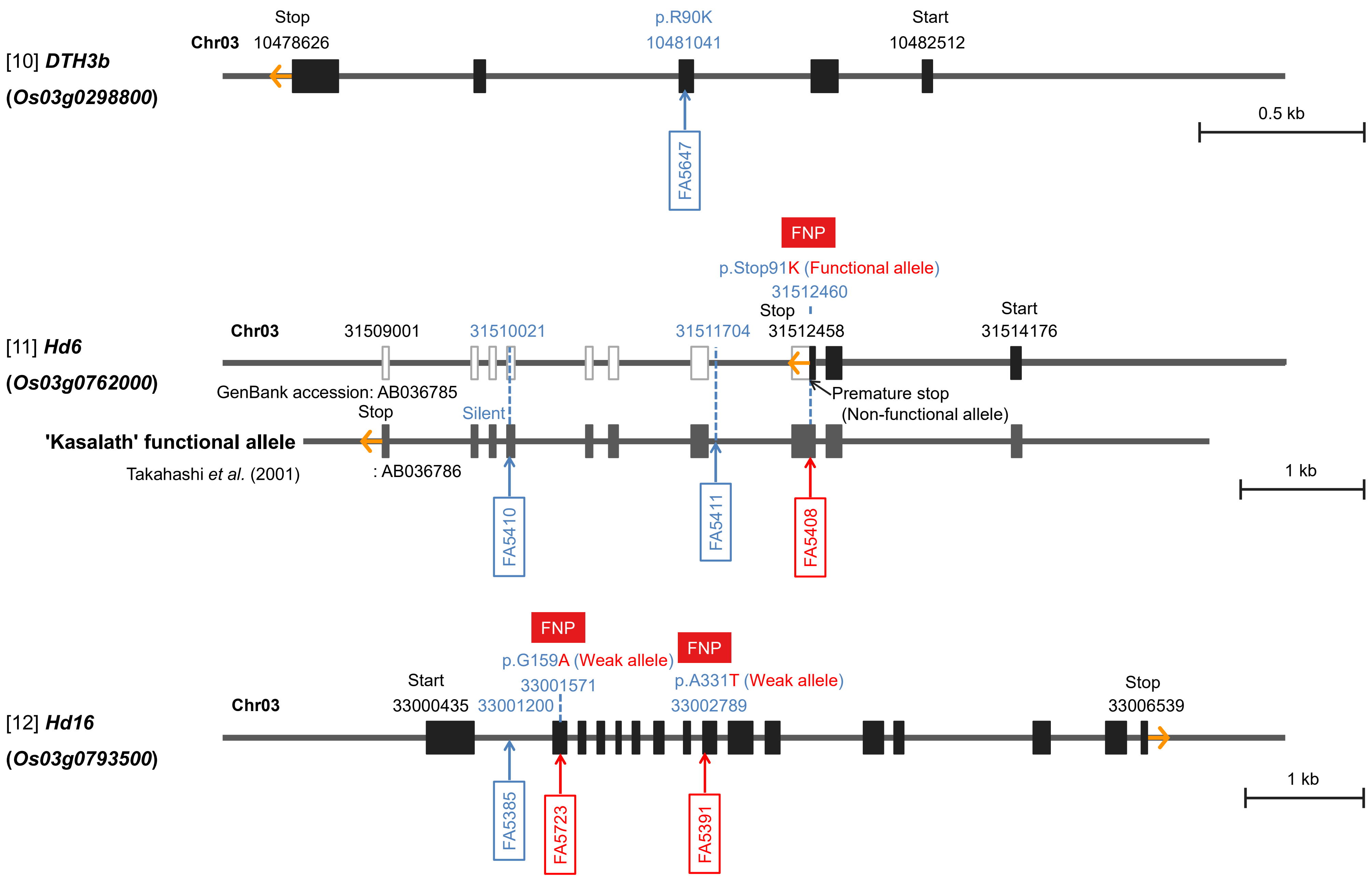

Supplemental Fig. 2 (Continued)

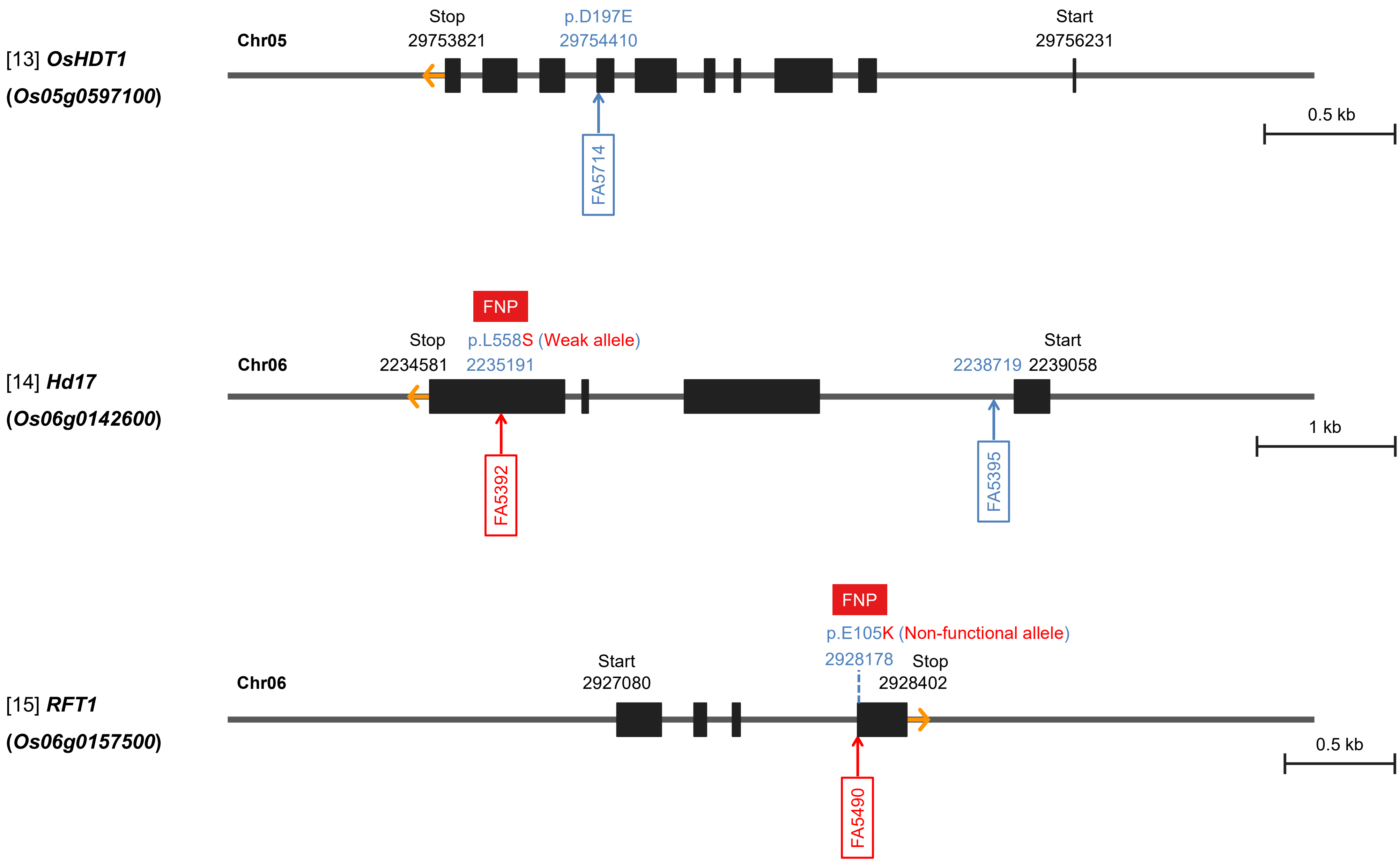

Supplemental Fig. 2 (Continued)

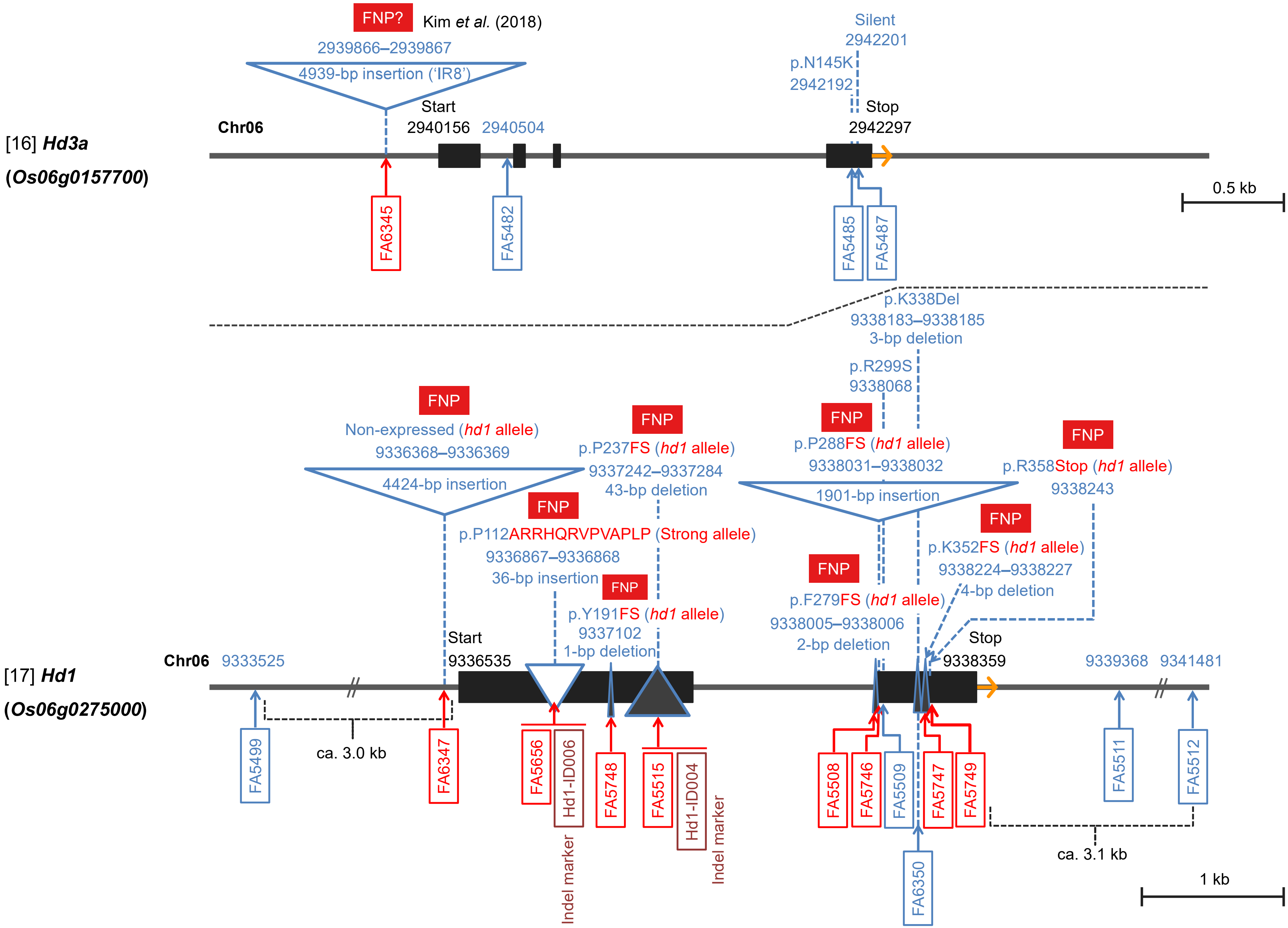

Supplemental Fig. 2 (Continued)

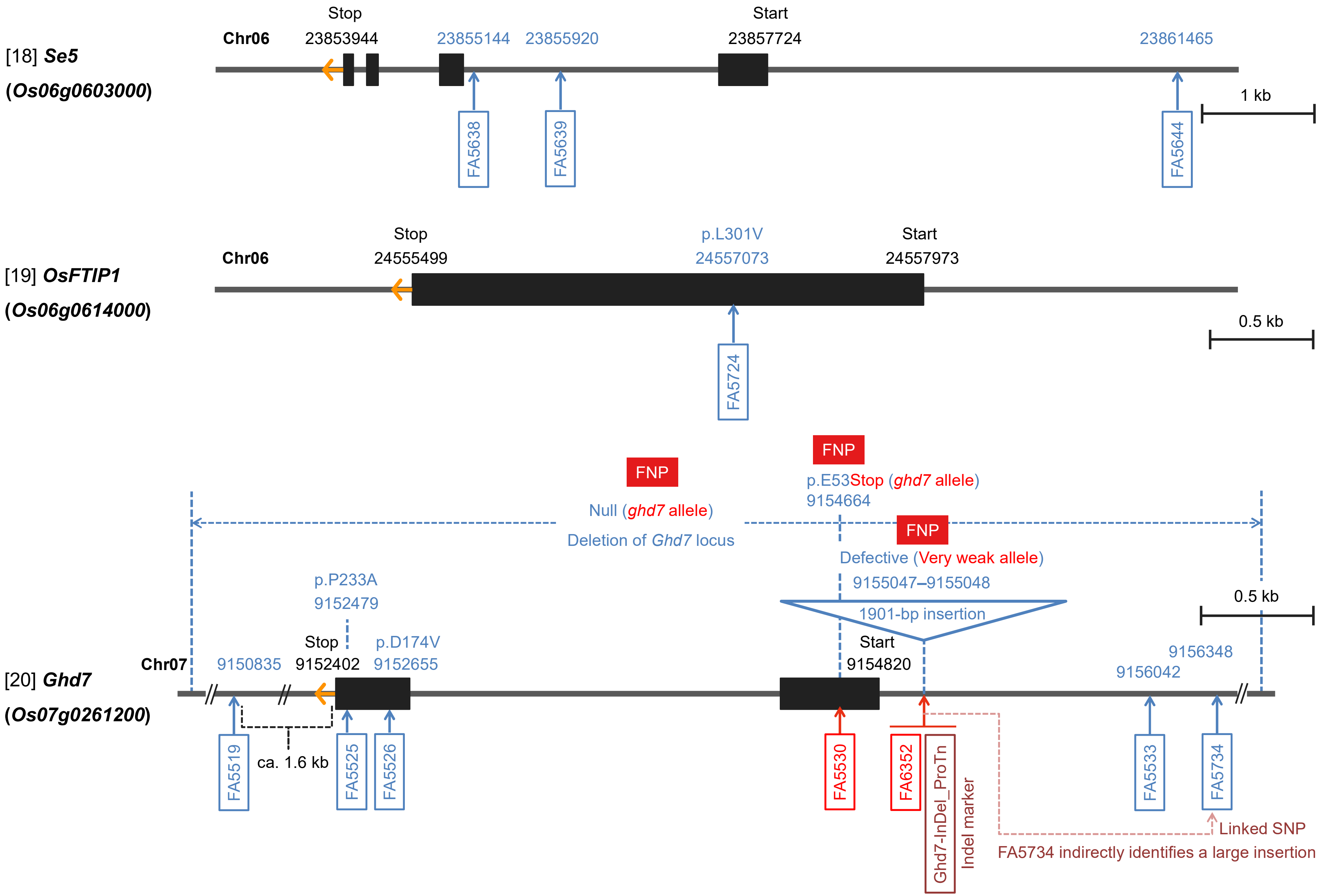

Supplemental Fig. 2 (Continued)

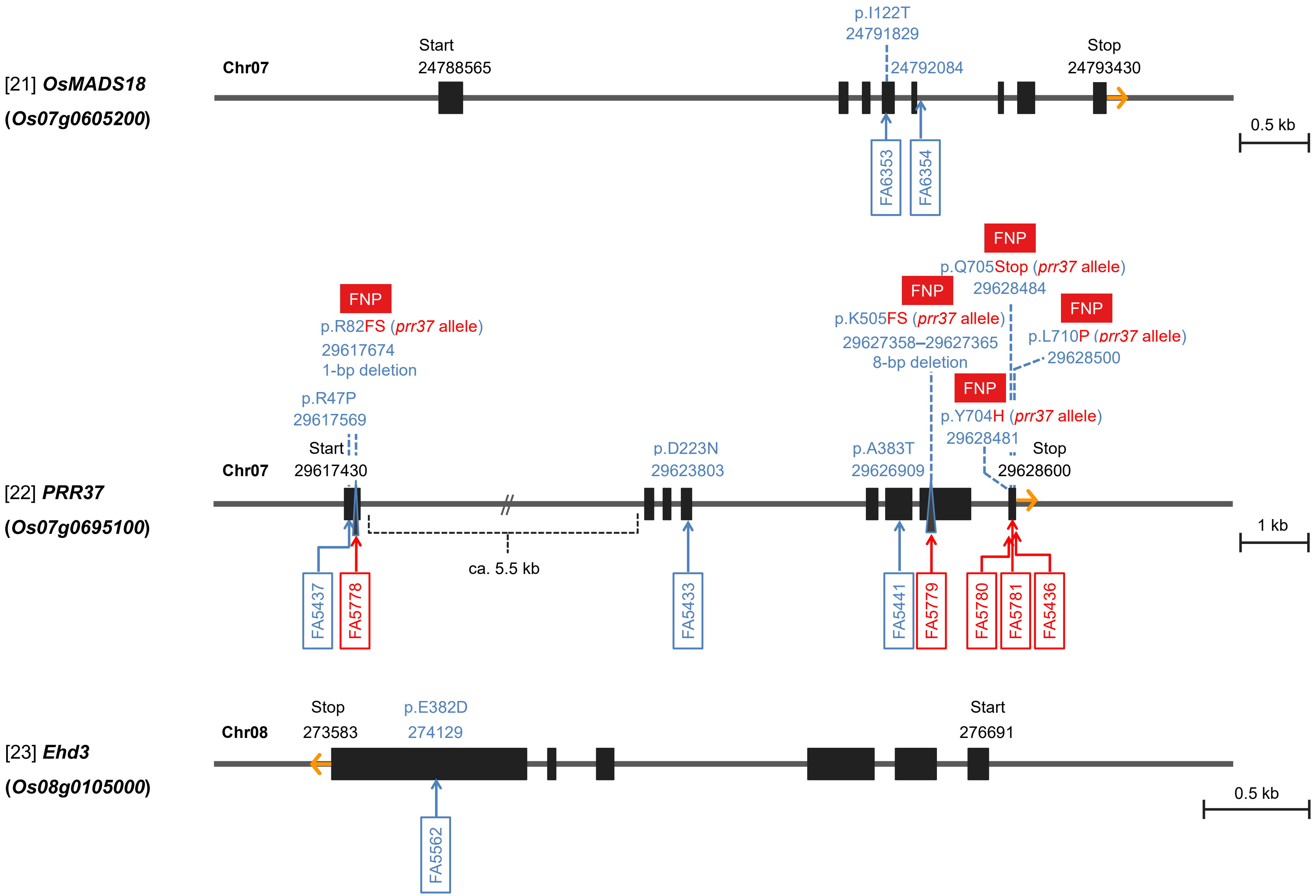

Supplemental Fig. 2 (Continued)

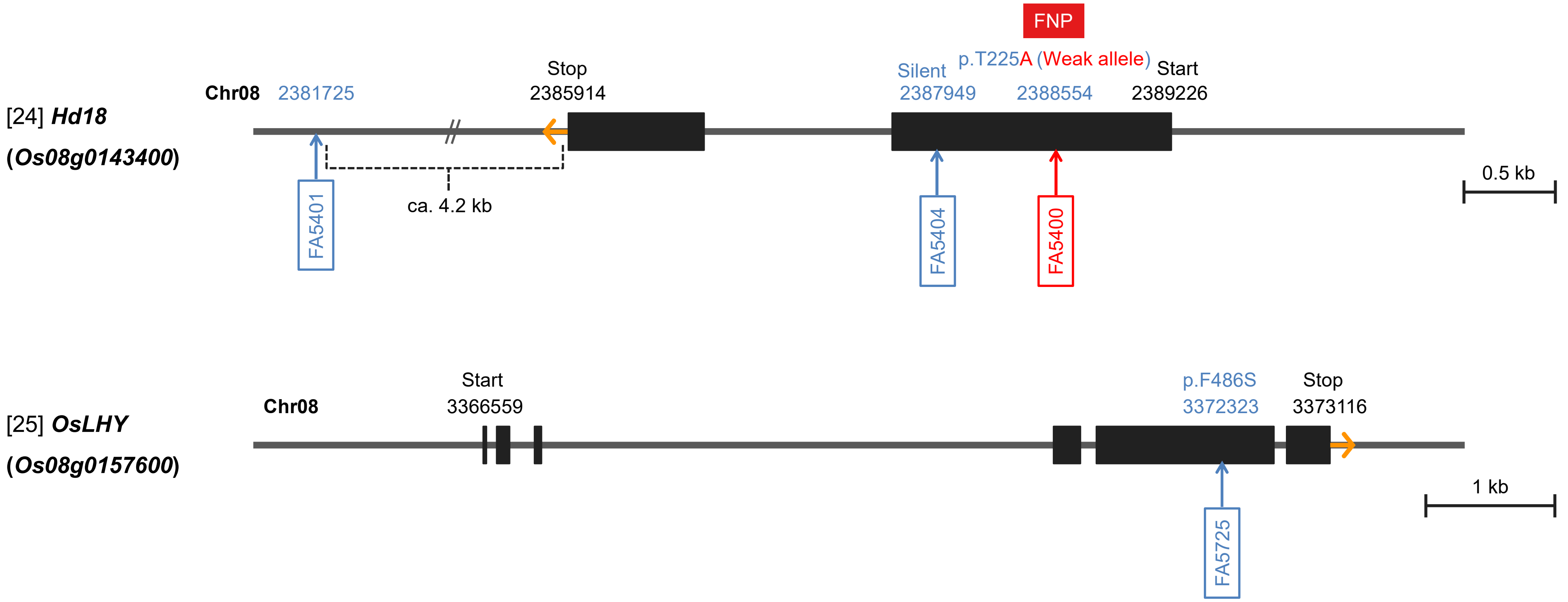

Supplemental Fig. 2 (Continued)

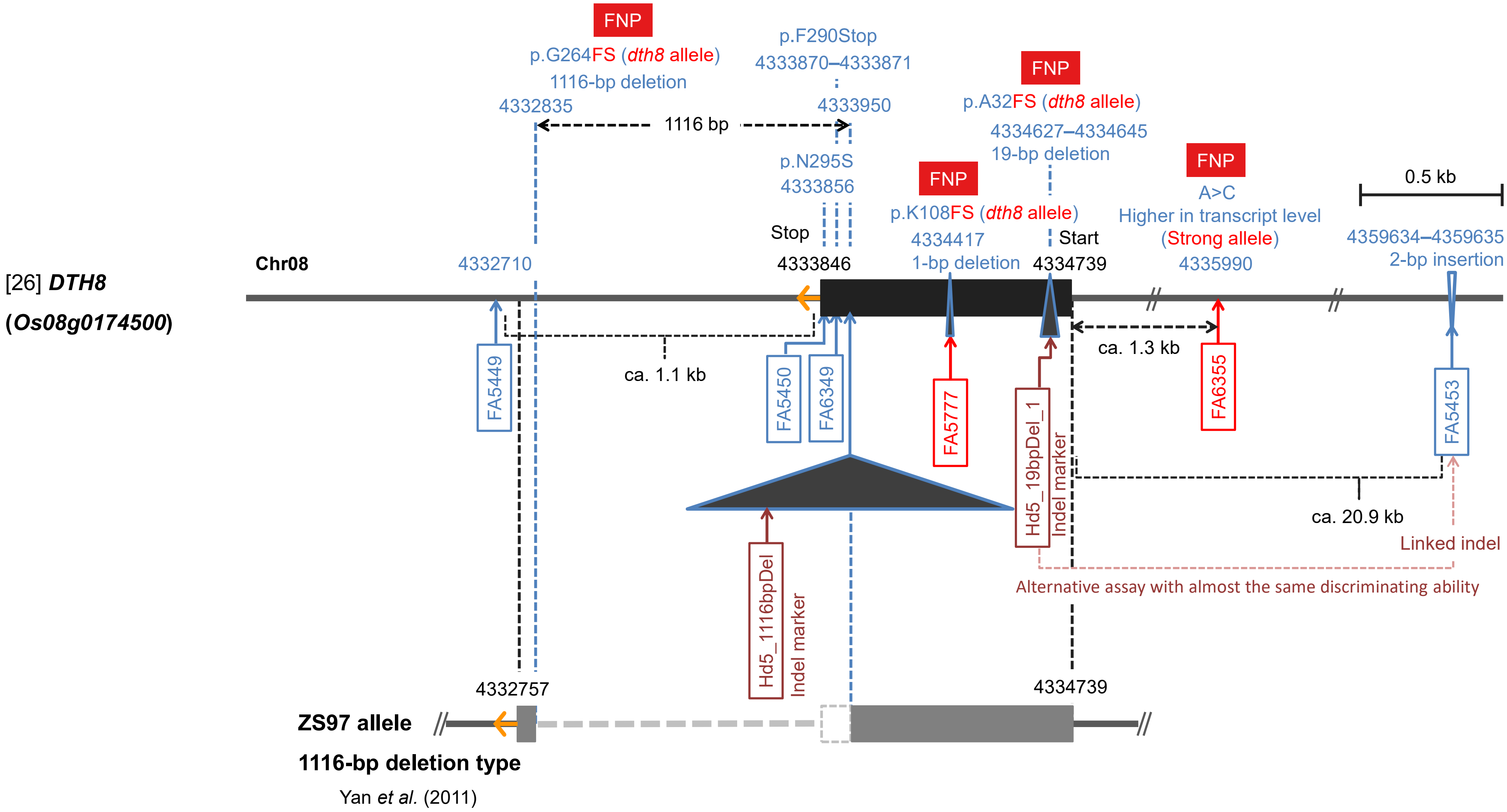

Supplemental Fig. 2 (Continued)

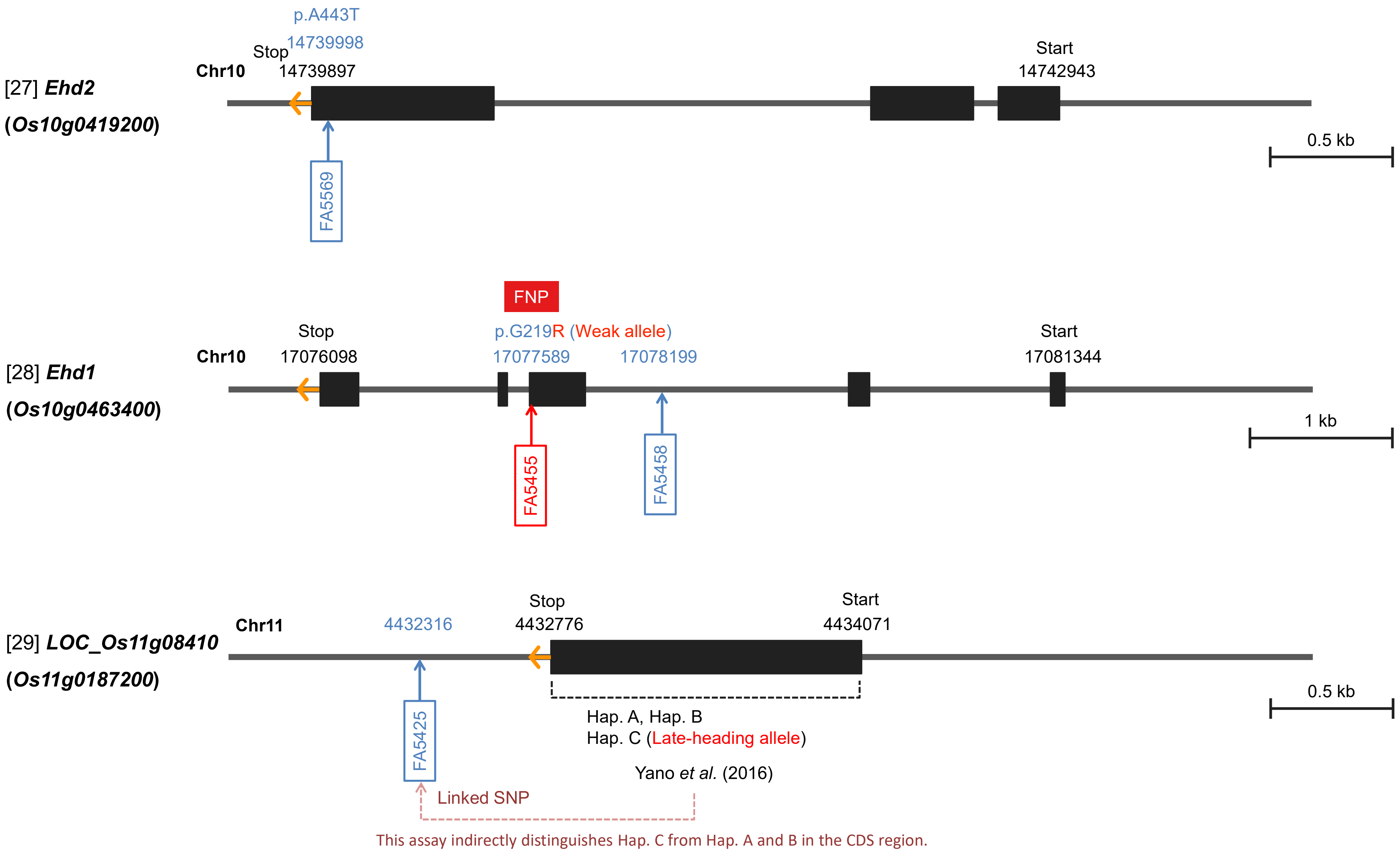

Supplemental Fig. 2 (Continued)

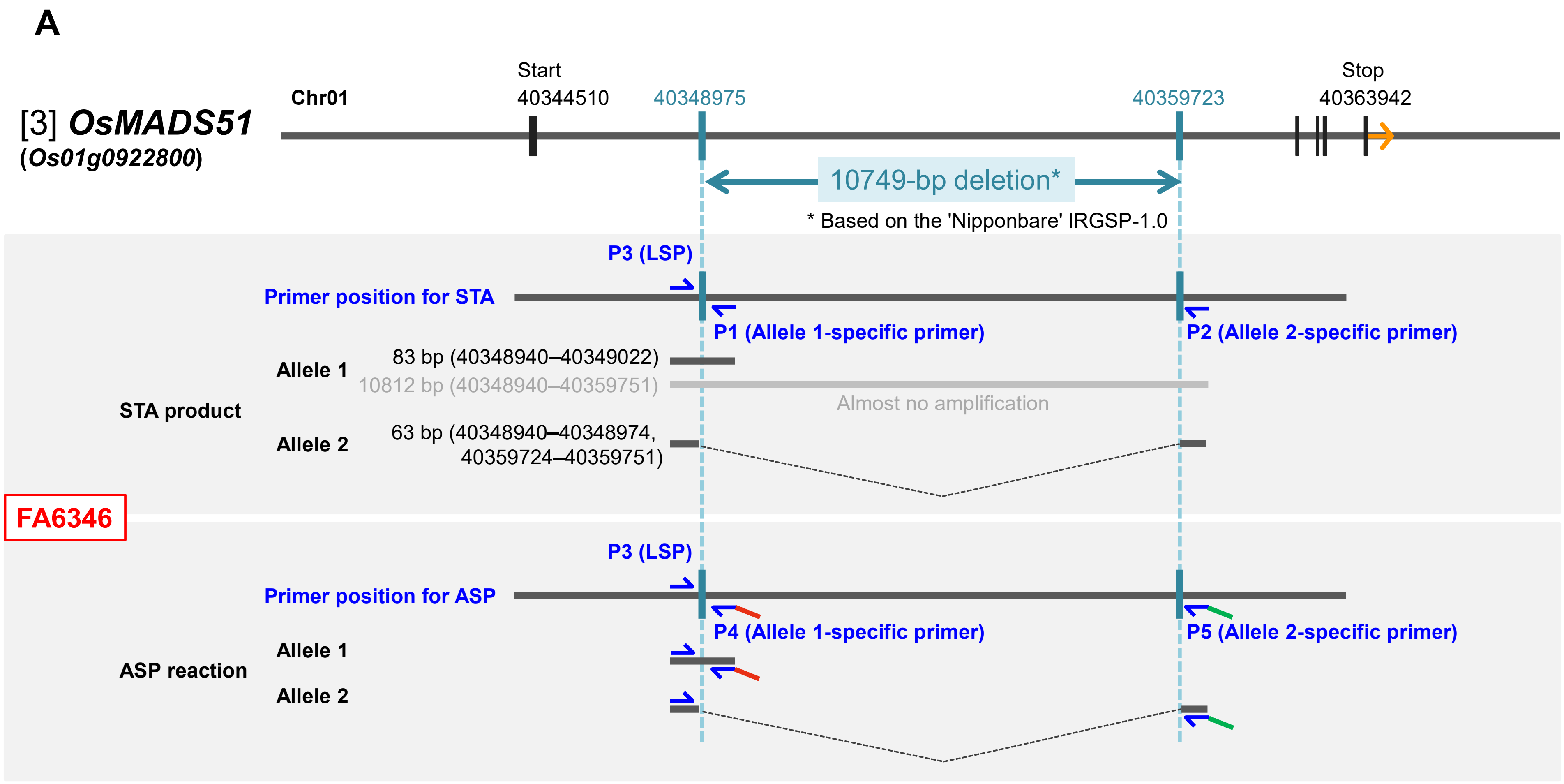

Supplemental Fig. 3. Details of three assays (FA6346, FA6345, and FA6347) designed to detect polymorphisms with a large insertion or deletion. Colored lines at the 5′ ends of allele-specific primers (ASPs) indicate the tag sequences for fluorescent labeling (red, FAM; green, HEX). In each assay, specific target amplification (STA) reaction is performed with the primers P1, P2, and P3 mixed at a 1:1:2 ratio; the concentration of P3 is adjusted to the standard locus-specific primer (LSP) concentration. The ASP reaction with the primers P4, P5, and P3 is performed under standard conditions with standard mixing ratios (see Materials and Methods). (A) FA6346 assay to detect a 10749-bp deletion at the *OsMADS51* locus. (B) FA6345 assay to detect a 4939-bp insertion at the *Hd3a* locus. (C) FA6347 assay to detect a 4424-bp insertion at the *Hd1* locus.

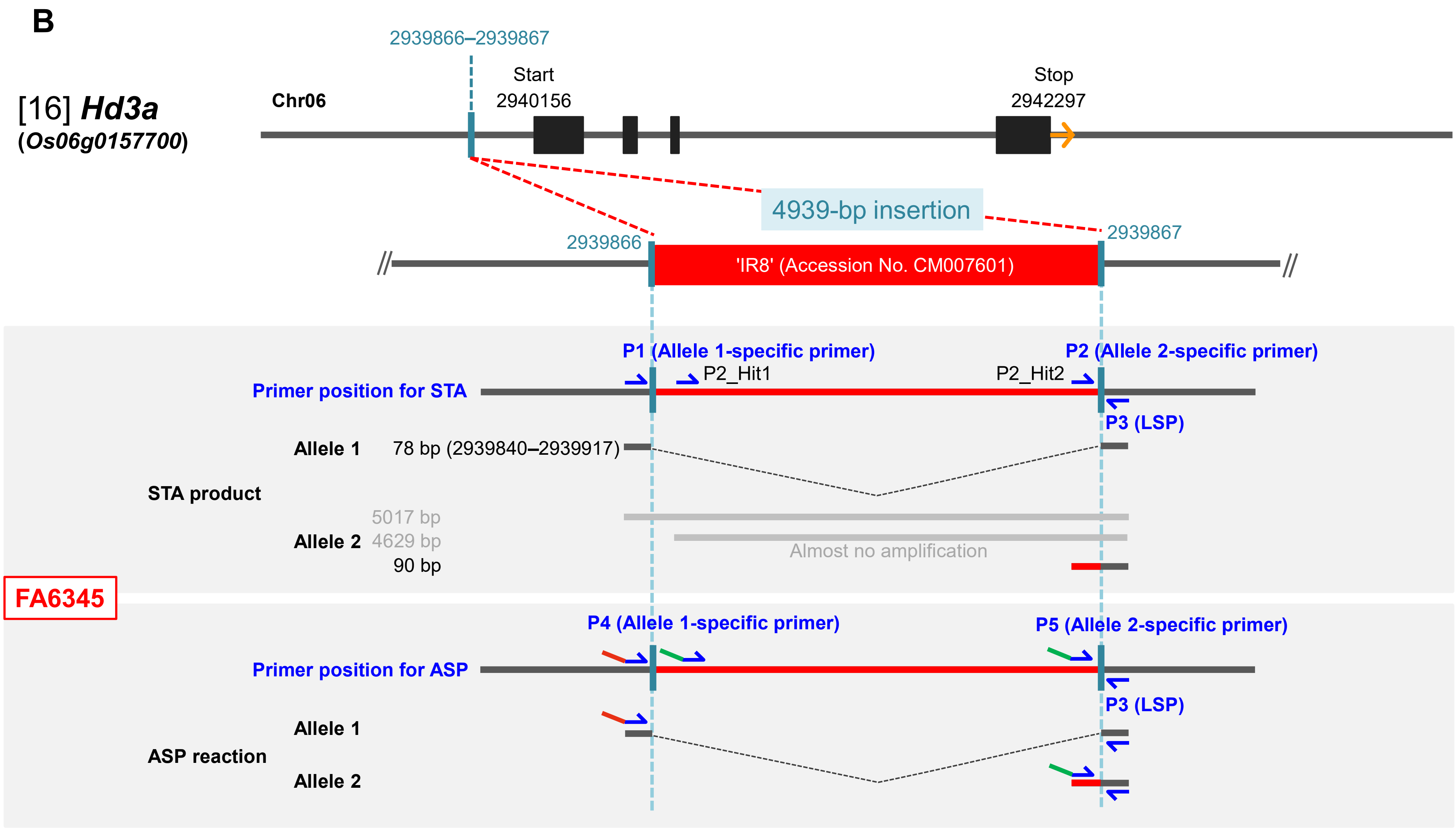

Supplemental Fig. 3 (Continued)

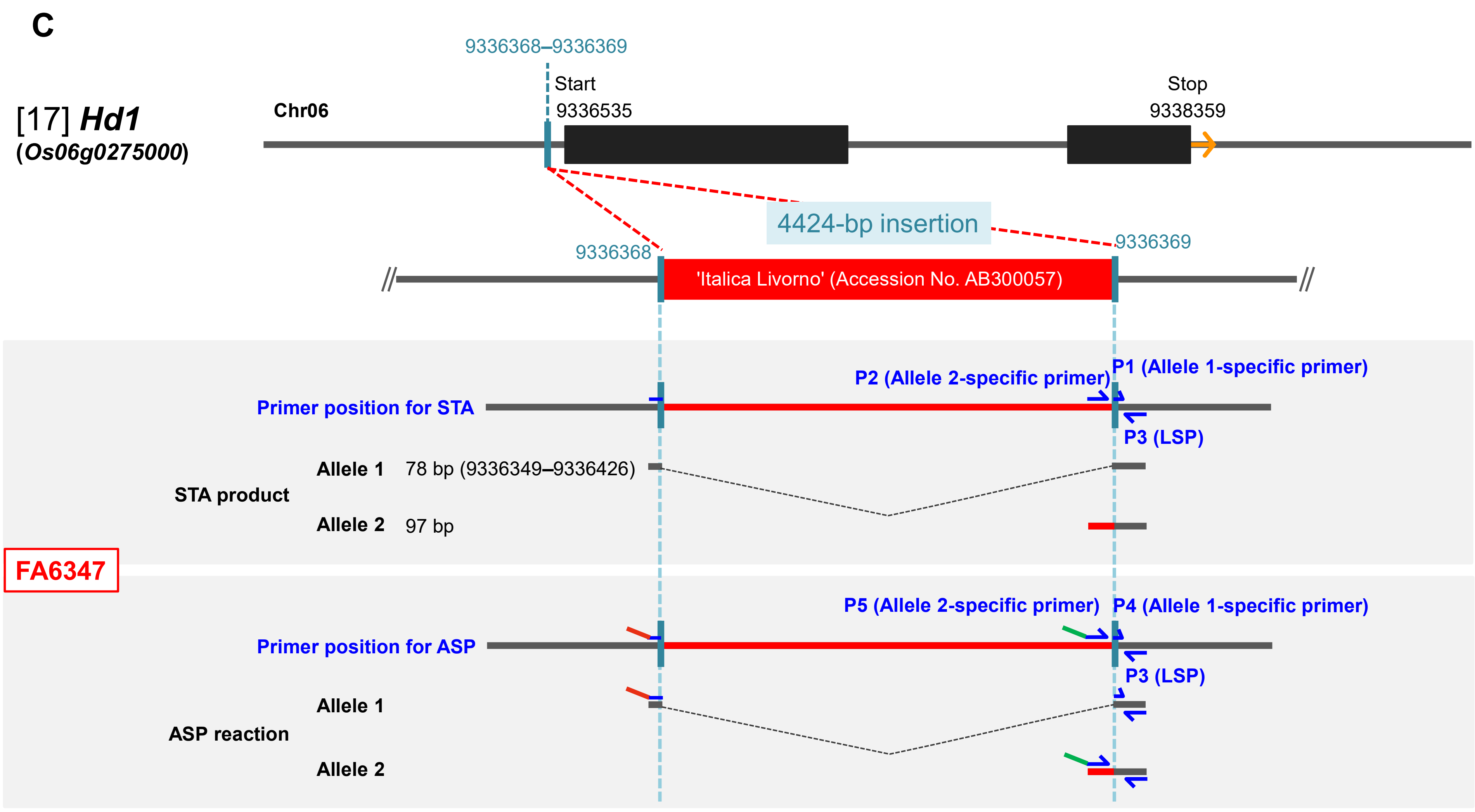

Supplemental Fig. 3 (Continued)

Supplemental Fig. 4. Alleles of 377 cultivars identified in the 96-plex SNP genotyping assays (HDA1). Allele types shown on the left: A, marker genotype of 'Nipponbare'; B, other marker genotypes; −, NoCall (no elevated fluorescence signal). At all loci, the allele number (Allele No.) was set to A01 for the 'Nipponbare' type. On the right, the assays for each locus and information on the corresponding mutation are shown. FA serial No. corresponds to that in the list of 96-plex SNP genotyping assays (Supplemental Table 8). Black background indicates mutations in the coding sequence (CDS). Red assay names indicate FNPs shown in Supplemental Table 4. Allele types shown on the right: green, 'Nipponbare'; yellow, other; white, NoCall; red, type that contributes to non-functionality. When allele type ≥ 11 bp, the specific base number is indicated. Chr., chromosome number; AA sub, amino acid substitution; INS, insertion; DEL, deletion.

Locus / Gene [1]	Allele	Allele No.	Chr.	1			
			IRGSP-1.0 position (bp)	4,330,799	4,331,676	4,333,310	4,340,906
			CDS position	2,818	2,045	1,209	5' region
			AA sub	I940V	T682N	Silent	-
			FA serial No.	1	2	3	4
			Assay name	FA5546	FA5547	FA5549	FA5554
OsGI	AAAA	A01	Allele type	T	G	C	T
	BAAA	A02		C	G	C	T
	AAAB	A03		T	G	C	C
	BBAA	A04		C	T	C	T
	BBBA	A05		C	T	T	T

Locus / QTL_01	Allele	Allele No.	Chr.	1
			IRGSP-1.0 position (bp)	5,590,233
			CDS position	-
			AA sub	-
			FA serial No.	5
			Assay name	FA0057
**FA0057**	A	A01	Allele type	G
	B	A02		A
	-	A03		-

Locus / Gene [2]	Allele	Allele No.	Chr.	1		
			IRGSP-1.0 position (bp)	36,355,847	36,357,690	36,357,921
			CDS position	328	1,175	1,406
			AA sub	V110I	R392H	N469T
			FA serial No.	6	7	8
			Assay name	FA5414	FA5420	FA5421
LOC_Os01g62780	AAA	A01	Allele type	G	G	A
	BAA	A02		A	G	A
	BAB	A03		A	G	C
	BBB	A04		A	A	C

Supplemental Fig. 4

Locus	Allele	Allele No.		
			Chr.	1
			IRGSP-1.0 position (bp)	37,507,621
			CDS position	-
			AA sub	-
QTL_02			FA serial No.	9
			Assay name	FA0118
**FA0118**	A	A01	Allele type	C
	B	A02		T

Locus	Allele	Allele No.				
			Chr.	1		
			IRGSP-1.0 position (bp)	40,343,339	40,348,092	40,348,975–40,359,723
			CDS position	5' region	Intron	Intron
			AA sub	-	-	-
Gene [3]			FA serial No.	10	11	12
			Assay name	FA5571	FA5572	FA6346
OsMADS51	AAA	A01	Allele type	A	A	10749bp
	BAA	A02		G	A	10749bp
	BAB	A03		G	A	10749bp DEL
	BBA	A04		G	C	10749bp

Locus	Allele	Allele No.		
			Chr.	2
			IRGSP-1.0 position (bp)	2,880,308
			CDS position	263
			AA sub	V88A
Gene [4]			FA serial No.	13
			Assay name	FA5712
OsVIL2	A	A01	Allele type	A
	B	A02		G

Supplemental Fig. 4 (Continued)

Locus	Allele	Allele No.
QTL_03		
**FA2420**	A	A01
	B	A02

Chr.	2
IRGSP-1.0 position (bp)	11,579,446
CDS position	-
AA sub	-
FA serial No.	14
Assay name	FA2420
Allele type	T
	G

Locus	Allele	Allele No.
Gene [5]		
OsCOL4	A	A01
	B	A02

Chr.	2
IRGSP-1.0 position (bp)	23,996,520–23,996,521
CDS position	3' region
AA sub	-
FA serial No.	15
Assay name	FA5568
Allele type	DEL
	A

Locus	Allele	Allele No.
Gene [6]		
DTH2	AA	A01
	BA	A02
	BB	A03

Chr.	2	
IRGSP-1.0 position (bp)	30,098,026	30,100,200
CDS position	955	3' region (25)
AA sub	Y319D	- (Alternative to R9G)
FA serial No.	16	17
Assay name	FA5542	FA5543
Allele type	T	A
	G	A
	G	G

Supplemental Fig. 4 (Continued)

Locus / Gene [7]	Allele	Allele No.		
			Chr.	3
			IRGSP-1.0 position (bp)	719,949
			CDS position	2,267
			AA sub	Y756S
			FA serial No.	18
			Assay name	FA5478
Ehd4	A	A01	Allele type	A
	B	A02		C

Locus / Gene [8]	Allele	Allele No.			Indel marker genotype
			Chr.	3	3
			IRGSP-1.0 position (bp)	1,270,518–1,270,519	1,257,893–1,257,928
			CDS position	Intron	5' region
			AA sub	-	-
			FA serial No.	19	-
			Assay name	FA6351	122500InDel-1
Ef-cd	A	A01	Allele type	DEL	36bp
	B	A02		T	36bp DEL

Locus / Gene [9]	Allele	Allele No.				
			Chr.	3		
			IRGSP-1.0 position (bp)	1,270,709	1,276,141	1,283,280–1,283,281
			CDS position	552	Intron	Intron
			AA sub	Silent	-	-
			FA serial No.	20	21	22
			Assay name	FA5462	FA5466	FA5705
OsMADS50	AAA	A01	Allele type	G	G	DEL
	AAB	A02		G	G	TTC
	BAB	A03		A	G	TTC
	BBB	A04		A	T	TTC
	B--	A05		A	-	-

Supplemental Fig. 4 (Continued)

Locus	Allele	Allele No.		
QTL_04			Chr.	3
			IRGSP-1.0 position (bp)	1,763,199
			CDS position	-
			AA sub	-
			FA serial No.	23
			Assay name	FA0192
**FA0192**	A	A01	Allele type	G
	B	A02		A

Locus	Allele	Allele No.		
QTL_05			Chr.	3
			IRGSP-1.0 position (bp)	2,196,417
			CDS position	-
			AA sub	-
			FA serial No.	24
			Assay name	FA0193
**FA0193**	A	A01	Allele type	C
	B	A02		T
	-	A03		-

Locus	Allele	Allele No.		
Gene [10]			Chr.	3
			IRGSP-1.0 position (bp)	10,481,041
			CDS position	269
			AA sub	R90K
			FA serial No.	25
			Assay name	FA5647
DTH3b	A	A01	Allele type	C
	B	A02		T

Supplemental Fig. 4 (Continued)

Locus / Gene [11]	Allele	Allele No.	Chr.	3		
			IRGSP-1.0 position (bp)	31,510,021	31,511,704	31,512,460
			CDS position	3' region (792)†	3' region (Intron)†	271
			AA sub	- (Silent)†	-	Stop91K
			FA serial No.	26	27	28
			Assay name	FA5410	FA5411	FA5408
Hd6	AAA	A01	Allele type	A	T	A
	AAB	A02		A	T	T
	ABB	A03		A	G	T
	BAB	A04		G	T	T

† Information in parentheses is based on the functional alleles of the 'Kasalath' type (see Supplemental Fig. 2).

Locus / Gene [12]	Allele	Allele No.	Chr.	3		
			IRGSP-1.0 position (bp)	33,001,200	33,001,571	33,002,789
			CDS position	Intron	476	991
			AA sub	-	G159A	A331T
			FA serial No.	29	30	31
			Assay name	FA5385	FA5723	FA5391
Hd16	AAA	A01	Allele type	C	G	G
	BAA	A02		A	G	G
	AAB	A03		C	G	A
	ABA	A04		C	C	G

Locus / QTL_06	Allele	Allele No.	Chr.	5
			IRGSP-1.0 position (bp)	75,314
			CDS position	-
			AA sub	-
			FA serial No.	32
			Assay name	FA1067
**FA1067**	A	A01	Allele type	C
	B	A02		G

Supplemental Fig. 4 (Continued)

Locus / Gene [13]	Allele	Allele No.	Chr.	5
			IRGSP-1.0 position (bp)	29,754,410
			CDS position	591
			AA sub	D197E
			FA serial No.	33
			Assay name	FA5714
OsHDT1	A	A01	Allele type	A
	B	A02		T

Locus / Gene [14]	Allele	Allele No.	Chr.	6	
			IRGSP-1.0 position (bp)	2,235,191	2,238,719
			CDS position	1,673	-
			AA sub	L558S	-
			FA serial No.	34	35
			Assay name	FA5392	FA5395
Hd17	AA	A01	Allele type	A	G
	BA	A02		G	G
	BB	A03		G	A

Locus / Gene [15]	Allele	Allele No.	Chr.	6
			IRGSP-1.0 position (bp)	2,928,178
			CDS position	313
			AA sub	E105K
			FA serial No.	36
			Assay name	FA5490
RFT1	A	A01	Allele type	G
	B	A02		A

Supplemental Fig. 4 (Continued)

Locus / Gene [16]	Allele	Allele No.					
Locus			Chr.	6			
			IRGSP-1.0 position (bp)	2,939,866–2,939,867	2,940,504	2,942,192	2,942,201
			CDS position	5' region	Intron	435	444
			AA sub	-	-	N145K	Silent
			FA serial No.	37	38	39	40
Gene [16]			Assay name	FA6345	FA5482	FA5485	FA5487
Hd3a	AAAA	A01	Allele type	DEL	T	C	C
	ABAA	A02		DEL	G	C	C
	AABA	A03		DEL	T	G	C
	AAAB	A04		DEL	T	C	T
	BBAA	A05		4939bp INS	G	C	C

Locus / Gene [17]	Allele	Allele No.														
Locus			Chr.	6												
			IRGSP-1.0 position (bp)	9,333,525	9,336,368–9,336,369	9,336,867–9,336,868	9,337,102	9,337,242–9,337,284	9,338,005–9,338,006	9,338,031–9,338,032	9,338,068	9,338,183–9,338,185	9,338,224–9,338,227	9,338,243	9,339,368	9,341,481
			CDS position	5' region	5' region	333–334	568	708–750	835–836	860–861	897	1,012–1,014	1,053–1,056	1,072	3' region	3' region
			AA sub	-	-	P112ARRHQRVPVAPLP	Y191FS	P237FS	F279FS	P288FS	R299S	K338DEL	K352FS	R358Stop	-	-
			FA serial No.	41	42	43	44	45	46	47	48	49	50	51	52	53
Gene [17]			Assay name	FA5499	FA6347	FA5656	FA5748	FA5515	FA5508	FA5746	FA5509	FA6350	FA5747	FA5749	FA5511	FA5512
Hd1	AAAAAAAAAAAAA	A01	Allele type	A	DEL	DEL	G	43bp	TT	DEL	A	AAG	AAAG	C	T	C
	BABAAAAAAAAAA	A02		G	DEL	36bp INS/-	G	43bp	TT	DEL	A	AAG	AAAG	C	T	C
	BABAAAAAAAABA	A03		G	DEL	36bp INS/-	G	43bp	TT	DEL	A	AAG	AAAG	C	C	C
	BABABAAAAAAAA	A04		G	DEL	36bp INS/-	G	43bp DEL	TT	DEL	A	AAG	AAAG	C	T	C
	BABAAABAAAAAA	A05		G	DEL	36bp INS/-	G	43bp	TT/-	1901bp INS	A	AAG	AAAG	C	T	C
	BABBAAAAAAABA	A06		G	DEL	36bp INS/-	1bp DEL	43bp	TT	DEL	A	AAG	AAAG	C	C	C
	BABAAAAAABABA	A07		G	DEL	36bp INS/-	G	43bp	TT	DEL	A	AAG	4bp DEL	C	C	C
	BABAAAABAAABA	A08		G	DEL	36bp INS/-	G	43bp	TT	DEL	C	AAG	AAAG	C	C	C
	BABAABABAAABA	A09		G	DEL	36bp INS/-	G	43bp	2bp DEL	DEL	C	AAG	AAAG	C	C	C
	BABAAAABAABBA	A10		G	DEL	36bp INS/-	G	43bp	TT	DEL	C	AAG	AAAG	T	C	C
	BABAAAABBAABA	A11		G	DEL	36bp INS/-	G	43bp	TT	DEL	C	3bp DEL	AAAG	C	C	C
	BABAAAABAAABB	A12		G	DEL	36bp INS/-	G	43bp	TT	DEL	C	AAG	AAAG	C	C	T
	BBBAAAABAAABA	A13		G	4424bp INS	36bp INS/-	G	43bp	TT	DEL	C	AAG	AAAG	C	C	C
	BABAABABAAABB	A14		G	DEL	36bp INS/-	G	43bp	2bp DEL	DEL	C	AAG	AAAG	C	C	T

Supplemental Fig. 4 (Continued)

Locus / Gene [18]	Allele	Allele No.	Chr.	6		
			IRGSP-1.0 position (bp)	23,855,144	23,855,920	23,861,465
			CDS position	Intron	Intron	5' region
			AA sub	-	-	-
			FA serial No.	54	55	56
			Assay name	FA5638	FA5639	FA5644
Se5	AAA	A01	Allele type	A	G	T
	BAA	A02		T	G	T
	ABA	A03		A	T	T
	AAB	A04		A	G	A

Locus / Gene [19]	Allele	Allele No.	Chr.	6
			IRGSP-1.0 position (bp)	24,557,073
			CDS position	901
			AA sub	L301V
			FA serial No.	57
			Assay name	FA5724
OsFTIP1	A	A01	Allele type	A
	B	A02		C

Locus / QTL_07	Allele	Allele No.	Chr.	7
			IRGSP-1.0 position (bp)	3,654,626
			CDS position	-
			AA sub	-
			FA serial No.	58
			Assay name	FA0419
**FA0419**	A	A01	Allele type	C
	B	A02		T

Supplemental Fig. 4 (Continued)

Locus	Allele	Allele No.	Chr.	7						
			IRGSP-1.0 position (bp)	9,150,835	9,152,479	9,152,655	9,154,664	9,155,047–9,155,048	9,156,042	9,156,348
			CDS position	3' region	697	521	157	5' region	5' region	5' region
			AA sub	-	P233A	D174V	E53Stop	-	-	-
			FA serial No.	59	60	61	62	63	64	65
Gene [20]			Assay name	FA5519	FA5525	FA5526	FA5530	FA6352	FA5533	FA5734
Ghd7	AAAAAAA	A01	Allele type	A	G	T	C	DEL	C	G
	AABAAAA	A02		A	G	A	C	DEL	C	G
	AAAAABA	A03		A	G	T	C	DEL	G	G
	ABBAAAA	A04		A	C	A	C	DEL	C	G
	BABAAAA	A05		G	G	A	C	DEL	C	G
	AAABABA	A06		A	G	T	A	DEL	G	G
	AAAABAB	A07		A	G	T	C	1901bp INS	C	A
	-------	A08		-	-	-	-	-	-	-

Locus	Allele	Allele No.	Chr.	7
			IRGSP-1.0 position (bp)	16,622,242
			CDS position	-
			AA sub	-
			FA serial No.	66
QTL_08			Assay name	FA0444
**FA0444**	A	A01	Allele type	A
	B	A02		G

Locus	Allele	Allele No.	Chr.	7	
			IRGSP-1.0 position (bp)	24,791,829	24,792,084
			CDS position	365	Intron
			AA sub	I122T	-
			FA serial No.	67	68
Gene [21]			Assay name	FA6353	FA6354
OsMADS18	AA	A01	Allele type	T	A
	AB	A02		T	C
	BB	A03		C	C

Supplemental Fig. 4 (Continued)

Locus / Gene [22]	Allele	Allele No.	Chr.	7							
			IRGSP-1.0 position (bp)	29,617,569	29,617,674	29,623,803	29,626,909	29,627,358–29,627,365	29,628,481	29,628,484	29,628,500
			CDS position	140	245	667	1,147	1,515–1,522	2,110	2,113	2,129
			AA sub	R47P	R82FS	D223N	A383T	K505FS	Y704H	Q705Stop	L710P
			FA serial No.	69	70	71	72	73	74	75	76
			Assay name	FA5437	FA5778	FA5433	FA5441	FA5779	FA5780	FA5781	FA5436
PRR37	AAAAAAAA	A01	Allele type	G	G	G	G	GAACGTTG	T	C	T
	BAAAAAAA	A02		C	G	G	G	GAACGTTG	T	C	T
	BABAAAAA	A03		C	G	A	G	GAACGTTG	T	C	T
	BABAAAAB	A04		C	G	A	G	GAACGTTG	T	C	C
	BAABAAAA	A05		C	G	G	A	GAACGTTG	T	C	T
	BAABBAAA	A06		C	G	G	A	8bp DEL	T	C	T
	BAABABAA	A07		C	G	G	A	GAACGTTG	C	C	T
	BAABAABA	A08		C	G	G	A	GAACGTTG	T	T	T
	BBABAAAA	A09		C	1bp DEL	G	A	GAACGTTG	T	C	T

Locus / Gene [23]	Allele	Allele No.	Chr.	8
			IRGSP-1.0 position (bp)	274,129
			CDS position	1,146
			AA sub	E382D
			FA serial No.	77
			Assay name	FA5562
Ehd3	A	A01	Allele type	T
	B	A02		G

Locus / Gene [24]	Allele	Allele No.	Chr.	8		
			IRGSP-1.0 position (bp)	2,381,725	2,387,949	2,388,554
			CDS position	3' region	1,278	673
			AA sub	-	Silent	T225A
			FA serial No.	78	79	80
			Assay name	FA5401	FA5404	FA5400
Hd18	AAA	A01	Allele type	C	T	T
	AAB	A02		C	T	C
	ABB	A03		C	C	C
	BAB	A04		T	T	C
	-BB	A05		-	C	C

Supplemental Fig. 4 (Continued)

Locus<br>Gene [25]	Allele	Allele No.	Chr.	8
			IRGSP-1.0 position (bp)	3,372,323
			CDS position	1,457
			AA sub	F486S
			FA serial No.	81
			Assay name	FA5725
OsLHY	A	A01	Allele type	T
	B	A02		C

Locus<br>Gene [26]	Allele	Allele No.	Chr.	8						InDel marker genotypes<br>8	
			IRGSP-1.0 position (bp)	4,332,710	4,333,856	4,333,870–4,333,871	4,334,417	4,335,990	4,359,634–4,359,635	4,332,835–4,333,950	4,334,627–4,334,645
			CDS position	3' region	884	869–870	323	5' region	5' region	790	95–113
			AA sub	-	N295S	F290Stop	K108FS	-	-	G264FS	A32FS
			FA serial No.	82	83	84	85	86	87	-	-
			Assay name	FA5449	FA5450 (Alternative to 1116-bp del.)	FA6349	FA5777	FA6355	FA5453 (Alternative to 19-bp del.)	Hd5_1116bpDel	Hd5_19bpDel_1
DTH8	AAAAAA	A01	Allele type	G	T	AA/-	T/-	A	DEL	1116bp	19bp
	AAAAAB	A02		G	T	AA/-	T/-	A	AT	1116bp	19bp DEL
	ABAABA	A03		G	C	AA/-	T/-	C	DEL	1116bp	19bp
	A-AABA	A04		G	-	AA/-	T/-	C	DEL	1116bp DEL	19bp
	-BAABA	A05		-	C	AA/-	T/-	C	DEL	1116bp	19bp
	-BBAAA	A06		-	C	TC	T/-	A	DEL	1116bp	19bp
	BBAABA	A07		A	C	AA/-	T/-	C	DEL	1116bp	19bp
	ABABBA	A08		G	C	AA/-	1bp DEL	C	DEL	1116bp	19bp
	BBAAB-	A09		A	C	AA/-	T/-	C	-	1116bp	19bp

Locus<br>QTL_09	Allele	Allele No.	Chr.	8	
			IRGSP-1.0 position (bp)	12,331,561	14,786,388
			CDS position	-	-
			AA sub	-	-
			FA serial No.	88	89
			Assay name	FA2167	FA0494
**FA2167-FA0494**	AA	A01	Allele type	C	T
	AB	A02		C	C
	BB	A03		T	C

Supplemental Fig. 4 (Continued)

Locus / Gene [27]	Allele	Allele No.	Chr.	10
			IRGSP-1.0 position (bp)	14,739,998
			CDS position	1,327
			AA sub	A443T
			FA serial No.	90
			Assay name	FA5569
Ehd2	A	A01	Allele type	C
	B	A02		T

Locus / Gene [28]	Allele	Allele No.	Chr.	10	
			IRGSP-1.0 position (bp)	17,077,589	17,078,199
			CDS position	655	-
			AA sub	G219R	-
			FA serial No.	91	92
			Assay name	FA5455	FA5458
Ehd1	AA	A01	Allele type	C	A
	AB	A02		C	G
	BA	A03		T	A

Locus / QTL_10	Allele	Allele No.	Chr.	10
			IRGSP-1.0 position (bp)	22,389,675
			CDS position	-
			AA sub	-
			FA serial No.	93
			Assay name	FA0603
**FA0603**	A	A01	Allele type	G
	B	A02		T

Supplemental Fig. 4 (Continued)

Locus / Gene [29]	Allele	Allele No.	Chr.	11
			IRGSP-1.0 position (bp)	4,432,316
			CDS position	3' region
			AA sub	-
			FA serial No.	94
			Assay name	FA5425
LOC_Os11g08410	A	A01	Allele type	A
	B	A02		G

Locus / QTL_11	Allele	Allele No.	Chr.	12
			IRGSP-1.0 position (bp)	4,000,543
			CDS position	-
			AA sub	-
			FA serial No.	95
			Assay name	FA1674
**FA1674**	A	A01	Allele type	C
	B	A02		G
	-	A03		-

Locus / QTL_12	Allele	Allele No.	Chr.	12
			IRGSP-1.0 position (bp)	24,387,429
			CDS position	-
			AA sub	-
			FA serial No.	96
			Assay name	FA1759
**FA1759**	A	A01	Allele type	G
	B	A02		T

Supplemental Fig. 4 (Continued)
