# Supplementary material for: Development of SNP genotyping assays for heading date in rice
Source: Breed Sci. 2024 Jun 25;74(3):274–84. doi: 10.1270/jsbbs.23093 (PMC11561416; doi:10.1270/jsbbs.23093)
Supplement: Supplementary file 2 — Supplemental Tables [file 74_274-s2.pdf]

Kitazawa et al./Breeding Science
doi: 10.1270/jsbbs.23093

Supplemental Table 1. List of the 377 cultivars used in this study and their alleles at 41 loci obtained in 96 assays

Cultivar No.	Cultivar name (Kanji and Kana in Japanese)	Cultivar name (listed alphabetically)	Origin(Area)	WRC/JRC number[a]	Cultivars used in assay for primary screening	Locus name	OsGI	FA0057	LOC_Os01g62780	FA0118	OsMADS51	OsVIL2	FA2420	OsCOL4	DTH2	Ehd4	Ef-cd	OsMADS50	FA0192	FA0193	DTH3b	Hd6	Hd16	FA1067	OsHDT1	Hd17	RFT1	Hd3a	Hd1	Se5	OsFTIP1	FA0419	Ghd7	FA0444	OsMADS18	PRR37	Ehd3	Hd18	OsLHY	DTH8	FA2167-FA0494	Ehd2	Ehd1	FA0603	LOC_Os11g08410	FA1674	FA1759
						Alleles at 41 loci																																									
						No.[b]	1	2	3	4	5	6	7	8	9	10	11	12	13	14	15	16	17	18	19	20	21	22	23	24	25	26	27	28	29	30	31	32	33	34	35	36	37	38	39	40	41
						No.[c]	[1]	-	[2]	-	[3]	[4]	-	[5]	[6]	[7]	[8]	[9]	-	-	[10]	[11]	[12]	-	[13]	[14]	[15]	[16]	[17]	[18]	[19]	-	[20]	-	[21]	[22]	[23]	[24]	[25]	[26]	-	[27]	[28]	-	[29]	-	-
						No.[d]	-	1	-	2	-	-	3	-	-	-	-	-	4	5	-	-	-	6	-	-	-	-	-	-	-	7	-	8	-	-	-	-	-	-	9	-	-	10	-	11	12
1	愛知旭	Aichiasahi	Japan(Aichi)	JRC 52	✓		1	1	2	2	4	2	1	2	1	1	1	1	1	1	1	2	1	1	1	1	1	1	2	1	1	1	1	1	2	1	1	2	1	1	1	1	1	1	1	1	1
2	あいちのかおりSBL	Aichinokaori SBL	Japan(Aichi)	-	✓		1	2	1	2	4	1	1	1	1	1	1	1	1	1	1	1	1	1	1	2	1	1	2	1	1	2	3	2	2	1	1	2	1	1	1	1	1	2	1	1	1
3	愛国	Aikoku	Japan(Fukui)	JRC 26	✓		3	1	1	2	1	1	1	1	1	1	1	1	1	1	1	4	1	2	1	2	1	1	2	1	1	2	7	2	2	1	1	2	2	1	3	1	1	2	1	1	1
4	赤毛	Akage	Japan(Akita)	JRC 17	✓		1	1	1	2	4	1	1	2	1	1	1	4	1	1	1	2	1	1	2	2	1	3	12	1	2	2	6	1	2	4	2	1	1	1	3	1	1	1	1	1	1
5	赤米（長崎）	Akamai(Nagasaki)	Japan(Nagasaki)	JRC 40			1	1	2	1	1	2	2	1	2	1	1	1	1	1	1	2	1	1	2	2	1	1	2	1	2	1	3	1	2	1	1	2	2	1	1	1	1	1	1	1	1
6	赤米（徳島）	Akamai(Tokushima)	Japan(Tokushima)	JRC 41			4	1	4	2	2	1	2	2	2	1	1	4	1	1	1	2	1	2	2	2	1	1	8	1	1	2	3	1	2	7	1	2	1	3	1	1	1	1	1	1	1
7	あかね空	Akanezora	Japan(Aichi)	-	✓		1	2	1	2	1	1	1	1	1	1	1	4	2	1	1	1	3	1	1	2	1	1	1	1	1	1	1	2	2	1	1	1	1	1	3	1	1	2	1	2	1
8	アケボノ	Akebono	Japan(Mie)	-			1	1	2	1	4	2	1	1	1	1	1	1	1	1	1	2	1	1	1	1	1	1	2	1	1	1	1	2	1	1	1	2	1	1	1	1	1	2	1	1	1
9	あ系883	Akei 883	Japan(Aichi)	-	✓		1	2	1	1	4	1	1	1	1	1	1	1	1	1	1	1	1	1	1	1	1	1	1	1	1	1	1	2	2	1	1	1	1	1	1	1	1	2	1	1	2
10	あきあかね	Akiakane	Japan(Niigata)	-			3	2	1	2	1	1	1	2	1	1	1	4	2	2	1	1	3	1	1	2	1	1	1	1	1	1	1	1	2	1	1	2	1	1	1	1	1	2	1	1	2
11	秋晴	Akibare	Japan(Aichi)	-	✓		1	1	1	1	4	1	1	1	1	1	1	1	1	1	1	1	1	1	1	1	1	1	1	1	1	1	3	2	2	1	1	2	1	1	3	1	1	2	1	2	1
12	アキチカラ	Akichikara	Japan(Niigata)	-			3	2	1	1	4	1	1	1	1	1	1	2	1	1	1	2	1	1	1	2	1	1	4	1	1	2	1	1	1	2	1	4	1	1	3	2	1	1	1	2	2
13	あきだわら	Akidawara	Japan(Ibaraki)	-			2	2	1	2	1	1	1	1	1	1	1	4	2	2	1	1	1	2	1	1	1	1	1	1	1	1	1	2	2	1	1	1	1	1	3	1	1	2	1	1	2
14	秋はるか	Akiharuka	Japan(Fukuoka)	-			1	1	1	2	1	1	1	1	1	1	1	4	2	2	1	1	1	2	1	2	1	1	1	1	1	2	3	1	2	1	1	2	1	1	1	1	1	2	1	2	2
15	アキヒカリ	Akihikari	Japan(Aomori)	-	✓		3	2	1	1	4	1	1	2	1	1	1	2	1	1	1	2	1	1	2	1	1	1	4	1	1	2	1	1	1	2	1	4	1	1	3	2	1	1	1	2	2
16	あきほ	Akiho	Japan(Hokkaido)	-	✓		3	1	1	2	4	1	2	2	1	1	1	2	1	1	1	1	1	1	2	1	1	3	2	1	1	2	6	1	1	4	1	1	1	1	3	2	1	2	1	1	1
17	あきまつり	Akimatsuri	Japan(Yamaguchi)	-			1	2	1	1	1	1	1	1	1	1	1	4	2	2	1	1	1	2	1	1	1	1	1	1	1	1	3	2	2	1	1	1	1	1	3	1	1	2	1	1	2
18	あきさかり	Akisakari	Japan(Fukui)	-			1	2	1	2	1	1	1	1	1	1	1	4	2	2	1	1	3	2	1	2	1	1	1	1	1	1	1	1	2	1	1	2	1	1	1	2	1	1	1	2	1
19	あきさやか	Akisayaka	Japan(Fukuoka)	-			3	2	1	2	1	1	1	2	1	1	1	4	2	2	1	4	1	2	1	2	1	1	1	1	1	2	1	1	1	1	1	1	1	1	3	1	1	2	1	2	2
20	秋田106号	Akita 106	Japan(Akita)	-	✓		3	2	1	2	1	1	1	1	1	1	1	4	2	2	1	1	1	1	1	2	1	4	4	1	1	2	1	1	2	1	1	2	1	1	3	1	1	2	1	2	1
21	秋田63号	Akita 63	Japan(Akita)	-			3	2	1	2	1	1	1	2	1	1	1	4	2	2	1	1	1	1	1	2	1	1	4	1	1	2	3	1	1	2	1	1	1	1	1	1	1	1	1	2	1
22	あきたこまち	Akitakomachi	Japan(Akita)	-	✓		3	2	1	2	1	1	1	1	1	1	1	1	1	2	1	1	1	2	1	2	1	1	4	1	1	2	1	1	2	2	1	1	1	1	3	2	1	1	1	2	2
23	アクネモチ	Akunemochi	Japan(Kagoshima)	-			1	1	2	1	4	2	2	2	1	1	1	1	1	1	1	2	1	1	1	2	1	1	2	1	1	1	1	1	1	2	1	4	2	1	1	1	1	1	1	1	1
24	あみちゃんまい	Amichammai	Japan(Niigata)	-			3	2	1	1	4	1	2	2	2	1	1	4	2	2	1	1	3	1	1	2	1	1	4	1	1	2	1	1	1	1	1	1	1	1	3	1	1	1	1	2	1
25	葵の風	Aoinokaze	Japan(Aichi)	-	✓		1	2	1	2	1	1	1	1	1	1	1	4	2	2	1	1	1	2	1	2	1	1	1	1	1	1	1	2	2	1	1	1	1	1	3	1	1	2	1	1	2
26	青系IL2号	Aokei IL 2	Japan(Aomori)	-	✓		3	2	1	1	1	1	2	2	1	1	1	1	1	2	1	1	1	1	1	2	1	1	4	1	1	2	1	1	1	2	1	1	1	1	3	1	1	1	1	2	2
27	朝日	Asahi	Japan(Unknown)	-	✓		1	1	2	1	4	2	2	2	1	1	1	1	1	1	1	2	1	1	1	1	1	1	2	1	1	1	1	1	1	2	1	2	1	1	1	1	1	2	1	1	1
28	あさひの夢	Asahinoyume	Japan(Aichi)	-	✓		1	1	1	2	1	1	1	1	1	1	1	1	1	1	1	1	1	1	1	1	1	1	2	1	1	1	3	2	2	1	1	1	1	1	1	1	1	2	1	1	1
29	朝の光	Asanohikari	Japan(Aichi)	-	✓		1	1	1	1	1	1	1	1	1	1	1	1	1	1	1	1	1	1	1	1	1	4	1	1	1	1	1	1	2	2	1	1	1	1	1	1	1	2	1	1	1
30	あさゆき	Asayuki	Japan(Aomori)	-			1	1	1	2	1	1	1	1	1	1	1	2	2	2	1	1	1	1	1	2	1	1	4	1	1	2	1	1	2	1	1	2	1	1	3	1	1	2	1	2	2
31	あそみのり	Asominori	Japan(Fukuoka)	-			1	1	2	1	4	1	1	1	1	1	1	1	1	1	1	1	1	1	1	2	1	1	1	1	1	1	1	1	2	2	1	4	1	1	1	1	1	2	1	1	1
32	阿波赤米	Awaakamai	Japan(Tokushima)	-			1	1	1	2	4	1	2	2	1	1	1	2	1	1	1	2	1	2	2	1	1	1	2	1	1	2	1	1	1	2	1	2	2	1	1	1	1	1	1	1	1
33	Basilanon	Basilanon	Philippines	WRC 44	✓		4	1	4	2	2	1	2	2	2	1	1	2	1	1	1	3	2	1	2	2	1	1	6	2	2	2	1	1	3	2	1	2	2	3	1	1	2	1	1	2	1
34	Bei Khe	Bei Khe	Cambodia	WRC 03	✓		4	1	3	2	2	2	2	2	3	2	1	3	1	1	2	3	2	1	2	3	2	2	3	4	2	2	4	1	3	7	1	3	2	4	1	1	2	1	1	1	1
35	べこあおば	Bekoaoba	Japan(Akita)	-			3	2	1	1	4	1	1	1	1	1	1	4	2	2	1	2	1	1	2	1	1	4	5	3	1	2	3	1	1	2	1	1	1	1	3	1	1	1	1	1	1
36	べこげんき	Bekogenki	Japan(Akita)	-			1	1	1	2	1	1	1	1	1	1	1	2	1	1	1	2	1	1	1	2	1	1	4	1	1	2	3	2	2	2	1	2	1	1	3	2	2	2	1	1	2
37	べこごのみ	Bekogonomi	Japan(Akita)	-			1	1	1	1	4	1	1	1	1	1	1	4	1	1	1	1	1	1	2	1	1	4	4	1	1	2	3	2	1	3	1	1	1	1	3	2	1	1	1	1	1
38	ベニセンゴク	Benisengoku	Japan(Fukuoka)	-			1	1	2	1	4	2	2	2	1	1	1	1	1	1	1	2	1	1	1	1	1	1	2	1	1	1	1	2	2	2	1	4	1	1	1	1	1	1	1	1	1
39	Bleiyo	Bleiyo	Thailand	WRC 63	✓		4	1	3	2	3	2	2	2	3	2	1	3	1	1	2	3	2	1	2	3	2	2	3	4	2	2	4	1	3	5	1	3	2	5	1	1	2	1	1	1	1
40	坊主	Bouzu	Japan(Hokkaido)	-	✓		1	1	1	2	4	1	2	2	1	1	1	4	1	1	1	2	1	1	1	2	1	3	12	1	2	2	6	1	2	4	2	1	1	1	3	1	1	1	1	1	1
41	坊主6号	Bouzu 6	Japan(Hokkaido)	-	✓		1	2	1	2	4	1	2	2	1	1	1	4	1	1	1	2	1	1	2	2	1	3	12	1	2	2	6	1	2	4	2	1	1	2	3	1	1	1	1	1	1
42	坊主糯	Bouzumochi	Japan(Oita)	JRC 13			1	1	2	2	4	1	2	1	1	1	1	2	1	1	1	2	1	1	2	2	1	1	11	2	1	2	1	1	2	2	2	4	1	1	1	1	1	2	1	2	1
43	ちほみのり	Chihominori	Japan(Akita)	-			3	2	1	1	1	1	1	1	1	1	1	2	1	1	1	1	3	1	1	2	1	1	4	1	1	1	1	1	1	1	1	2	1	1	3	1	1	2	1	2	1
44	ちくし52号	Chikushi 52	Japan(Fukuoka)	-			3	2	1	2	1	1	1	1	1	1	1	2	1	1	1	1	3	2	2	2	1	1	1	1	1	2	1	1	2	1	1	2	1	1	3	2	1	2	1	2	2
45	ちくし89号	Chikushi 89	Japan(Fukuoka)	-	✓		3	2	1	2	1	1	1	2	1	1	1	4	2	2	1	1	1	2	1	2	1	1	1	1	1	2	1	1	2	1	1	2	1	1	1	1	1	1	1	2	2
46	ちくし粉85号	Chikushi Kona 85	Japan(Fukuoka)	-			3	1	1	1	1	1	2	2	1	1	1	1	1	1	1	2	1	1	1	1	1	1	1	1	1	1	4	1	3	1	1	4	1	1	1	1	1	2	1	2	1
47	チヨホナミ	Chiyohonami	Japan(Miyagi)	-	✓		1	1	1	1	1	1	1	1	1	1	1	4	2	2	1	1	1	1	1	2	1	1	4	1	1	1	1	1	1	3	1	2	1	1	3	1	1	2	1	2	1
48	チヨニシキ	Chiyonishiki	Japan(Aichi)	-	✓		3	2	1	2	1	1	1	1	1	1	1	4	2	2	1	1	1	1	1	2	1	1	4	1	1	2	1	1	2	1	1	2	1	1	3	1	1	1	1	2	1
49	中稜2号	Chujo 2	Japan(Aichi)	-	✓		1	1	2	1	4	2	2	2	1	1	1	1	1	1	1	2	1	1	1	1	1	1	2	1	1	1	3	2	2	1	1	4	1	1	1	1	1	2	1	2	1
50	中京旭	Chukyoasahi	Japan(Aichi)	-	✓		1	1	2	1	4	2	2	2	1	1	1	1	1	1	1	2	1	1	1	1	1	1	2	1	1	1	1	1	1	1	1	2	1	1	1	1	1	2	1	1	1

(Continued)

Cultivar No.	Cultivar name (Kanji and Kana in Japanese)	Cultivar name (listed alphabetically)	Origin(Area)	WRC/JRC number[a]	Cultivars used in assay for primary screening	Alleles at 41 loci																																								
					No.[d]	-	1	-	2	-	-	3	-	-	-	-	-	4	5	-	-	-	6	-	-	-	-	-	-	-	7	-	8	-	-	-	-	-	-	9	-	-	10	-	11	12
					No.[c]	[1]	-	[2]	-	[3]	[4]	-	[5]	[6]	[7]	[8]	[9]	-	-	[10]	[11]	[12]	-	[13]	[14]	[15]	[16]	[17]	[18]	[19]	-	[20]	-	[21]	[22]	[23]	[24]	[25]	[26]	-	[27]	[28]	-	[29]	-	-
					No.[b]	1	2	3	4	5	6	7	8	9	10	11	12	13	14	15	16	17	18	19	20	21	22	23	24	25	26	27	28	29	30	31	32	33	34	35	36	37	38	39	40	41
					Locus name	OsGI	FA0057	LOC_Os01g62780	FA0118	OsMADS51	OsVIL2	FA2420	OsCOL4	DTH2	Ehd4	Ef-cd	OsMADS50	FA0192	FA0193	DTH3b	Hd6	Hd16	FA1067	OsHDT1	Hd17	RFT1	Hd3a	Hd1	Se5	OsFTIP1	FA0419	Ghd7	FA0444	OsMADS18	PRR37	Ehd3	Hd18	OsLHY	DTH8	FA2167-FA0494	Ehd2	Ehd1	FA0603	LOC_Os11g08410	FA1674	FA1759
51	大地の風	Daichinokaze	Japan(Aichi)	-	✓	1	2	1	2	1	1	1	1	1	1	1	4	2	1	1	1	1	2	1	2	1	1	2	1	1	1	3	2	2	1	1	1	1	1	1	1	1	2	1	1	2
52	団子	Dango	Japan(Unknown)	JRC 25		1	1	2	2	4	1	1	2	1	1	1	1	1	1	1	2	1	1	2	1	1	1	2	1	1	1	1	2	1	1	1	4	1	1	1	1	1	2	1	1	1
53	Davao 1	Davao 1	Philippines	WRC 07	✓	4	1	4	2	2	2	2	2	3	2	1	3	1	1	2	3	2	1	2	3	2	5	7	3	2	2	4	1	3	6	1	3	2	8	1	1	2	1	1	1	1
54	Deng Pao Zhai	Deng Pao Zhai	China	WRC 19	✓	4	1	3	2	2	1	2	2	3	2	1	3	1	1	2	3	2	1	2	3	1	2	3	4	2	2	2	1	3	5	1	3	2	4	1	1	2	1	1	1	1
55	どまんなか	Domannaka	Japan(Yamagata)	-	✓	3	2	1	2	1	1	1	1	1	1	1	1	2	1	1	1	3	1	1	2	1	4	4	1	1	2	3	2	2	1	1	2	1	1	1	1	1	2	1	2	2
56	どんとこい	Dontokoi	Japan(Niigata)	-	✓	3	2	1	2	1	1	1	2	1	1	1	4	2	2	1	1	3	2	1	2	1	1	1	1	1	1	1	1	1	1	1	1	1	1	1	1	1	1	1	2	2
57	えみのあき	Eminoaki	Japan(Akita)	-		1	1	1	1	1	1	1	1	1	1	1	4	2	1	1	1	1	1	1	2	1	1	4	1	1	2	1	1	2	3	1	2	1	1	1	1	1	2	1	2	1
58	笑みの絆	Eminokizuna	Japan(Niigata)	-		3	1	1	2	1	1	1	2	1	1	1	1	1	1	1	1	3	2	1	2	1	1	1	1	1	1	1	2	2	2	1	2	1	1	1	1	1	1	1	1	2
59	えみゆたか	Emiyutaka	Japan(Aomori)	-		3	2	1	1	4	1	1	1	1	1	1	2	1	1	1	1	1	1	2	2	1	1	4	1	1	2	1	1	1	1	1	1	1	1	3	2	1	2	1	1	2
60	フジヒカリ	Fujihikari	Japan(Hiroshima)	-	✓	1	2	1	2	1	1	1	1	1	1	1	2	2	2	1	1	3	2	1	1	1	1	9	1	1	2	1	1	2	1	1	1	1	1	3	1	1	2	1	2	2
61	フジミノリ	Fujiminori	Japan(Aomori)	-	✓	1	1	1	1	4	1	2	2	1	1	1	2	1	1	1	2	1	1	2	1	1	1	4	1	1	2	1	1	1	2	1	4	1	1	3	2	1	1	1	2	2
62	藤坂5号	Fujisaka 5	Japan(Aomori)	-	✓	1	1	1	1	4	2	2	2	1	1	1	2	1	1	1	2	1	1	2	1	1	4	4	1	1	1	3	2	2	3	1	2	1	1	1	2	1	1	1	2	2
63	ふ系175号	Fukei 175	Japan(Aomori)	-	✓	3	2	1	2	1	1	2	2	1	1	1	2	1	1	1	1	1	1	1	2	1	1	4	1	1	2	1	1	2	1	1	2	1	1	3	1	1	2	1	2	2
64	富国	Fukoku	Japan(Hokkaido)	JRC 46		3	1	1	2	4	1	1	1	1	1	1	4	1	1	1	4	1	2	2	2	1	3	12	1	1	2	7	2	2	4	2	1	1	2	3	1	1	2	1	1	1
65	ふくひびき	Fukuhibiki	Japan(Akita)	-	✓	1	1	1	1	4	1	1	1	1	1	1	4	2	2	1	1	1	1	2	1	1	4	4	1	1	2	3	2	2	3	1	1	1	1	1	1	1	2	1	2	1
66	ふくいずみ	Fukuizumi	Japan(Fukuoka)	-		3	2	1	2	1	1	1	1	1	1	1	4	2	2	1	1	1	2	1	2	1	1	1	1	1	1	3	2	1	1	1	1	1	1	1	1	1	2	1	1	1
67	ふくまる	Fukumaru	Japan(Ibaraki)	-	✓	1	1	1	2	1	1	1	1	1	1	1	4	2	1	1	1	3	1	1	2	1	1	4	1	1	2	1	1	2	1	1	2	1	1	3	1	1	1	1	1	1
68	フクニシキ	Fukunishiki	Japan(Akita)	-		1	1	1	1	4	1	1	2	1	1	1	1	2	2	1	1	1	1	1	2	1	4	9	2	2	1	1	1	1	2	1	1	1	1	1	1	1	2	1	1	1
69	ふくのこ	Fukunoko	Japan(Hiroshima)	-		1	2	1	2	4	2	2	1	2	1	1	1	1	1	1	1	1	1	1	2	1	1	2	1	1	1	1	1	1	2	1	1	1	1	3	1	1	1	1	2	1
70	ふさおとめ	Fusaotome	Japan(Chiba)	-	✓	1	1	1	2	1	1	1	1	1	1	1	4	2	1	1	1	3	1	1	2	1	4	4	1	1	2	1	1	2	1	1	2	1	1	3	1	1	2	1	1	2
71	双葉	Futaba	Japan(Aichi)	-	✓	1	1	1	1	4	2	2	2	1	1	1	2	1	1	1	2	1	1	1	1	1	1	2	1	1	1	1	1	1	3	1	2	1	1	1	2	1	1	1	2	1
72	凱旋糯	Gaisenmochi	Japan(Unknown)	JRC 01	✓	4	1	2	2	4	1	2	2	2	1	1	4	1	1	1	2	1	1	2	2	1	1	1	1	1	2	1	1	2	2	2	4	1	1	1	1	1	1	1	2	1
73	げんきまる	Genkimaru	Japan(Miyagi)	-		1	1	1	2	1	1	1	1	1	1	1	4	2	2	1	1	1	1	1	2	1	1	4	1	1	2	1	1	2	1	1	2	1	1	3	1	1	2	1	2	1
74	元気つくし	Genkitsukushi	Japan(Fukuoka)	-	✓	3	2	1	2	1	1	1	1	1	1	1	4	2	2	1	1	3	2	1	2	1	1	1	1	1	1	1	1	2	1	1	2	1	1	1	1	1	1	1	2	1
75	銀坊主	Ginbouzu	Japan(Ishikawa)	JRC 27	✓	3	1	1	1	1	1	1	1	1	1	1	1	1	1	1	1	1	2	1	2	1	1	2	1	2	1	1	2	2	1	1	1	1	1	3	1	1	2	1	1	1
76	吟のさと	Ginnosato	Japan(Fukuoka)	-		1	1	2	1	1	1	1	2	1	1	1	2	1	1	1	2	1	1	1	1	1	4	2	1	1	1	1	1	1	1	1	4	1	1	3	1	1	1	2	1	1
77	吟おうみ	Ginomi	Japan(Shiga)	-		1	1	2	1	1	1	1	1	1	1	1	1	1	1	1	1	1	1	1	1	1	1	2	1	1	1	1	1	2	1	1	1	1	1	1	1	1	2	1	1	1
78	五百万石	Gohyakumangoku	Japan(Niigata)	-	✓	1	1	1	1	4	2	2	2	1	1	1	1	1	1	1	2	1	1	2	1	1	1	4	1	1	1	1	2	1	2	1	2	1	1	1	2	1	1	1	1	1
79	ゴロピカリ	Goropikari	Japan(Gumma)	-	✓	1	1	1	2	1	2	1	1	1	1	1	1	1	1	1	1	1	1	1	1	1	1	1	1	1	1	1	2	2	1	1	2	1	1	3	1	1	1	1	1	1
80	桂朝2号	Guizhao 2	China	-		5	1	3	2	3	1	2	2	3	2	1	3	1	1	2	3	1	1	2	3	1	1	7	3	2	2	2	1	3	5	1	3	2	4	1	1	2	1	1	1	1
81	ハバタキ	Habataki	Japan(Niigata)	-	✓	5	1	3	2	3	1	2	2	3	1	1	1	1	1	2	3	2	1	2	1	1	1	7	3	2	2	4	1	3	5	1	3	2	8	1	1	2	1	1	1	1
82	はえぬき	Haenuki	Japan(Yamagata)	-	✓	3	2	1	1	4	1	1	1	1	1	1	1	1	1	1	1	1	2	1	2	1	1	4	1	1	1	1	1	2	3	1	2	1	1	3	2	1	2	1	2	2
83	はいごころ	Haigokoro	Japan(Hiroshima)	-		1	1	1	1	1	1	1	1	1	1	1	4	2	2	1	1	1	2	1	2	1	1	2	1	1	2	1	1	2	1	1	1	1	1	3	1	1	1	1	1	2
84	ハマアサヒ	Hamaasahi	Japan(Aomori)	-		1	1	1	2	4	2	2	2	1	1	1	2	1	1	1	1	1	1	2	1	1	3	12	1	1	2	7	2	1	4	1	4	1	1	3	2	1	1	1	1	2
85	はまさり	Hamasari	Japan(Saitama)	-		2	1	1	1	4	1	2	2	2	1	1	1	1	1	1	1	1	1	2	3	1	1	2	1	1	2	1	1	1	1	1	1	1	1	2	1	3	2	1	1	2
86	ハナエチゼン	Hanaechizen	Japan(Fukui)	-	✓	1	1	1	1	1	1	1	1	1	1	1	4	2	2	1	1	3	1	1	2	1	4	9	2	2	1	1	1	2	1	1	1	1	1	3	1	1	2	1	1	1
87	はなの舞	Hananomai	Japan(Yamagata)	-	✓	3	2	1	2	4	1	1	1	1	1	1	2	1	1	1	1	3	1	1	2	1	1	4	1	1	2	1	1	2	1	1	2	1	1	3	1	1	1	1	2	1
88	はるもに	Harumoni	Japan(Fukuoka)	-		1	1	1	2	1	1	1	1	1	1	1	4	2	2	1	1	1	1	1	2	1	1	1	1	1	2	3	1	2	1	1	2	1	1	3	1	1	1	1	2	2
89	晴るる	Haruru	Japan(Yamaguchi)	-	✓	3	2	1	1	1	1	1	1	1	1	1	4	2	1	1	1	3	1	1	2	1	1	1	1	1	2	1	1	2	1	1	4	1	1	3	1	1	1	1	1	2
90	走坊主	Hashiribouzu	Japan(Hokkaido)	-	✓	1	1	1	2	4	1	2	2	1	1	1	4	1	1	1	2	1	1	2	2	1	3	12	1	2	2	6	1	2	4	2	1	1	1	3	1	1	1	1	1	1
91	八束穂	Hassokuho	Japan(Unknown)	JRC 18		1	1	1	1	1	1	2	2	1	1	1	4	1	1	1	2	1	1	2	1	1	1	9	1	2	2	1	1	2	2	1	4	1	1	1	1	2	1	2	1	1
92	初星	Hatsuboshi	Japan(Aichi)	-	✓	1	2	1	2	1	2	1	1	1	1	1	4	2	1	1	1	1	1	1	1	1	4	4	1	1	2	1	1	2	1	1	2	1	1	3	1	1	1	1	2	2
93	ハツニシキ	Hatsunishiki	Japan(Akita)	-	✓	1	2	1	2	4	1	1	2	1	1	1	1	2	2	1	1	3	1	1	2	1	1	4	1	1	2	1	1	2	1	1	1	1	1	3	1	1	2	1	2	1
94	ハツシモ	Hatsushimo	Japan(Aichi)	-	✓	1	1	1	1	4	1	1	1	1	1	1	1	1	1	1	1	1	1	1	2	1	1	2	1	1	1	1	2	2	1	1	2	1	1	1	1	1	2	1	1	1
95	初雫	Hatsushizuku	Japan(Hokkaido)	-		3	2	1	2	1	1	2	2	1	1	1	4	1	2	1	1	1	1	2	1	1	3	2	1	1	2	6	2	2	4	1	2	1	1	1	2	1	2	1	2	1
96	はやまさり	Hayamasari	Japan(Hokkaido)	-	✓	3	1	1	2	4	1	2	2	2	1	1	2	1	1	1	1	1	2	2	1	1	3	2	1	1	2	6	2	1	4	1	4	1	2	3	1	1	1	1	1	2
97	ハヤニシキ	Hayanishiki	Japan(Akita)	-		3	1	1	1	4	2	2	2	1	1	1	2	1	1	1	1	3	1	2	2	1	4	4	1	1	2	3	2	2	3	1	4	1	1	3	2	1	1	1	2	2
98	ひだほまれ	Hidahomare	Japan(Gifu)	-		1	1	2	1	4	2	2	2	1	1	1	1	1	1	1	1	1	1	1	1	1	1	4	1	1	1	1	1	1	2	1	2	1	1	1	1	1	1	1	2	2
99	姫ごのみ	Himegonomi	Japan(Hiroshima)	-		1	1	1	2	1	1	1	1	1	1	1	4	2	2	1	1	1	2	1	2	1	1	1	1	1	2	1	1	2	1	1	1	1	1	3	1	1	2	1	2	2
100	ヒメノモチ	Himenomochi	Japan(Akita)	JRC 50	✓	1	2	1	1	1	2	1	2	1	1	1	1	1	1	1	1	3	1	2	2	1	4	4	1	1	1	3	1	2	1	1	2	2	1	1	2	1	1	1	2	2

(Continued)

Cultivar No.	Cultivar name (Kanji and Kana in Japanese)	Cultivar name (listed alphabetically)	Origin(Area)	WRC/JRC number[a]	Cultivars used in assay for primary screening		Alleles at 41 loci																																								
						No.[d]	-	1	-	2	-	-	3	-	-	-	-	-	4	5	-	-	-	6	-	-	-	-	-	-	-	7	-	8	-	-	-	-	-	-	9	-	-	10	-	11	12
						No.[c]	[1]	-	[2]	-	[3]	[4]	-	[5]	[6]	[7]	[8]	[9]	-	-	[10]	[11]	[12]	-	[13]	[14]	[15]	[16]	[17]	[18]	[19]	-	[20]	-	[21]	[22]	[23]	[24]	[25]	[26]	-	[27]	[28]	-	[29]	-	-
						No.[b]	1	2	3	4	5	6	7	8	9	10	11	12	13	14	15	16	17	18	19	20	21	22	23	24	25	26	27	28	29	30	31	32	33	34	35	36	37	38	39	40	41
						Locus name	OsGI	FA0057	LOC_Os01g62780	FA0118	OsMADS51	OsVIL2	FA2420	OsCOL4	DTH2	Ehd4	Ef-cd	OsMADS50	FA0192	FA0193	DTH3b	Hd6	Hd16	FA1067	OsHDT1	Hd17	RFT1	Hd3a	Hd1	Se5	OsFTIP1	FA0419	Ghd7	FA0444	OsMADS18	PRR37	Ehd3	Hd18	OsLHY	DTH8	FA2167-FA0494	Ehd2	Ehd1	FA0603	LOC_Os11g08410	FA1674	FA1759
101	日の出	Hinode	Japan(Kinki)	JRC 03			1	1	2	2	4	1	2	2	2	1	1	4	1	1	1	2	1	1	2	2	1	1	11	1	1	2	1	1	2	2	2	4	1	1	1	1	1	2	1	2	1
102	ヒノヒカリ	Hinohikari	Japan(Miyazaki)	-	✓		1	2	1	2	1	1	1	1	1	1	1	4	2	2	1	1	1	2	1	2	1	1	1	1	1	2	1	1	2	1	1	2	1	1	3	1	1	1	1	2	2
103	平山	Hirayama	Japan(Tokyo)	JRC 10	✓		1	1	2	2	4	1	2	2	2	1	1	4	1	1	1	2	1	1	2	2	1	1	11	1	1	2	1	1	2	2	1	4	1	1	1	1	2	1	1	2	1
104	ひとはな	Hitohana	Japan(Fukuoka)	-			1	1	1	1	1	1	1	1	1	1	1	1	1	1	1	1	1	1	1	1	1	4	2	1	1	1	1	1	1	1	1	1	1	1	1	1	1	1	1	2	1
105	ひとめぼれ	Hitomebore	Japan(Miyagi)	-	✓		3	2	1	2	1	1	1	1	1	1	1	4	2	1	1	1	1	1	1	2	1	1	4	1	1	2	1	1	2	1	1	2	1	1	3	1	1	2	1	2	2
106	冷立稲	Hiyadachito	Japan(Yamagata)	JRC 45			1	1	1	1	4	2	1	2	1	1	1	2	1	1	1	2	1	1	2	1	1	1	12	1	1	2	1	1	1	3	1	2	1	1	3	1	1	1	1	1	1
107	ヒヨクモチ	Hiyokumochi	Japan(Fukuoka)	-			1	1	2	1	4	1	2	2	1	1	1	1	1	1	1	2	1	1	1	1	1	4	2	1	1	1	1	1	1	1	1	4	1	1	3	1	1	1	1	1	1
108	ほほほの穂	Hohohonoho	Japan(Ishikawa)	-			1	1	1	1	1	1	1	2	1	1	1	1	1	2	1	1	1	2	1	2	1	4	9	2	2	1	1	1	1	2	1	1	1	1	3	1	1	2	1	2	1
109	北海188号	Hokkai 188	Japan(Hokkaido)	-			1	1	1	1	4	1	2	1	2	1	1	2	1	1	1	1	1	2	2	1	1	3	4	1	1	2	7	2	1	4	2	2	2	1	3	1	1	2	1	1	2
110	北海PL3	Hokkai PL 3	Japan(Hokkaido)	-			3	1	1	2	4	1	1	1	1	1	1	4	1	1	1	1	1	1	2	1	1	3	1	1	1	2	6	2	2	4	2	2	1	1	1	1	1	2	1	2	1
111	北海PL7	Hokkai PL 7	Japan(Hokkaido)	-			2	1	1	2	4	1	2	2	1	1	1	2	1	1	1	1	1	1	2	2	1	3	2	1	1	2	6	1	2	4	2	2	2	1	3	1	1	2	1	1	2
112	北海PL9	Hokkai PL 9	Japan(Hokkaido)	-			2	1	1	2	4	1	2	1	2	1	1	2	1	1	1	1	1	1	2	1	1	3	2	1	1	2	6	1	1	4	2	2	2	1	3	2	1	2	1	1	2
113	ほっかりん	Hokkarin	Japan(Aomori)	-	✓		1	1	1	1	1	1	1	2	1	1	1	2	2	2	1	1	1	2	2	1	1	3	4	1	1	2	1	1	2	1	1	1	1	1	3	1	1	2	1	2	2
114	北陸193号	Hokuriku 193	Japan(Niigata)	-			5	1	4	2	3	1	2	2	3	1	1	1	1	1	2	2	2	1	2	1	1	1	7	3	2	2	4	1	3	5	1	3	2	1	3	1	2	1	1	1	1
115	宝満神社米	Homanjinjamai	Japan(Kagoshima)	JRC 54			1	1	2	1	4	2	2	2	2	1	1	2	1	1	1	2	1	1	2	2	1	1	2	1	2	1	1	1	2	2	1	2	2	1	3	1	1	1	2	1	1
116	ホウネンワセ	Hounenwase	Japan(Fukui)	-	✓		3	2	1	2	4	1	1	2	1	1	1	1	1	1	1	1	3	1	1	2	1	1	4	1	1	2	1	1	2	1	1	1	1	1	3	1	1	2	1	2	1
117	ホシアオバ	Hoshiaoba	Japan(Hiroshima)	-			5	1	1	2	1	1	2	1	1	1	1	4	2	2	1	2	1	1	1	2	1	1	4	1	1	2	3	1	1	2	1	1	1	1	1	1	1	2	1	1	1
118	ほしじるし	Hoshijirushi	Japan(Ibaraki)	-			1	1	1	2	1	1	1	1	1	1	1	4	2	2	1	1	3	2	1	2	1	1	1	1	1	1	1	1	2	1	1	2	1	1	1	1	1	2	1	2	2
119	ほしみのり	Hoshiminori	Japan(Ibaraki)	-			1	1	1	1	1	1	1	1	1	1	1	4	2	2	1	1	3	2	1	2	1	4	1	1	1	1	1	2	2	2	1	1	1	1	1	2	1	1	1	2	2
120	ホシニシキ	Hoshinishiki	Japan(Ibaraki)	-			2	1	1	1	4	1	1	1	1	1	1	1	1	1	1	1	1	1	2	2	1	4	1	1	1	1	1	1	1	1	1	1	1	1	1	1	1	1	1	1	1
121	ほしのゆめ	Hoshinoyume	Japan(Hokkaido)	-	✓		1	1	1	2	1	1	2	2	1	1	1	1	2	1	1	1	1	1	2	1	1	3	2	1	1	2	6	1	2	4	2	1	1	1	3	2	1	2	1	1	2
122	ホシユタカ	Hoshiyutaka	Japan(Hiroshima)	-			2	1	2	2	4	1	2	1	2	1	1	1	1	1	1	2	1	1	2	2	1	1	1	1	1	1	1	1	1	2	1	1	1	1	1	1	1	1	1	1	1
123	細稈	Hosogara	Japan(Aomori)	JRC 20	✓		1	1	1	2	4	1	2	2	1	1	1	2	1	1	1	2	1	1	2	1	1	1	4	1	1	2	1	2	1	3	2	4	1	1	3	1	1	1	1	1	1
124	ホウヨク	Houyoku	Japan(Fukuoka)	-	✓		1	1	2	1	4	1	2	2	1	1	1	1	1	1	1	4	1	1	1	1	1	1	2	1	1	1	1	1	1	1	1	4	1	1	3	1	1	1	1	1	1
125	兵庫錦	Hyogonishiki	Japan(Hyogo)	-			1	1	2	1	4	1	1	2	1	1	1	2	1	1	1	2	1	1	1	1	1	4	2	1	1	1	1	1	1	2	1	4	1	1	3	1	1	2	2	1	1
126	胆振早稲	Iburiwase	Japan(Hokkaido)	-			1	1	1	2	4	1	2	2	1	1	1	1	1	1	1	2	1	1	2	2	1	3	12	1	2	2	6	1	2	4	2	1	1	1	3	1	1	1	1	1	1
127	一番星	Ichibamboshi	Japan(Ibaraki)	-	✓		1	1	1	2	1	1	1	1	1	1	1	4	2	1	1	1	3	1	1	1	1	1	4	1	1	2	1	1	2	1	1	2	1	1	3	1	1	2	1	1	2
128	イクヒカリ	Ikuhikari	Japan(Fukui)	-			3	2	1	2	1	1	1	2	1	1	1	4	2	2	1	1	3	2	1	1	1	1	1	1	1	1	1	2	1	1	1	1	1	1	1	1	1	1	1	1	2
129	イナバワセ	Inabawase	Japan(Niigata)	-			3	2	1	2	1	1	1	1	1	1	1	4	2	2	1	1	1	2	1	2	1	1	4	1	1	2	1	1	1	2	1	1	1	1	3	1	1	1	1	2	2
130	いなほっこり	Inahokkori	Japan(Hiroshima)	-			1	1	1	2	1	1	1	1	1	1	1	4	2	2	1	1	3	2	1	2	1	1	1	1	1	1	1	2	2	1	1	2	1	1	3	2	1	2	1	2	2
131	IR 24	IR 24	Philippines	-			5	1	3	2	3	1	2	2	3	2	1	3	1	1	2	3	2	1	2	1	1	4	7	3	2	2	4	1	3	5	1	3	2	8	1	1	2	1	1	1	1
132	IR 36	IR 36	Philippines	-			5	1	3	2	3	2	2	2	3	2	2	3	1	1	2	3	2	1	2	3	2	5	7	3	2	2	4	1	3	5	1	3	2	8	1	1	2	1	1	1	1
133	IR 58	IR 58	Philippines	WRC 14			5	1	3	2	3	1	2	2	3	2	2	3	1	1	2	3	2	1	2	3	1	1	9	3	2	2	4	1	3	5	1	5	2	6	1	1	2	1	1	1	1
134	IR 64	IR 64	Philippines	-	✓		5	1	3	2	3	2	2	2	3	2	2	3	1	1	2	3	2	1	2	1	1	4	9	3	2	2	4	1	3	5	1	3	2	6	1	1	2	1	1	1	1
135	入間錦	Irumanishiki	Japan(Saitama)	JRC 07	✓		1	1	1	2	4	1	2	2	2	1	1	4	1	1	1	4	1	1	2	2	1	1	12	1	2	2	1	1	2	2	1	2	2	1	3	1	1	1	1	1	1
136	石白	Ishijiro	Japan(Toyama)	JRC 23			1	2	1	1	4	1	1	2	1	1	1	1	1	1	1	1	1	1	1	1	1	1	2	1	2	1	3	2	2	2	1	1	1	1	3	1	1	2	1	1	1
137	石狩白毛	Ishikarishiroke	Japan(Hokkaido)	-			1	1	1	2	4	1	1	2	1	1	1	4	1	1	1	2	1	2	1	2	1	3	12	1	1	2	6	1	2	4	1	2	1	1	3	1	1	2	1	1	1
138	いただき	Itadaki	Japan(Niigata)	-	✓		1	2	1	2	1	1	2	2	1	1	1	4	2	2	1	1	3	1	1	2	1	1	2	1	1	2	1	1	1	1	1	2	1	1	1	1	1	1	1	1	2
139	Italica Livorno	Italica Livorno	Italy	-			1	1	2	2	4	1	2	2	1	1	1	4	1	1	1	2	1	1	2	1	1	1	13	2	1	2	8	1	2	2	1	1	1	1	3	1	1	1	2	1	1
140	いわいだわら	Iwaidawara	Japan(Akita)	-			1	1	1	1	4	1	1	1	1	1	1	4	2	1	1	1	1	1	2	1	1	4	4	3	1	2	3	2	1	2	1	1	1	1	1	2	1	1	1	1	1
141	泉348	Izumi 348	Japan(Unknown)	-			1	2	1	1	4	1	1	2	1	1	1	1	1	1	1	2	2	1	1	1	1	4	5	3	1	1	4	1	1	2	1	4	1	1	3	1	1	2	1	1	1
142	Jarjan	Jarjan	Bhutan	WRC 28	✓		4	1	4	2	3	1	2	2	3	2	1	3	1	1	2	3	2	1	2	3	1	2	14	2	2	2	5	1	3	5	1	3	2	9	1	1	2	1	2	1	1
143	上州	Joshuu	Japan(Yamagata)	JRC 24	✓		1	1	1	2	4	1	1	1	1	1	1	2	1	1	1	2	1	1	1	1	1	1	1	1	1	1	1	2	2	3	1	4	1	1	1	1	1	2	1	2	1
144	十石	Jukkoku	Japan(Unknown)	-			1	1	1	1	4	1	1	1	1	1	1	1	1	1	1	1	1	1	1	2	1	1	2	1	1	1	1	1	2	1	1	1	1	1	1	1	1	2	1	2	1
145	かばしこ	Kabashiko	Japan(Miyazaki)	JRC 35			1	1	2	2	4	2	1	2	1	1	1	2	1	1	1	2	1	1	1	1	1	1	2	1	2	1	1	1	2	3	1	2	2	1	3	1	1	1	2	2	1
146	嘉平	Kahei	Japan(Kagoshima)	JRC 11	✓		1	1	2	2	4	1	2	2	2	1	1	4	1	1	1	2	1	1	2	2	1	1	11	1	1	2	1	1	2	2	1	2	2	1	1	1	2	1	1	2	1
147	亀治	Kameji	Japan(Shimane)	JRC 31	✓		1	1	1	1	4	1	1	2	1	1	1	2	1	1	1	4	1	1	1	1	1	1	1	1	2	1	1	2	1	2	1	4	1	1	3	1	1	2	2	2	1
148	亀の尾	Kamenoo	Japan(Unknown)	-	✓		3	2	1	2	1	1	1	2	1	1	1	1	1	1	1	1	3	2	1	2	1	3	2	1	1	2	3	2	2	3	1	4	1	1	3	1	1	2	2	1	1
149	亀の尾4号	Kamenoo 4	Japan(Aomori)	-			1	1	1	1	4	1	2	2	2	1	1	4	2	2	1	4	1	1	2	1	1	1	4	1	1	2	1	2	1	3	2	4	1	1	3	1	1	1	1	1	2
150	金子b	Kaneko b	Japan(Kanto Tozan)	JRC 06	✓		2	1	2	2	4	1	2	2	2	1	1	4	1	1	1	2	1	1	2	1	1	1	11	2	2	2	1	2	2	9	2	4	1	1	1	1	2	1	1	1	1

(Continued)

Cultivar No.	Cultivar name (Kanji and Kana in Japanese)	Cultivar name (listed alphabetically)	Origin(Area)	WRC/JRC number[a]	Cultivars used in assay for primary screening	OsGI	FA0057	LOC_Os01g62780	FA0118	OsMADS51	OsVIL2	FA2420	OsCOL4	DTH2	Ehd4	Ef-cd	OsMADS50	FA0192	FA0193	DTH3b	Hd6	Hd16	FA1067	OsHDT1	Hd17	RFT1	Hd3a	Hd1	Se5	OsFTIP1	FA0419	Ghd7	FA0444	OsMADS18	PRR37	Ehd3	Hd18	OsLHY	DTH8	FA2167-FA0494	Ehd2	Ehd1	FA0603	LOC_Os11g08410	FA1674	FA1759
					Alleles at 41 loci: Locus name																																									
					No.[b]	1	2	3	4	5	6	7	8	9	10	11	12	13	14	15	16	17	18	19	20	21	22	23	24	25	26	27	28	29	30	31	32	33	34	35	36	37	38	39	40	41
					No.[c]	[1]	-	[2]	-	[3]	[4]	-	[5]	[6]	[7]	[8]	[9]	-	-	[10]	[11]	[12]	-	[13]	[14]	[15]	[16]	[17]	[18]	[19]	-	[20]	-	[21]	[22]	[23]	[24]	[25]	[26]	-	[27]	[28]	-	[29]	-	-
					No.[d]	-	1	-	2	-	-	3	-	-	-	-	-	4	5	-	-	-	6	-	-	-	-	-	-	-	7	-	8	-	-	-	-	-	-	9	-	-	10	-	11	12
151	関東212号	Kanto 212	Japan(Ibaraki)	-	✓	3	2	1	2	1	1	1	2	1	1	1	4	2	2	1	1	1	1	1	2	1	1	4	1	1	2	1	1	2	3	1	2	1	1	3	1	1	1	1	2	1
152	関東HD2号	Kanto HD 2	Japan(Ibaraki)	-		3	2	1	2	1	1	1	1	1	1	1	4	2	2	1	1	3	2	1	2	1	1	1	1	1	2	1	1	2	1	1	1	1	9	3	1	1	2	1	2	2
153	唐法師	Karahoshi	Japan(Kagoshima)	JRC 44		4	1	4	2	2	2	2	2	3	2	1	3	1	1	2	3	2	1	2	3	1	2	3	3	2	2	4	1	3	6	1	3	2	8	1	1	2	1	1	1	1
154	Kasalath	Kasalath	India	WRC 02	✓	4	1	4	2	3	2	2	2	3	2	1	3	1	1	2	3	2	1	2	3	1	2	9	2	2	2	5	1	2	8	1	3	2	9	1	1	2	1	2	1	1
155	カツラワセ	Katsurawase	Japan(Kagoshima)	-		1	1	1	1	1	1	1	1	1	1	1	2	1	1	1	1	1	1	2	2	1	1	9	1	1	2	1	1	2	2	1	1	1	1	1	2	1	2	1	2	2
156	Khao Nam Jen	Khao Nam Jen	Laos	WRC 68	✓	2	1	2	2	4	1	2	2	2	1	1	4	1	1	1	2	1	1	2	2	1	1	8	2	1	2	1	1	2	2	1	2	2	1	2	1	1	1	1	1	1
157	Khau Mac Kho	Khau Mac Kho	Vietnam	WRC 48	✓	2	3	2	2	4	1	2	2	2	1	1	4	1	1	1	2	1	1	2	2	1	1	10	1	2	2	1	1	2	5	1	2	2	1	1	1	1	1	1	1	1
158	喜峰	Kihou	Japan(Aichi)	-	✓	1	1	1	1	4	2	1	1	1	1	1	1	1	1	1	1	1	1	1	1	1	4	4	1	1	1	3	2	2	1	1	2	1	1	1	1	1	1	1	2	2
159	金南風	Kinmaze	Japan(Aichi)	-	✓	1	1	2	1	4	2	2	2	1	1	1	1	1	1	1	2	1	1	1	1	1	1	2	1	1	1	3	2	2	1	1	4	1	1	1	1	1	2	1	2	1
160	Kinangdong Patong	Kinandang Patong	Philippines	-	✓	2	1	2	2	4	1	2	2	2	1	1	4	1	1	1	2	1	1	2	2	1	1	9	2	2	2	1	1	2	2	1	2	2	1	1	1	2	1	1	2	1
161	キヌヒカリ	Kinuhikari	Japan(Niigata)	-	✓	3	2	1	2	1	1	1	1	1	1	1	4	1	1	1	1	3	2	1	2	1	1	1	1	1	1	1	1	2	1	1	1	1	1	1	1	1	1	1	2	2
162	きぬむすめ	Kinumusume	Japan(Fukuoka)	-	✓	3	2	1	2	1	1	1	1	1	1	1	4	2	1	1	1	1	2	1	2	1	1	1	1	1	1	1	1	2	1	1	1	1	1	1	1	1	1	1	2	2
163	きぬのはだ	Kinunohada	Japan(Akita)	-		1	2	1	2	1	1	1	1	1	1	1	4	2	2	1	1	3	2	1	2	1	1	1	1	1	1	1	1	2	1	1	2	1	1	1	2	1	1	1	2	1
164	きらら397	Kirara 397	Japan(Hokkaido)	-	✓	1	1	1	1	4	1	2	2	1	1	1	4	2	1	1	1	1	1	2	1	1	3	2	1	1	2	6	1	1	4	2	1	1	1	3	1	1	2	1	1	2
165	キタアケ	Kitaake	Japan(Hokkaido)	-	✓	1	1	1	2	4	1	2	2	2	1	1	4	1	1	1	1	1	1	2	1	1	3	2	1	1	2	6	1	1	4	1	1	1	1	3	1	1	2	1	1	2
166	きたあおば	Kitaaoba	Japan(Hokkaido)	-		5	1	1	2	1	1	2	2	1	1	1	4	1	2	1	1	1	1	2	1	1	3	2	1	1	2	6	1	2	4	2	2	1	1	1	1	1	2	1	2	1
167	きたげんき	Kitagenki	Japan(Hokkaido)	-		1	1	1	2	4	1	2	2	1	1	1	2	1	1	1	1	1	1	2	2	1	3	2	1	1	2	6	1	2	4	1	1	1	1	3	1	1	2	1	1	1
168	キタヒカリ	Kitahikari	Japan(Hokkaido)	-	✓	3	1	1	2	4	1	2	1	2	1	1	4	1	1	1	1	1	2	2	1	1	3	12	1	1	2	6	1	1	4	2	1	1	1	3	2	1	2	1	1	2
169	きたくりん	Kitakurin	Japan(Hokkaido)	-		1	1	1	1	1	1	2	2	1	1	1	2	1	1	1	1	1	1	2	1	1	3	2	1	1	2	6	1	2	4	1	1	1	1	3	2	1	2	1	1	2
170	きたゆきもち	Kitayukimochi	Japan(Hokkaido)	-	✓	3	1	1	2	4	1	1	2	1	1	1	4	1	1	1	2	1	1	1	2	1	3	12	1	1	2	6	2	2	4	2	4	1	2	1	1	1	1	1	2	1
171	キヨニシキ	Kiyonishiki	Japan(Akita)	-	✓	3	2	1	1	1	2	2	2	1	1	1	1	2	2	1	1	1	1	2	1	1	1	4	1	1	2	3	1	1	3	1	2	1	1	1	2	1	2	1	2	1
172	コチヒビキ	Kochihibiki	Japan(Saitama)	-		1	1	1	1	1	1	1	1	1	1	1	1	1	1	1	1	1	1	1	1	1	4	2	1	1	2	3	2	2	1	1	1	1	1	1	1	1	2	1	2	1
173	黄金晴	Koganebare	Japan(Aichi)	-	✓	1	1	1	1	4	1	1	1	1	1	1	1	1	1	1	1	1	1	1	1	1	4	1	1	1	1	1	1	2	1	1	2	1	1	1	1	1	1	1	2	1
174	コガネマサリ	Koganemasari	Japan(Miyazaki)	-	✓	1	1	1	1	4	1	1	1	1	1	1	2	1	1	1	1	1	1	1	2	1	1	1	1	1	1	1	1	1	1	1	1	1	1	3	1	1	2	1	1	1
175	こがねもち	Koganemochi	Japan(Niigata)	-	✓	1	1	1	1	4	2	2	2	1	1	1	4	2	2	1	1	3	1	2	2	1	1	2	1	1	1	3	2	2	3	1	4	1	1	3	1	1	2	1	1	2
176	黄金錦	Koganenishiki	Japan(Aichi)	-		1	1	1	1	4	1	1	1	1	1	1	2	1	1	1	1	1	1	1	2	1	1	1	1	1	1	1	1	1	1	1	2	1	1	3	2	1	2	1	2	1
177	恋の予感	Koinoyokan	Japan(Hiroshima)	-		1	2	1	2	1	1	1	1	1	1	1	4	2	1	1	1	1	2	1	2	1	1	1	1	1	1	1	1	2	1	1	2	1	1	1	1	1	1	1	2	1
178	恋初めし	Koisomeshi	Japan(Hiroshima)	-		2	2	1	2	1	1	1	1	1	1	1	4	2	2	1	1	1	2	1	2	1	1	1	1	1	1	1	2	2	1	1	1	1	1	1	1	1	2	1	2	2
179	こなゆきの舞	Konayukinomai	Japan(Niigata)	-		1	2	1	1	4	1	1	1	1	1	1	4	2	2	1	1	1	1	2	2	1	1	4	1	1	2	3	2	2	3	1	1	1	1	1	1	1	2	1	2	1
180	コシヒカリ	Koshihikari	Japan(Fukui)	-	✓	3	2	1	2	1	1	1	1	1	1	1	4	2	2	1	1	3	2	1	2	1	1	1	1	1	2	1	1	2	1	1	1	1	1	3	1	1	2	1	2	2
181	コシヒカリ環1号	Koshihikari Kan 1	Japan(Ibaraki)	-	✓	3	2	1	2	1	1	1	1	1	1	1	4	2	2	1	1	3	2	1	2	1	1	1	1	1	2	1	1	2	1	1	1	1	1	3	1	1	2	1	2	2
182	コシホマレ	Koshihomare	Japan(Niigata)	-	✓	1	1	1	1	4	2	1	2	1	1	1	4	2	2	1	2	1	1	1	1	1	4	4	1	1	2	1	1	2	3	1	2	1	1	1	2	1	1	1	2	2
183	こしいぶき	Koshiibuki	Japan(Niigata)	-	✓	3	2	1	2	1	1	1	1	1	1	1	4	2	1	1	1	1	1	1	2	1	4	4	1	1	2	1	1	2	1	1	2	1	1	3	1	1	2	1	2	2
184	越路早生	Koshijiwase	Japan(Niigata)	-	✓	1	1	1	1	4	1	1	2	1	1	1	1	1	1	1	1	1	2	1	2	1	1	4	1	1	1	1	1	2	3	1	1	1	1	3	1	1	2	1	2	1
185	越のかおり	Koshinokaori	Japan(Niigata)	-		3	2	1	2	1	1	1	1	1	1	1	4	1	1	1	1	3	2	1	3	1	2	1	1	1	1	1	1	2	1	1	1	1	1	1	1	1	1	1	2	2
186	くらのぬし	Kuranonushi	Japan(Ibaraki)	-		5	1	3	2	3	1	2	2	3	1	1	1	1	1	2	2	2	1	2	3	1	1	7	3	2	2	4	1	3	5	1	3	2	1	3	1	2	1	1	1	1
187	クサホナミ	Kusahonami	Japan(Ibaraki)	-		5	1	2	1	4	1	1	1	1	1	1	1	1	1	1	1	1	1	2	2	1	1	1	1	1	1	1	2	1	1	1	1	1	1	3	1	1	2	1	1	1
188	クサノホシ	Kusanohoshi	Japan(Hiroshima)	-		5	1	2	1	4	1	1	1	1	1	1	1	1	1	1	1	1	1	2	2	1	1	2	1	1	2	1	1	1	2	1	1	1	1	1	2	1	2	1	1	1
189	クサユタカ	Kusayutaka	Japan(Niigata)	-		1	1	1	1	1	1	1	1	1	1	1	4	2	2	1	2	1	1	1	2	1	1	4	1	1	1	3	1	1	2	1	1	1	1	1	1	1	2	1	1	1
190	京都旭	Kyotoasahi	Japan(Kyoto)	JRC 34		1	1	2	1	4	2	2	2	1	1	1	1	1	1	1	2	1	1	1	1	1	1	2	1	1	1	1	1	1	1	1	2	1	1	1	1	1	2	1	1	1
191	LAC23	LAC23	Nigeria	-	✓	2	1	2	2	4	1	2	2	2	1	1	4	1	1	1	2	1	1	2	2	1	1	9	2	2	2	1	1	2	2	2	2	2	1	1	1	2	1	1	3	1
192	レーク65	Lake 65	Japan(Shiga)	-	✓	3	2	1	2	1	1	1	1	1	1	1	4	2	2	1	1	3	2	1	2	1	1	1	1	1	1	1	1	2	1	1	1	1	1	3	1	1	1	1	2	2
193	リーフスター	Leafstar	Japan(Ibaraki)	-		3	2	1	2	1	1	1	1	2	1	1	2	1	1	1	1	3	1	2	2	1	1	1	1	1	2	1	1	2	5	1	4	1	1	3	1	1	2	1	1	1
194	LTH	Lijiangxintuanheigu	China	-		1	1	1	2	1	1	2	2	2	1	1	4	1	1	1	2	1	1	2	2	1	1	8	2	1	2	1	1	2	2	1	2	2	1	1	1	1	1	2	1	1
195	まきみずほ	Makimizuho	Japan(Fukuoka)	-		5	1	1	2	1	1	2	1	1	1	1	4	2	2	1	2	1	1	1	2	1	1	4	1	1	1	3	1	1	2	1	1	1	1	1	1	1	2	1	1	1
196	まなむすめ	Manamusume	Japan(Miyagi)	-	✓	3	2	1	2	1	1	1	1	1	1	1	4	2	1	1	1	1	1	1	2	1	1	4	1	1	2	1	1	2	1	1	2	1	1	3	1	1	1	1	2	1
197	万作	Mansaku	Japan(Nagano)	JRC 22		1	1	1	2	4	1	2	2	1	1	1	4	2	1	1	2	1	1	2	1	1	1	2	1	1	2	1	2	1	3	2	2	1	1	3	1	1	1	1	1	1
198	まっしぐら	Masshigura	Japan(Akita)	-	✓	3	2	1	1	4	1	1	2	1	1	1	2	1	1	1	1	3	1	1	2	1	1	4	1	1	2	1	1	1	1	1	2	1	1	3	2	1	1	1	2	1
199	祭り晴	Matsuribare	Japan(Aichi)	-	✓	1	1	1	1	1	1	1	1	1	1	1	4	2	1	1	1	1	1	1	1	1	1	1	1	1	1	3	2	2	1	1	1	1	1	1	1	1	2	1	2	2
200	目黒糯	Meguromochi	Japan(Kanto Tozan)	JRC 14	✓	4	1	2	2	4	1	2	2	2	1	1	4	1	1	1	2	1	1	2	2	1	1	1	1	1	2	1	1	2	2	1	4	1	1	1	1	1	1	1	2	1

(Continued)

Cultivar No.	Cultivar name (Kanji and Kana in Japanese)	Cultivar name (listed alphabetically)	Origin(Area)	WRC/JRC number[a]	Cultivars used in assay for primary screening	OsGI	FA0057	LOC_Os01g62780	FA0118	OsMADS51	OsVIL2	FA2420	OsCOL4	DTH2	Ehd4	Ef-cd	OsMADS50	FA0192	FA0193	DTH3b	Hd6	Hd16	FA1067	OsHDT1	Hd17	RFT1	Hd3a	Hd1	Se5	OsFTIP1	FA0419	Ghd7	FA0444	OsMADS18	PRR37	Ehd3	Hd18	OsLHY	DTH8	FA2167-FA0494	Ehd2	Ehd1	FA0603	LOC_Os11g08410	FA1674	FA1759
					Alleles at 41 loci: No.[b]	1	2	3	4	5	6	7	8	9	10	11	12	13	14	15	16	17	18	19	20	21	22	23	24	25	26	27	28	29	30	31	32	33	34	35	36	37	38	39	40	41
					No.[c]	[1]	-	[2]	-	[3]	[4]	-	[5]	[6]	[7]	[8]	[9]	-	-	[10]	[11]	[12]	-	[13]	[14]	[15]	[16]	[17]	[18]	[19]	-	[20]	-	[21]	[22]	[23]	[24]	[25]	[26]	-	[27]	[28]	-	[29]	-	-
					No.[d]	-	1	-	2	-	-	3	-	-	-	-	-	4	5	-	-	-	6	-	-	-	-	-	-	-	7	-	8	-	-	-	-	-	-	9	-	-	10	-	11	12
201	めんこいな	Menkoina	Japan(Akita)	-	✓	3	2	1	2	1	1	1	1	1	1	1	4	2	2	1	1	1	1	1	2	1	1	4	1	1	2	1	1	1	2	1	2	1	1	3	1	1	2	1	2	1
202	めんこもち	Menkomochi	Japan(Aomori)	-		3	2	1	1	4	1	1	2	1	1	1	2	1	1	1	1	3	1	2	2	1	1	4	1	1	2	1	1	1	3	1	2	1	1	3	1	1	1	1	2	2
203	三重23号	Mie 23	Japan(Mie)	-	✓	3	2	1	2	1	1	2	1	1	1	1	4	2	2	1	1	1	1	1	2	1	1	4	1	1	2	1	2	2	1	1	2	1	1	3	1	1	2	1	2	1
204	みえのゆめ	Mienoyume	Japan(Mie)	-		1	1	1	1	1	1	1	1	1	1	1	4	2	1	1	1	1	1	1	1	1	1	1	1	1	1	1	2	2	1	1	1	1	1	3	1	1	2	1	1	1
205	ミルキークイーン	Milkyqueen	Japan(Ibaraki)	-		3	2	1	2	1	1	1	1	1	1	1	4	2	2	1	1	3	2	1	2	1	1	1	1	1	2	1	1	2	1	1	1	1	1	3	1	1	2	1	2	2
206	密陽23号	Milyang 23	Korea	WRC 57		5	1	3	2	3	1	2	2	3	1	1	1	1	1	2	2	2	1	2	1	1	4	7	3	2	2	4	1	3	5	1	3	2	1	1	1	2	1	1	1	1
207	みなちから	Minachikara	Japan(Hiroshima)	-		3	2	1	1	4	1	1	2	1	1	1	1	1	1	1	2	1	1	2	2	1	1	4	1	1	2	4	1	1	5	1	4	1	1	3	1	2	2	1	1	1
208	ミネアサヒ	Mineasahi	Japan(Aichi)	-	✓	1	2	1	2	1	1	1	1	1	1	1	4	2	2	1	1	1	1	1	2	1	1	4	1	1	2	3	2	2	1	1	2	1	1	1	1	1	2	1	2	2
209	みねはるか	Mineharuka	Japan(Aichi)	-		3	2	1	2	1	1	1	1	1	1	1	4	2	2	1	1	1	1	1	2	1	1	4	1	1	2	1	2	2	1	1	2	1	1	1	1	1	2	1	2	1
210	ミレニシキ	Mirenishiki	Japan(Ibaraki)	-	✓	2	1	2	1	1	1	1	1	1	1	1	4	2	2	1	1	1	1	1	1	1	1	1	1	1	2	1	1	2	1	1	1	1	1	3	1	1	2	1	1	2
211	ミズハタモチ	Mizuhatamochi	Japan(Ibaraki)	-	✓	1	1	1	1	4	1	2	2	1	1	1	4	1	1	1	1	1	1	1	2	1	1	11	1	1	1	1	1	2	2	2	4	1	1	3	1	1	2	1	2	1
212	ミズホチカラ	Mizuhochikara	Japan(Fukuoka)	-		3	2	1	1	4	1	1	2	1	1	1	1	1	1	1	2	2	1	2	1	1	4	5	3	1	2	4	1	1	2	1	4	1	1	3	1	3	2	1	1	1
213	みずほの輝き	Mizuhonokagayaki	Japan(Niigata)	-		3	2	1	2	1	1	1	2	1	1	1	4	2	2	1	1	3	1	1	2	1	1	1	1	1	2	1	1	2	1	1	2	1	1	1	1	1	2	1	1	1
214	みずかがみ	Mizukagami	Japan(Shiga)	-	✓	3	2	1	2	1	1	1	1	1	1	1	4	2	2	1	1	1	2	1	2	1	1	4	1	1	1	1	1	1	1	1	1	1	1	3	1	1	2	1	2	1
215	もちだわら	Mochidawara	Japan(Ibaraki)	-		5	1	3	2	3	1	2	2	3	1	1	1	1	1	2	2	2	1	2	3	1	1	7	3	2	2	4	1	3	5	1	3	2	1	3	1	2	1	1	1	1
216	Modan	Modan	India	-		4	1	4	2	2	2	2	2	2	2	2	5	1	3	1	3	2	1	2	3	1	2	9	2	2	2	1	1	2	2	1	3	2	9	1	1	2	1	2	1	1
217	萌えみのり	Moeminori	Japan(Akita)	-		1	1	1	1	1	1	1	1	1	1	1	4	2	1	1	1	1	1	1	2	1	1	4	1	1	1	1	1	2	3	1	2	1	1	1	2	1	2	1	2	2
218	モグモグあおば	Mogumoguaoba	Japan(Fukuoka)	-		5	1	1	2	1	1	2	1	1	1	1	1	2	2	1	2	1	1	2	2	1	1	5	3	1	2	4	1	1	2	1	1	1	1	3	1	3	2	1	1	1
219	モミロマン	Momiroman	Japan(Ibaraki)	-		3	2	1	1	4	1	1	2	1	1	1	1	1	1	1	2	1	1	2	2	1	1	5	3	1	2	4	1	1	2	1	4	1	1	3	1	3	2	1	1	1
220	もみゆたか	Momiyutaka	Japan(Aichi)	-		4	1	4	2	3	1	2	2	3	2	1	3	1	1	2	3	2	1	2	2	1	1	6	3	2	2	4	1	3	5	1	3	2	1	3	1	2	1	1	1	1
221	森多早生	Moritawase_a*	Japan(Yamagata)	-		1	1	1	1	4	1	2	2	1	1	1	2	1	1	1	1	3	1	2	1	1	1	1	1	1	2	1	2	1	3	1	2	1	1	3	1	1	2	1	1	1
222	森田早生	Moritawase_b*	Japan(Yamagata)	-	✓	1	2	1	1	4	1	1	2	1	1	1	1	1	1	1	1	1	1	1	1	1	1	1	1	1	1	3	2	2	3	1	2	2	1	3	1	1	2	2	1	1
223	Muha	Muha	India	WRC 25	✓	4	1	4	2	3	1	2	2	3	2	1	3	1	1	1	3	2	1	2	3	1	2	9	2	2	2	1	1	2	5	2	2	2	7	1	1	2	1	2	1	1
224	むつほまれ	Mutsuhomare	Japan(Aomori)	-	✓	3	2	1	1	4	1	1	2	1	1	1	2	1	1	1	1	1	1	2	1	1	1	4	1	1	2	1	1	2	3	1	2	1	1	3	1	1	1	1	2	1
225	Naba	Naba	India	WRC 05	✓	4	1	3	2	3	2	2	2	3	2	1	3	1	1	2	2	2	1	2	3	1	2	9	4	2	2	4	1	3	5	1	3	2	3	1	1	2	1	1	1	1
226	名古屋白	Nagoyashiro	Japan(Akita)	JRC 38		1	1	1	2	4	2	2	2	1	1	1	4	2	1	1	4	1	1	2	1	1	3	1	1	1	2	1	1	1	3	1	2	2	1	3	1	1	1	2	1	1
227	ナゴユタカ	Nagoyutaka	Japan(Niigata)	-	✓	3	2	1	2	4	1	1	2	1	1	1	2	1	1	1	1	1	1	1	2	1	1	4	1	1	1	1	2	1	1	1	2	1	1	1	1	1	1	1	2	2
228	中生新千本	Nakateshinsenbon	Japan(Aichi)	-	✓	1	1	2	1	4	2	2	2	1	1	1	1	1	1	1	2	1	1	1	1	1	1	2	1	1	1	3	2	2	1	1	4	1	1	1	2	1	2	1	2	1
229	ななつぼし	Nanatsuboshi	Japan(Hokkaido)	-	✓	3	1	1	2	1	1	2	2	1	1	1	2	1	1	1	1	1	1	2	1	1	3	2	1	1	2	6	1	2	4	2	2	1	1	3	1	1	2	1	2	1
230	なすひかり	Nasuhikari	Japan(Tochigi)	-	✓	3	2	1	2	1	1	1	1	1	1	1	4	1	1	1	1	1	2	1	2	1	1	4	1	1	2	1	1	2	1	1	2	1	1	1	1	1	1	1	2	2
231	なつあおば	Natsuaoba	Japan(Niigata)	-		5	1	1	1	4	1	1	1	1	1	1	2	1	1	1	2	1	1	1	1	1	1	4	1	1	2	1	1	1	5	1	4	1	1	3	2	1	1	1	1	2
232	ナツヒカリ	Natsuhikari	Japan(Kagoshima)	-	✓	1	1	1	1	1	2	1	1	1	1	1	2	1	1	1	1	1	1	1	2	1	1	9	1	1	2	1	1	2	2	1	1	1	1	1	2	1	2	1	2	2
233	なつほのか	Natsuhonoka	Japan(Kagoshima)	-		3	2	1	2	1	1	1	1	1	1	1	4	2	2	1	1	3	1	1	2	1	1	1	1	1	2	1	1	2	1	1	2	1	1	3	1	1	1	1	2	2
234	なつのたより	Natsunotayori	Japan(Kagoshima)	-		3	2	1	1	1	1	1	1	1	1	1	2	1	1	1	1	3	1	2	2	1	1	4	1	1	2	1	1	2	2	1	1	1	1	1	2	1	2	1	2	2
235	ニホンマサリ	Nihonmasari	Japan(Saitama)	-	✓	1	1	2	1	1	1	1	1	1	1	1	1	1	1	1	1	1	1	1	1	1	4	1	1	1	2	3	2	2	1	1	1	1	1	1	1	1	1	1	2	1
236	新潟早生	Niigatawase	Japan(Niigata)	-		1	1	1	1	4	2	1	2	1	1	1	4	2	2	1	1	1	1	1	1	1	1	9	1	1	1	1	2	1	2	1	1	1	1	3	2	1	2	1	1	2
237	にじのきらめき	Nijinokirameki	Japan(Niigata)	-		3	2	1	2	1	1	1	1	1	1	1	4	2	2	1	1	3	1	1	2	1	1	1	1	1	1	1	1	2	1	1	2	1	1	3	2	1	1	1	2	2
238	にこまる	Nikomaru	Japan(Fukuoka)	-	✓	3	2	1	2	1	1	1	2	1	1	1	4	2	1	1	1	1	1	1	2	1	1	1	1	1	2	1	1	2	1	1	2	1	1	1	1	1	1	1	2	2
239	日本晴	Nipponbare	Japan(Aichi)	WRC 01	✓	1	1	1	1	1	1	1	1	1	1	1	1	1	1	1	1	1	1	1	1	1	1	1	1	1	1	1	1	1	1	1	1	1	1	1	1	1	1	1	1	1
240	ニシアオバ	Nishiaoba	Japan(Fukuoka)	-		1	1	1	1	1	1	1	1	1	1	1	4	2	2	1	2	1	1	1	1	1	4	2	1	1	2	3	1	1	2	1	1	1	1	1	1	1	1	1	1	1
241	ニシホマレ	Nishihomare	Japan(Miyazaki)	-	✓	1	1	2	1	1	1	2	2	1	1	1	1	1	1	1	4	1	1	1	1	1	1	2	1	1	1	1	1	1	1	1	1	1	1	3	1	1	2	1	1	1
242	Nona Bokra	Nona Bokra	India	-	✓	4	1	3	2	2	2	2	2	3	2	1	3	1	1	1	3	2	1	2	3	2	2	3	3	2	2	4	1	3	5	2	3	2	3	1	1	2	1	1	1	1
243	農林1号	Nourin 1	Japan(Niigata)	-	✓	3	2	1	2	1	1	2	2	1	1	1	4	2	2	1	1	3	2	1	2	1	1	4	1	1	2	3	2	2	3	1	4	1	1	3	1	1	2	1	1	2
244	農林16号	Nourin 16	Japan(Miyagi)	-		3	1	1	2	4	1	2	2	1	1	1	4	2	2	1	4	1	1	2	2	1	1	4	1	1	2	1	2	2	1	2	4	1	1	3	1	1	1	1	1	1
245	農林18号	Nourin 18	Japan(Kumamoto)	-	✓	1	1	2	1	4	2	2	2	1	1	1	1	1	1	1	2	1	2	1	1	1	1	2	1	1	1	1	2	1	1	1	4	2	1	1	1	1	2	1	1	1
246	農林22号	Nourin 22	Japan(Hyogo)	-	✓	1	1	1	1	4	1	1	1	1	1	1	1	1	1	1	1	1	1	1	2	1	1	1	1	1	1	1	1	2	1	1	1	1	1	1	1	1	2	1	2	1
247	農林29号	Nourin 29	Japan(Gifu)	-		1	1	1	1	1	1	1	1	1	1	1	1	1	1	1	2	1	1	1	1	1	1	1	1	1	1	1	1	1	2	1	4	1	1	1	1	1	2	1	2	1
248	農林6号	Nourin 6	Japan(Hyogo)	-	✓	1	1	1	1	4	1	1	1	1	1	1	1	1	1	1	2	1	1	1	1	1	1	1	1	1	1	1	2	2	1	1	4	1	1	3	1	1	2	1	2	1
249	農林8号	Nourin 8	Japan(Hyogo)	-	✓	1	1	1	1	1	1	1	1	1	1	1	1	1	1	1	1	1	1	1	2	1	1	2	1	1	1	1	1	1	2	1	1	1	1	1	1	1	2	1	1	1
250	能登ひかり	Notohikari	Japan(Ishikawa)	-	✓	1	1	1	1	1	1	1	2	1	1	1	4	2	2	1	1	1	2	1	2	1	4	9	2	2	1	1	1	2	1	1	1	1	1	3	1	1	2	1	2	1

(Continued)

Cultivar No.	Cultivar name (Kanji and Kana in Japanese)	Cultivar name (listed alphabetically)	Origin(Area)	WRC/JRC number[a]	Cultivars used in assay for primary screening		Alleles at 41 loci																																								
						No.[d]	-	1	-	2	-	-	3	-	-	-	-	-	4	5	-	-	-	6	-	-	-	-	-	-	-	7	-	8	-	-	-	-	-	-	9	-	-	10	-	11	12
						No.[c]	[1]	-	[2]	-	[3]	[4]	-	[5]	[6]	[7]	[8]	[9]	-	-	[10]	[11]	[12]	-	[13]	[14]	[15]	[16]	[17]	[18]	[19]	-	[20]	-	[21]	[22]	[23]	[24]	[25]	[26]	-	[27]	[28]	-	[29]	-	-
						No.[b]	1	2	3	4	5	6	7	8	9	10	11	12	13	14	15	16	17	18	19	20	21	22	23	24	25	26	27	28	29	30	31	32	33	34	35	36	37	38	39	40	41
						Locus name	OsGI	FA0057	LOC_Os01g62780	FA0118	OsMADS51	OsVIL2	FA2420	OsCOL4	DTH2	Ehd4	Ef-cd	OsMADS50	FA0192	FA0193	DTH3b	Hd6	Hd16	FA1067	OsHDT1	Hd17	RFT1	Hd3a	Hd1	Se5	OsFTIP1	FA0419	Ghd7	FA0444	OsMADS18	PRR37	Ehd3	Hd18	OsLHY	DTH8	FA2167-FA0494	Ehd2	Ehd1	FA0603	LOC_Os11g08410	FA1674	FA1759
251	大場	Ouba	Japan(Ishikawa)	-	✓		1	1	1	1	4	1	1	2	1	1	1	1	1	1	1	1	1	2	2	1	1	1	2	1	1	1	3	2	2	3	2	4	1	1	1	1	1	1	1	1	1
252	オオチカラ	Oochikara	Japan(Niigata)	-	✓		3	2	1	2	1	1	1	1	1	1	1	4	2	2	1	2	1	1	1	2	1	1	4	1	1	2	3	1	1	2	1	1	1	1	1	1	1	1	1	1	1
253	おどろきもち	Odorokimochi	Japan(Ibaraki)	-			5	1	4	2	3	1	2	2	3	1	1	1	1	1	2	2	2	1	2	1	1	1	7	3	2	2	4	1	3	5	1	3	2	1	3	1	2	1	1	1	1
254	おいらん	Oiran	Japan(Kumamoto)	JRC 12			1	1	2	2	1	1	2	2	2	1	1	4	1	1	1	2	1	1	2	2	1	1	11	2	1	2	1	1	2	2	1	2	2	1	1	1	2	1	1	2	1
255	岡穂	Okabo	Japan(Unknown)	JRC 47			4	1	2	2	4	1	2	2	2	1	1	4	1	1	1	2	1	1	2	2	1	1	1	1	1	2	1	1	2	2	2	4	1	1	1	1	1	1	1	2	1
256	おきにいり	Okiniiri	Japan(Akita)	-	✓		3	2	1	2	1	1	1	2	1	1	1	4	2	2	1	1	1	1	1	2	1	1	4	1	1	2	1	1	2	3	1	2	1	1	3	1	1	1	1	2	1
257	オッカモドシ	Okkamodoshi	Japan(Unknown)	JRC 08			1	1	2	2	1	1	2	2	2	1	1	4	1	1	1	2	1	1	2	2	1	1	11	1	1	2	1	1	2	2	1	2	2	1	1	1	1	1	1	2	1
258	雄町	Omachi	Japan(Okayama)	JRC 32	✓		1	1	2	2	1	1	2	2	1	1	1	2	1	1	1	2	1	2	2	1	1	4	2	1	1	1	1	1	1	1	1	2	1	1	1	1	1	1	2	1	1
259	オオナリ	Oonari	Japan(Ibaraki)	-			5	1	4	2	3	1	2	2	3	1	1	1	1	1	2	2	2	1	2	1	1	1	7	3	2	2	4	1	3	5	1	3	2	1	3	1	2	1	1	1	1
260	オオセト	Ouseto	Japan(Hiroshima)	-			1	1	1	1	4	1	1	1	1	1	1	1	1	1	1	1	1	1	1	1	1	1	2	1	1	1	1	2	2	1	1	1	1	1	1	1	1	2	1	2	1
261	オトメモチ	Otomemochi	Japan(Akita)	-			1	1	1	1	4	1	2	2	1	1	1	1	1	1	1	1	3	1	2	2	1	1	4	1	1	1	3	1	2	3	1	4	1	1	3	1	1	2	1	1	1
262	奥羽197号	Ouu 197	Japan(Akita)	-	✓		1	1	1	1	4	1	2	2	1	1	1	4	2	2	1	4	1	1	2	1	1	1	4	1	1	1	1	2	2	1	1	2	1	1	3	1	1	1	1	1	1
263	奥羽292号	Ouu 292	Japan(Akita)	-	✓		1	1	1	1	4	1	1	2	1	1	1	4	2	2	1	1	1	1	1	2	1	1	4	1	1	1	1	1	2	2	1	1	1	1	1	2	1	1	1	1	1
264	オワリハタモチ	Owarihatamochi	Japan(Mie)	-	✓		2	1	2	2	4	1	2	2	2	1	1	4	1	1	1	2	1	1	2	2	1	1	11	1	1	2	1	1	2	2	2	4	1	1	1	1	2	1	1	2	1
265	雄山錦	Oyamanishiki	Japan(Toyama)	-	✓		1	1	1	1	4	2	2	2	1	1	1	4	1	1	1	1	1	1	1	1	1	1	4	1	1	1	1	1	1	2	1	2	1	1	1	1	1	2	1	1	2
266	プリンセスサリー	Prinscess sari	Japan(Ibaraki)	-			1	1	1	1	1	1	2	1	1	1	1	1	1	1	1	1	1	1	1	1	1	4	1	1	1	1	1	1	1	1	1	1	2	1	1	1	2	1	1	1	1
267	Qiu Zhao Zong	Qiu Zhao Zong	China	WRC 10	✓		4	1	4	2	2	2	2	2	3	2	1	3	1	1	2	3	1	1	2	3	1	1	3	3	2	2	8	1	3	6	1	3	2	4	1	1	2	1	1	1	1
268	雷電	Raiden	Japan(Kanto Tozan)	JRC 53			1	1	2	2	1	1	2	2	1	1	1	2	1	1	1	2	1	2	2	1	1	4	2	1	1	1	1	1	1	1	1	2	1	1	1	1	1	1	2	1	1
269	レイホウ	Reihou	Japan(Fukuoka)	-	✓		1	1	2	1	4	1	2	2	1	1	1	1	1	1	1	4	1	1	1	1	1	1	2	1	1	1	1	1	1	1	1	4	1	1	3	1	1	2	1	1	1
270	レイメイ	Reimei	Japan(Aomori)	-	✓		1	1	1	1	4	1	2	2	1	1	1	2	1	1	1	2	1	1	2	1	1	1	4	1	1	2	1	1	1	2	1	4	1	1	3	2	1	1	1	2	2
271	茘支江	Reishikou	China	-	✓		4	1	1	1	4	1	1	2	1	1	1	2	1	1	1	2	1	1	1	2	1	1	1	1	2	1	1	2	1	2	1	4	1	1	1	1	1	2	2	1	1
272	陸羽132号	Rikuu 132	Japan(Akita)	-	✓		3	1	1	2	1	1	2	2	1	1	1	4	2	2	1	4	1	2	1	2	1	1	4	1	1	2	1	2	1	3	2	4	1	1	3	1	1	2	1	1	2
273	陸羽20号	Rikuu 20	Japan(Akita)	-			3	1	1	2	1	1	1	1	1	1	1	1	1	1	1	4	1	2	1	2	1	1	2	1	1	2	7	2	2	1	1	2	2	1	3	1	1	2	1	1	1
274	陸稲陸羽2号	Rikutorikuu 2	Japan(Unknown)	JRC 49			1	1	1	1	4	1	2	2	2	1	1	4	2	2	1	4	1	1	2	1	1	1	4	1	1	2	1	2	1	3	2	4	1	1	3	1	1	1	1	1	2
275	幸風	Sachikaze	Japan(Aichi)	-	✓		1	1	2	1	4	1	1	1	1	1	1	1	1	1	1	1	1	1	1	2	1	1	1	1	1	1	1	1	1	1	1	4	1	1	1	2	1	2	1	1	1
276	さきひかり	Sakihikari	Japan(Fukui)	-	✓		1	2	1	2	1	1	1	1	1	1	1	4	2	2	1	1	1	2	1	2	1	1	1	1	1	2	1	1	2	1	1	1	1	1	3	1	1	1	1	2	2
277	ソルトスター	Saltstar	Japan(Ibaraki)	-			5	1	1	1	3	2	2	2	3	2	1	3	1	1	2	3	2	1	2	3	2	2	7	1	1	2	4	1	3	5	1	3	2	3	3	1	2	1	1	1	1
278	ササニシキ	Sasanishiki	Japan(Miyagi)	-	✓		1	2	1	2	1	1	1	2	1	1	1	4	2	2	1	1	1	1	1	2	1	1	4	1	1	1	1	1	1	3	1	2	1	1	3	1	1	2	1	2	1
279	さとじまん	Satojiman	Japan(Ibaraki)	-			1	1	1	1	1	1	1	1	1	1	1	4	2	2	1	1	3	1	1	2	1	1	1	1	1	1	1	2	2	1	1	2	1	1	1	1	1	1	1	2	2
280	関取	Sekitori	Japan(Unknown)	-	✓		4	1	2	2	4	1	2	2	2	1	1	4	1	1	1	2	1	1	2	2	1	1	12	1	2	2	1	1	2	2	1	4	1	1	1	1	1	2	1	2	1
281	関山	Sekiyama	Japan(Aomori)	JRC 36	✓		1	1	1	1	4	2	1	2	1	1	1	2	1	1	1	2	1	1	2	1	1	1	12	1	1	2	1	1	1	3	1	2	1	1	3	1	1	1	1	1	1
282	戦捷	Senshou	Japan(Tokyo)	JRC 04	✓		2	1	2	2	4	1	2	2	2	1	1	4	1	1	1	2	1	1	2	2	1	1	11	1	1	2	1	1	2	2	2	4	1	1	1	1	2	2	1	2	1
283	せとのかがやき	Setonokagayaki	Japan(Hiroshima)	-			1	2	1	2	1	1	1	1	1	1	1	4	2	1	1	1	1	1	1	1	1	1	1	1	1	1	3	2	2	1	1	1	1	1	1	1	1	1	1	1	1
284	せとのにじ	Setononiji	Japan(Yamaguchi)	-			3	2	1	2	1	1	1	1	1	1	1	4	2	2	1	1	3	1	1	2	1	1	1	1	1	2	1	1	2	1	1	2	1	1	3	1	1	1	1	2	2
285	七面鳥糯	Shichimenchomochi	Japan(Unknown)	JRC 29			1	1	2	2	4	2	1	2	1	1	1	1	1	1	1	2	1	1	1	2	1	1	2	1	2	1	1	2	2	1	1	2	1	1	1	1	1	1	2	1	1
286	しふくのみのり	Shifukunominori	Japan(Akita)	-			1	1	1	1	1	1	1	1	1	1	1	4	2	1	1	1	1	1	2	2	1	1	4	1	1	2	3	2	2	3	1	2	1	1	3	2	1	2	1	2	2
287	滋賀羽二重糯	Shigahabutaemochi	Japan(Shiga)	-			1	1	2	1	4	2	1	2	1	1	1	2	1	1	1	2	1	2	2	2	1	1	2	1	1	1	1	2	1	2	1	4	1	1	3	2	1	1	2	1	1
288	しまひかり	Shimahikari	Japan(Hokkaido)	-			3	1	1	1	4	1	2	2	1	1	1	4	2	1	1	2	1	1	2	1	1	3	4	1	1	2	7	2	2	4	2	1	1	2	1	1	1	1	1	1	2
289	シモキタ	Shimokita	Japan(Aomori)	-			1	1	1	1	4	1	2	2	1	1	1	1	2	2	1	2	1	1	1	1	1	1	4	1	1	2	1	1	2	2	1	1	1	1	3	1	1	2	1	1	1
290	信交538号	Shinko 538	Japan(Nagano)	-	✓		3	2	1	2	1	1	1	1	1	1	1	4	2	1	1	1	1	1	1	2	1	4	9	2	2	2	1	1	2	4	1	1	1	1	3	1	1	2	1	1	2
291	シンレイ	Shinrei	Japan(Miyazaki)	-			1	1	1	1	4	1	1	1	1	1	1	1	1	1	1	4	1	1	1	1	1	1	1	1	1	1	3	2	2	1	1	1	1	1	3	1	1	2	1	1	1
292	神力	Shinriki	Japan(Hyogo)	JRC 33	✓		1	1	2	1	4	2	1	2	1	1	1	1	1	1	1	2	1	1	1	1	1	4	2	1	1	1	1	2	1	2	1	2	1	1	1	2	1	1	2	1	1
293	神力糯	Shinrikimochi	Japan(Kumamoto)	JRC 28			1	1	2	1	4	2	1	2	1	1	1	1	1	1	1	2	1	1	1	2	1	4	2	1	1	1	1	2	1	1	1	2	1	1	1	1	1	1	1	1	1
294	信州	Shinshuu	Japan(Nagano)	JRC 51	✓		1	1	1	1	4	1	1	1	1	1	1	2	1	1	1	2	1	1	2	1	1	1	5	1	1	1	3	2	2	1	1	4	1	1	1	1	1	2	1	1	1
295	新大正糯	Shintaishomochi	Japan(Toyama)	-	✓		1	2	1	2	4	1	1	1	1	1	1	1	1	1	1	1	1	1	1	1	1	1	1	1	1	1	1	2	1	1	1	4	1	1	3	1	1	1	1	2	1
296	新山吹	Shinyamabuki	Japan(Aichi)	-	✓		1	1	1	1	4	1	1	1	1	1	1	1	1	1	1	1	1	1	1	2	1	1	2	1	1	1	1	1	1	1	1	1	1	1	3	1	1	2	1	2	1
297	新山田穂2号	Shinyamadaho 2	Japan(Hyogo)	JRC 37			1	1	1	1	4	2	2	1	1	1	1	2	1	1	1	2	1	2	1	1	1	1	2	1	1	1	3	2	2	1	1	2	2	1	3	1	1	1	2	1	1
298	白稲（ケモミ）	Shiroine(Kemomi)	Japan(Tokushima)	JRC 39			1	1	1	1	4	2	1	2	1	1	1	2	1	1	1	2	1	1	2	1	1	1	1	1	1	1	1	1	1	1	2	4	1	1	3	1	1	1	2	1	1
299	庄内29号	Shonai 29	Japan(Yamagata)	-	✓		3	2	1	1	4	1	1	1	1	1	1	1	2	1	1	1	1	1	1	1	1	1	4	1	1	1	1	1	2	3	1	2	1	1	1	2	1	2	1	2	2
300	Silewah	Silewah	Indonesia	-	✓		1	1	2	2	4	1	2	2	2	1	1	4	1	1	1	2	1	1	2	2	1	1	9	2	2	2	1	1	2	5	2	2	2	1	1	1	2	1	1	1	1

(Continued)

Cultivar No.	Cultivar name (Kanji and Kana in Japanese)	Cultivar name (listed alphabetically)	Origin(Area)	WRC/JRC number[a]	Cultivars used in assay for primary screening	Alleles at 41 loci																																								
					No.[d]	-	1	-	2	-	-	3	-	-	-	-	-	4	5	-	-	-	6	-	-	-	-	-	-	-	7	-	8	-	-	-	-	-	-	9	-	-	10	-	11	12
					No.[c]	[1]	-	[2]	-	[3]	[4]	-	[5]	[6]	[7]	[8]	[9]	-	-	[10]	[11]	[12]	-	[13]	[14]	[15]	[16]	[17]	[18]	[19]	-	[20]	-	[21]	[22]	[23]	[24]	[25]	[26]	-	[27]	[28]	-	[29]	-	-
					No.[b]	1	2	3	4	5	6	7	8	9	10	11	12	13	14	15	16	17	18	19	20	21	22	23	24	25	26	27	28	29	30	31	32	33	34	35	36	37	38	39	40	41
					Locus name	OsGI	FA0057	LOC_Os01g62780	FA0118	OsMADS51	OsVIL2	FA2420	OsCOL4	DTH2	Ehd4	Ef-cd	OsMADS50	FA0192	FA0193	DTH3b	Hd6	Hd16	FA1067	OsHDT1	Hd17	RFT1	Hd3a	Hd1	Se5	OsFTIP1	FA0419	Ghd7	FA0444	OsMADS18	PRR37	Ehd3	Hd18	OsLHY	DTH8	FA2167-FA0494	Ehd2	Ehd1	FA0603	LOC_Os11g08410	FA1674	FA1759
301	そらち	Sorachi	Japan(Hokkaido)	-		3	1	1	2	4	1	2	1	2	1	1	4	1	1	1	1	3	2	2	2	1	3	4	1	1	2	7	2	2	4	2	1	1	2	3	1	1	2	1	1	2
302	水原258号	Suweon 258	Korea	-		5	1	4	2	3	1	2	2	3	1	1	1	1	1	2	2	2	1	2	1	1	4	9	3	2	2	4	1	3	5	1	1	1	1	3	1	2	1	1	1	1
303	タチアオバ	Tachiaoba	Japan(Fukuoka)	-		1	1	1	1	1	1	1	1	2	1	1	4	2	1	1	2	1	1	1	2	1	1	1	2	1	1	1	2	1	2	2	2	1	1	3	2	1	2	1	2	2
304	たちあやか	Tachiayaka	Japan(Hiroshima)	-		5	1	1	2	1	1	2	1	1	1	1	4	2	2	1	2	1	1	1	2	1	1	4	1	1	2	3	1	1	2	1	1	1	1	1	1	1	2	1	1	1
305	たちはるか	Tachiharuka	Japan(Fukuoka)	-		3	2	1	1	1	1	1	1	2	1	1	4	2	2	1	1	1	1	1	2	1	1	1	2	1	1	1	2	2	1	1	2	1	1	1	2	1	2	1	2	1
306	たちはやて	Tachihayate	Japan(Ibaraki)	-		5	1	1	2	1	1	1	2	1	1	1	4	2	2	2	2	2	1	1	1	1	1	4	3	1	2	3	1	3	5	1	1	1	1	1	1	2	2	1	1	2
307	たちじょうぶ	Tachijobu	Japan(Hokkaido)	-		1	1	1	1	4	1	2	1	1	1	1	4	2	1	2	1	1	1	2	2	1	3	2	1	1	2	6	1	1	4	1	1	1	1	3	1	1	2	1	1	1
308	たちすがた	Tachisugata	Japan(Ibaraki)	-		5	1	1	1	3	1	1	1	1	1	1	1	1	1	2	2	1	1	2	1	1	1	7	1	1	2	4	1	3	5	1	3	2	1	3	1	2	2	1	1	1
309	たちすずか	Tachisuzuka	Japan(Hiroshima)	-		5	1	1	2	4	1	1	1	1	1	1	4	2	2	1	1	1	1	1	2	1	1	2	1	1	2	1	2	1	2	1	1	1	1	1	1	1	2	1	1	1
310	Tadukan	Tadukan	Philippines	WRC 20		4	1	4	2	2	2	2	2	3	2	1	3	1	1	2	3	2	1	2	3	2	2	6	3	2	2	4	1	3	2	1	3	2	3	1	1	2	1	1	1	1
311	台中65号	Taichung 65	Taiwan	-	✓	1	1	2	1	4	1	1	2	1	1	1	1	1	1	1	2	1	1	2	2	1	1	5	1	1	1	1	2	2	2	1	2	2	1	1	1	3	1	2	1	1
312	台農67号	Tainung 67	Taiwan	-		1	1	2	1	4	1	2	2	2	1	1	1	1	1	1	2	1	1	2	1	1	4	5	3	2	1	1	2	1	2	1	2	2	1	3	1	3	1	1	1	1
313	タカナリ	Takanari	Japan(Ibaraki)	-	✓	5	1	4	2	3	1	2	2	3	1	1	1	1	1	2	2	2	1	2	1	1	1	7	3	2	2	4	1	3	5	1	3	2	1	3	1	2	1	1	1	1
314	たかやまもち	Takayamamochi	Japan(Gifu)	-		1	1	1	2	1	2	2	2	1	1	1	1	1	1	1	2	1	1	1	2	1	4	4	1	1	2	1	1	2	2	1	1	1	1	3	2	1	1	1	2	1
315	竹田早生	Takedawase	Japan(Okayama)	-	✓	1	1	2	2	4	1	1	2	1	1	1	2	1	1	1	2	1	1	1	1	1	4	2	1	1	1	1	1	1	2	1	4	1	1	3	1	1	1	2	1	1
316	竹成	Takenari	Japan(Mie)	-		1	1	2	2	4	2	1	2	1	1	1	1	1	1	1	2	1	1	1	1	1	1	2	1	1	1	3	2	2	1	1	4	1	1	3	1	1	1	1	1	1
317	Taporuri	Taporuri	Taiwan	-		4	1	3	2	3	1	2	2	3	2	1	3	1	1	2	3	2	1	2	3	1	2	9	4	2	2	2	1	3	7	1	3	2	3	1	1	2	1	1	1	1
318	たつこもち	Tatsukomochi	Japan(Akita)	-		1	1	1	1	4	1	1	2	1	1	1	2	1	1	1	2	1	1	2	1	1	4	4	1	2	2	1	1	2	1	1	4	1	1	3	2	1	1	1	2	2
319	てんこもり	Tenkomori	Japan(Toyama)	-		3	2	1	1	1	1	1	1	1	1	1	4	2	2	1	1	3	2	1	2	1	1	1	1	1	2	1	1	2	2	1	2	1	1	3	1	1	2	1	1	2
320	てんたかく	Tentakaku	Japan(Toyama)	-	✓	1	1	1	1	1	1	1	1	1	1	1	4	2	1	1	1	1	1	1	2	1	4	9	2	2	2	1	1	2	1	1	1	1	1	3	1	1	2	1	1	1
321	唐干	Touboshi	Japan(Kagoshima)	JRC 42	✓	4	1	4	2	1	2	2	2	3	2	1	3	1	1	2	3	1	1	2	3	2	2	3	4	2	2	4	1	3	9	1	3	2	8	1	1	2	1	1	1	1
322	とちぎの星	Tochiginohoshi	Japan(Tochigi)	-		3	2	1	2	1	1	1	1	1	1	1	4	1	1	1	1	3	2	1	2	1	1	1	1	2	1	1	1	2	2	1	2	1	1	3	1	1	1	1	2	1
323	トドロキワセ	Todorokiwase	Japan(Niigata)	-	✓	3	2	1	2	4	2	1	2	1	1	1	4	2	2	1	1	3	1	1	2	1	1	4	1	1	2	1	1	2	3	1	2	1	1	1	1	1	1	1	2	1
324	とがおとめ	Togaotome	Japan(Toyama)	-		3	2	1	2	1	1	1	1	1	1	1	4	2	1	1	1	3	1	1	2	1	1	4	1	1	1	1	1	2	1	1	1	1	1	3	1	1	2	1	2	2
325	東北206号	Tohoku 206	Japan(Miyagi)	-	✓	3	2	1	2	1	1	1	1	1	1	1	4	2	1	1	1	1	1	1	2	1	1	4	1	1	2	1	1	2	1	1	2	1	1	3	1	1	2	1	1	2
326	東北209号	Tohoku 209	Japan(Miyagi)	-	✓	3	2	1	2	1	1	1	1	1	1	1	4	2	1	1	1	1	1	1	2	1	1	4	1	1	2	1	1	2	1	1	2	1	1	3	1	1	2	1	2	2
327	東北210号	Tohoku 210	Japan(Miyagi)	-		1	1	1	2	1	1	2	1	1	1	1	4	2	2	1	1	1	1	2	2	1	1	4	1	1	2	1	1	2	1	1	2	1	1	3	1	1	2	1	2	1
328	東北228号	Tohoku 228	Japan(Miyagi)	-		3	2	1	2	1	1	1	1	1	1	1	4	2	1	1	1	1	1	1	2	1	1	4	1	1	2	1	1	2	1	1	2	1	1	3	1	1	2	1	2	2
329	ときめきもち	Tokimekimochi	Japna(Akita)	-		1	1	1	1	4	1	1	1	1	1	1	4	2	2	1	1	1	1	2	2	1	4	4	1	1	2	3	1	1	1	1	4	1	1	3	1	1	1	1	2	1
330	巴まさり	Tomoemasari	Japan(Hokkaido)	-	✓	3	1	1	2	1	1	1	2	1	1	1	1	1	1	1	1	3	2	1	2	1	3	4	1	1	2	7	2	2	1	1	4	2	1	3	1	1	2	1	1	2
331	ともほなみ	Tomohonami	Japan(Aichi)	-	✓	3	2	1	2	1	1	1	1	1	1	1	4	2	2	1	1	3	2	1	2	1	1	1	1	1	2	3	2	2	1	1	1	1	1	3	1	1	2	1	2	2
332	トヨハタモチ	Toyohatamochi	Japan(Ibaraki)	-		1	1	1	2	4	1	2	2	2	1	1	4	1	1	1	2	1	1	2	2	1	1	12	1	1	2	1	1	2	2	2	4	1	1	1	1	2	1	1	2	1
333	とよめき	Toyomeki	Japan(Ibaraki)	-		1	2	1	2	1	1	1	2	1	1	1	1	1	1	1	2	3	1	1	1	1	4	5	3	1	1	4	1	1	1	1	4	1	1	1	1	1	1	1	1	2
334	東山38号	Tozan 38	Japan(Gifu)	-	✓	1	1	1	1	4	1	1	1	1	1	1	1	1	1	1	1	1	1	1	2	1	1	2	1	1	1	1	2	1	2	1	1	1	1	1	1	1	2	1	2	1
335	つがるロマン	Tsugaruroman	Japan(Aomori)	-	✓	3	2	1	2	1	1	2	2	1	1	1	1	1	2	1	1	1	1	1	2	1	1	4	1	1	2	1	1	1	2	1	1	1	1	3	1	1	1	1	2	2
336	つきあかり	Tsukiakari	Japana(Niigata)	-		3	2	1	2	1	2	1	1	1	1	1	4	2	2	1	1	3	1	1	2	1	1	4	1	2	2	1	1	2	1	1	2	2	1	3	1	1	1	1	2	1
337	月の光	Tsukinohikari	Japan(Aichi)	-	✓	1	1	1	1	1	1	1	1	1	1	1	1	1	1	1	1	1	1	1	1	1	1	1	1	1	1	1	2	2	1	1	1	1	1	1	1	1	2	1	1	1
338	つきすずか	Tsukisuzuka	Japan(Hiroshima)	-		5	1	1	2	1	1	1	1	1	1	1	4	2	2	1	1	1	1	1	2	1	1	2	1	1	2	1	2	1	2	1	1	1	1	1	1	1	2	1	1	1
339	つくしろまん	Tsukushiroman	Japan(Fukuoka)	-		3	2	1	2	1	1	1	1	1	1	1	4	2	2	1	1	3	2	1	2	1	1	1	1	1	1	1	1	2	1	1	2	1	1	3	1	1	1	1	2	2
340	つくし早生	Tsukushiwase	Japan(Fukuoka)	-		3	2	1	2	4	1	1	1	1	1	1	1	1	1	1	1	1	2	1	2	1	1	1	1	1	1	1	1	2	1	1	1	1	1	1	1	1	2	1	2	1
341	つや姫	Tsuyahime	Japan(Yamagata)	-	✓	3	2	1	2	1	1	1	1	1	1	1	4	2	2	1	1	3	2	1	2	1	1	1	1	1	2	1	1	2	1	1	2	1	1	3	1	1	1	1	2	2
342	つやおとめ	Tsuyaotome	Japan(Fukuoka)	-	✓	1	1	1	2	1	1	1	1	1	1	1	4	2	2	1	1	1	2	1	2	1	1	1	1	1	1	1	2	2	1	1	2	1	1	3	1	1	2	1	1	2
343	ツユアケ	Tsuyuake	Japan(Nara)	-		1	1	1	1	4	1	1	2	1	1	1	1	1	1	1	1	1	1	1	1	1	1	2	1	1	1	1	1	1	1	1	2	2	1	3	2	1	1	1	2	1
344	Tupa 121-3	Tupa 121-3	Bangladesh	WRC 32	✓	4	1	4	2	3	2	2	2	3	2	1	3	1	1	2	3	2	1	2	3	1	2	9	2	2	2	1	1	2	5	1	3	2	9	1	1	2	1	1	1	1
345	浦三1号	Urasan 1	Japan(Tochigi)	WRC 51		4	1	2	2	4	1	2	2	2	1	1	4	1	1	1	2	1	1	2	2	1	1	11	2	1	2	1	1	2	2	1	4	1	1	1	1	2	1	1	2	1
346	早生関取F	Wasesekitori F	Japan(Unknown)	-		1	1	2	2	1	2	2	2	1	1	1	4	1	1	1	2	1	1	2	1	1	1	11	1	2	2	1	2	2	2	1	2	1	1	1	1	2	1	1	1	1
347	渡舟	Wataribune	Japan(Shiga)	JRC 19	✓	2	1	2	2	4	1	2	2	2	1	1	2	1	1	1	2	1	1	1	1	1	4	2	2	2	1	1	1	2	1	1	2	2	1	1	1	2	1	2	1	1
348	ヤマビコ	Yamabiko	Japan(Mie)	-	✓	1	1	1	1	4	2	1	2	1	1	1	1	1	1	1	1	1	1	1	1	1	4	2	1	1	1	1	1	1	1	1	1	1	1	3	1	1	2	1	1	1
349	山田化	Yamadabake	Japan(Kagoshima)	JRC 05		4	1	2	2	4	1	2	2	2	1	1	4	1	1	1	2	1	1	2	2	1	1	11	1	1	2	1	1	2	2	1	2	2	1	1	1	2	1	1	1	1
350	山田錦	Yamadanishiki	Japan(Hyogo)	-	✓	1	1	2	2	1	1	1	2	1	1	1	2	1	1	1	2	1	1	1	1	1	4	2	1	1	1	1	1	1	2	1	4	1	1	3	1	1	1	2	1	1

(Continued)

Cultivar No.	Cultivar name (Kanji and Kana in Japanese)	Cultivar name (listed alphabetically)	Origin(Area)	WRC/JRC number[a]	Cultivars used in assay for primary screening	Locus name	OsGI	FA0057	LOC_Os01g62780	FA0118	OsMADS51	OsVIL2	FA2420	OsCOL4	DTH2	Ehd4	Ef-cd	OsMADS50	FA0192	FA0193	DTH3b	Hd6	Hd16	FA1067	OsHDT1	Hd17	RFT1	Hd3a	Hd1	Se5	OsFTIP1	FA0419	Ghd7	FA0444	OsMADS18	PRR37	Ehd3	Hd18	OsLHY	DTH8	FA2167-FA0494	Ehd2	Ehd1	FA0603	LOC_Os11g08410	FA1674	FA1759
						No.[b]	1	2	3	4	5	6	7	8	9	10	11	12	13	14	15	16	17	18	19	20	21	22	23	24	25	26	27	28	29	30	31	32	33	34	35	36	37	38	39	40	41
						No.[c]	[1]	-	[2]	-	[3]	[4]	-	[5]	[6]	[7]	[8]	[9]	-	-	[10]	[11]	[12]	-	[13]	[14]	[15]	[16]	[17]	[18]	[19]	-	[20]	-	[21]	[22]	[23]	[24]	[25]	[26]	-	[27]	[28]	-	[29]	-	-
						No.[d]	-	1	-	2	-	-	3	-	-	-	-	-	4	5	-	-	-	6	-	-	-	-	-	-	-	7	-	8	-	-	-	-	-	-	9	-	-	10	-	11	12
351	やまだわら	Yamadawara	Japan(Ibaraki)	-			1	2	1	2	1	1	1	2	1	1	1	1	1	1	1	2	2	1	1	1	1	4	5	3	1	1	4	1	1	2	1	4	1	1	1	1	1	2	1	1	1
352	山形95号	Yamagata 95	Japan(Yamagata)	-	✓		3	2	1	2	1	1	1	1	2	1	1	4	2	2	1	1	1	1	1	2	1	1	4	1	1	2	1	1	2	2	1	2	1	1	3	2	1	2	1	2	2
353	山形糯128号	Yamagatamochi 128	Japan(Yamagata)	-			3	1	1	1	4	2	1	1	1	1	1	4	2	2	1	1	3	1	2	1	1	4	4	1	1	1	1	1	2	1	1	2	1	1	3	2	1	2	1	1	1
354	ヤマヒカリ	Yamahikari	Japan(Fukui)	-			1	2	1	1	1	1	1	1	1	1	1	4	2	2	1	1	1	2	1	1	1	1	1	1	1	1	1	2	2	1	1	1	1	1	3	1	1	2	1	1	2
355	やまのしずく	Yamanoshizuku	Japan(Miyagi)	-			3	2	1	2	1	1	1	1	1	1	1	4	2	2	1	1	1	1	1	2	1	1	4	1	1	2	1	1	2	3	1	1	1	1	3	1	1	1	1	2	1
356	ヤマセニシキ	Yamasenishiki	Japan(Akita)	-	✓		3	2	1	1	1	1	2	1	1	1	1	1	1	1	1	1	3	1	1	2	1	1	4	1	1	2	3	1	2	3	1	4	1	1	3	1	1	2	1	1	1
357	大和力	Yamatojikara	Japan(Chiba)	-	✓		1	1	1	2	4	2	2	2	1	1	1	4	2	1	1	4	1	1	2	1	1	1	4	1	2	2	1	1	1	3	1	2	1	1	3	1	1	1	1	1	1
358	ヤシロモチ	Yashiromochi	Japan(Shimane)	-			1	1	1	1	4	2	1	2	1	1	1	1	1	1	1	2	1	1	1	1	1	4	2	1	1	1	1	1	1	3	1	2	1	1	1	1	1	1	1	1	1
359	やたのもち	Yatanomochi	Japan(Ibaraki)	-			1	1	2	1	4	1	2	2	2	1	1	2	1	1	1	2	1	1	2	2	1	1	4	1	1	2	2	2	2	2	2	2	2	3	3	1	3	2	1	1	1
360	雪化粧	Yukigesho	Japan(Yamagata)	-			1	2	1	2	4	1	1	2	1	1	1	2	1	1	1	1	1	1	1	2	1	1	4	1	1	2	1	2	1	1	1	2	1	1	3	2	1	1	1	2	1
361	ゆきひかり	Yukihikari	Japan(Hokkaido)	-			3	1	1	2	4	1	1	1	1	1	1	1	1	1	1	1	1	1	2	1	1	3	1	1	1	2	6	2	2	4	1	1	1	1	1	1	1	2	1	2	1
362	雪姫羽二重糯	Yukihimehabutaemochi	Japan(Shiga)	-	✓		1	1	2	1	4	2	1	2	1	1	1	2	1	1	1	2	1	2	2	2	1	1	2	1	1	1	1	2	1	2	1	4	1	1	3	2	1	1	2	1	1
363	ゆきまる	Yukimaru	Japan(Hokkaido)	-			3	1	1	1	4	1	2	2	1	1	1	1	1	1	1	1	1	1	2	1	1	3	12	1	1	2	6	1	1	4	2	1	1	2	3	2	1	2	1	1	2
364	ゆきのはな	Yukinohana	Japan(Aomori)	-			3	1	1	1	4	1	1	1	1	1	1	2	1	1	1	1	3	1	2	1	1	3	12	1	1	2	6	2	2	2	2	2	1	1	3	2	1	2	1	2	2
365	ゆきの精	Yukinosei	Japan(Niigata)	-			1	2	1	1	4	2	2	2	1	1	1	4	2	2	1	1	1	2	1	2	1	1	4	1	1	1	1	1	1	2	1	4	1	1	1	1	1	2	1	1	1
366	ゆきさやか	Yukisayaka	Japan(Hokkaido)	-			3	1	1	2	4	1	2	1	2	1	1	2	1	1	1	1	1	1	2	1	1	3	2	1	1	2	6	1	1	4	1	1	1	1	3	2	1	2	1	1	1
367	雪若丸	Yukiwakamaru	Japan(Yamagata)	-			3	2	1	1	1	1	1	1	1	1	1	4	2	2	1	1	1	1	1	2	1	1	4	1	1	2	1	1	2	3	1	2	1	1	3	1	1	2	1	2	2
368	ゆめあかり	Yumeakari	Japan(Aomori)	-	✓		3	2	1	2	1	1	1	1	1	1	1	2	1	1	1	1	1	1	1	2	1	1	4	1	1	2	1	1	1	2	1	1	1	1	3	2	1	2	1	2	2
369	夢あおば	Yumeaoba	Japan(Niigata)	-			5	1	1	1	4	1	1	1	1	1	1	2	1	1	1	2	1	1	2	1	1	1	4	1	1	2	4	1	2	5	1	1	1	1	1	1	1	2	1	1	1
370	ゆめふわり	Yumefuwari	Japan(Akita)	-			1	2	1	2	1	1	1	1	1	1	1	4	1	2	1	1	1	1	2	2	1	1	4	1	1	2	3	1	2	1	1	1	1	1	3	1	1	1	1	2	1
371	ユメヒカリ	Yumehikari	Japan(Fukuoka)	-	✓		3	2	2	1	4	1	1	1	1	1	1	4	2	1	1	2	1	2	1	2	1	1	1	1	1	2	1	1	2	1	1	1	1	1	3	1	1	2	1	2	2
372	ゆめひたち	Yumehitachi	Japan(Ibaraki)	-	✓		3	2	1	2	1	1	1	1	1	1	1	4	1	1	1	1	3	2	1	2	1	1	1	1	1	2	1	1	2	1	1	1	1	1	1	1	1	1	1	2	1
373	ゆめのはたもち	Yumenohatamochi	Japan(Ibaraki)	-	✓		2	1	2	2	1	1	2	2	2	1	1	4	1	1	1	2	1	1	2	2	1	1	11	1	1	2	1	2	2	2	2	4	1	1	1	1	2	2	1	2	1
374	ゆめぴりか	Yumepirika	Japan(Hokkaido)	-	✓		3	1	1	2	4	1	2	2	1	1	1	1	2	1	1	1	1	1	2	1	1	3	2	1	1	2	6	1	1	4	2	1	1	1	3	1	1	2	1	1	2
375	夢十色	Yumetoiro	Japan(Niigata)	-			5	1	4	2	3	1	2	2	3	1	2	3	1	1	2	2	2	1	2	3	1	1	7	3	2	2	4	1	3	5	1	3	2	6	1	1	2	1	1	1	1
376	夢つくし	Yumetsukushi	Japan(Fukuoka)	-	✓		3	2	1	2	1	1	1	1	1	1	1	4	1	1	1	1	3	2	1	2	1	1	1	1	1	2	1	1	2	1	1	1	1	1	1	1	1	1	1	2	2
377	ゆみあずさ	Yumiazusa	Japan(Akita)	-			3	2	1	2	1	1	1	1	1	1	1	4	2	2	1	1	3	1	1	2	1	4	4	1	1	2	1	1	2	1	1	2	1	1	3	1	1	1	1	1	1

(Alleles at 41 loci)

* 'Moritawase' was represented by two different lines, labeled 'a' (#221) and 'b' (#222).

[a] Numbers in the Japanese Rice Core collection (JRC) and the World Rice Core collection (WRC) are given according to Kojima *et al.* (2005) and Ebana *et al.* (2008), respectively.

[b] Serial number based on the order of the physical positions of the 41 loci.

[c] Serial number of genes (Table 2, Fig. 1).

[d] Serial number of QTLs (Table 1, Fig. 1).

Supplemental Literature Cited

Kojima, Y., K. Ebana, S. Fukuoka, T. Nagamine and M. Kawase (2005) Development of an RFLP-based rice diversity research set of germplasm. Breed Sci 55: 431–440.

Supplemental Table 2. List of the 200 cultivars and their heading dates used in multiple linear regression analyses

Cultivar No.[a]	Cultivar name (Kanji and Kana in Japanese)	Cultivar name (listed alphabetically)	DTH[b]					
			April		May		June	
			Mean	SD	Mean	SD	Mean	SD
1	愛知旭	Aichiasahi	117.8	3.6	108.6	5.2	94.4	3.3
3	愛国	Aikoku	101.9	3.6	90.6	3.2	77.7	4.6
4	赤毛	Akage	71.9	5.5	64.3	6.3	59.0	5.2
5	赤米（長崎）	Akamai(Nagasaki)	129.0	3.9	116.3	4.7	99.6	3.2
6	赤米（徳島）	Akamai(Tokushima)	119.6	3.8	113.3	5.8	101.8	2.9
7	あかね空	Akanezora	105.8	3.5	91.6	2.8	77.8	5.0
8	アケボノ	Akebono	123.4	4.3	113.1	4.3	96.3	2.9
11	秋晴	Akibare	111.6	3.3	99.1	3.1	80.3	5.2
13	あきだわら	Akidawara	111.6	3.3	100.3	1.1	83.0	3.6
15	アキヒカリ	Akihikari	91.3	4.2	85.0	2.9	75.3	3.8
16	あきほ	Akiho	70.4	6.3	69.1	3.5	59.5	5.1
22	あきたこまち	Akitakomachi	93.5	4.4	84.2	3.6	73.4	4.5
23	アクネモチ	Akunemochi	124.7	3.8	115.7	4.5	100.4	3.2
25	葵の風	Aoinokaze	120.3	3.8	108.3	2.6	89.4	4.3
27	朝日	Asahi	120.6	4.3	111.0	4.3	96.0	4.0
28	あさひの夢	Asahinoyume	114.2	3.8	101.3	3.1	83.6	5.5
29	朝の光	Asanohikari	109.2	3.4	95.9	2.8	79.0	6.0
32	阿波赤米	Awaakamai	120.1	4.0	109.6	4.7	94.9	3.5
33	Basilanon	Basilanon	111.3	4.2	98.5	3.6	90.6	5.5
34	Bei Khe	Bei Khe	122.8	6.3	112.6	3.1	97.1	4.5
38	ベニセンゴク	Benisengoku	123.1	3.9	113.4	3.9	96.8	4.3
40	坊主	Bouzu	75.8	5.0	66.7	3.5	61.1	4.4
42	坊主糯	Bouzumochi	98.6	4.0	89.3	3.3	80.5	4.3
47	チヨホナミ	Chiyohonami	95.2	4.6	86.0	3.3	77.3	3.4
48	チヨニシキ	Chiyonishiki	98.4	4.6	90.0	2.9	80.6	4.2
49	中穣2号	Chujo 2	112.1	3.5	103.6	4.9	90.7	4.2
50	中京旭	Chukyoasahi	119.7	4.1	109.5	4.2	95.3	4.4
51	大地の風	Daichinokaze	120.6	3.7	108.7	4.1	90.9	7.1
52	団子	Dango	118.2	3.0	109.3	4.4	92.4	4.9
53	Davao 1	Davao 1	106.7	3.4	98.1	3.0	91.0	5.5
54	Deng Pao Zhai	Deng Pao Zhai	115.4	4.4	104.9	2.7	93.6	4.5
55	どまんなか	Domannaka	94.4	4.9	85.9	3.2	76.6	4.6
56	どんとこい	Dontokoi	104.8	4.0	90.4	2.8	76.0	5.2
60	フジヒカリ	Fujihikari	85.5	4.2	78.0	3.5	68.3	5.0
61	フジミノリ	Fujiminori	91.3	4.0	85.5	4.2	74.8	3.9
62	藤坂5号	Fujisaka 5	91.9	4.9	86.2	4.8	74.2	3.3
63	ふ系175号	Fukei 175	93.8	4.7	86.7	2.7	75.8	5.0
64	富国	Fukoku	70.1	7.2	59.0	6.2	53.0	4.8
65	ふくひびき	Fukuhibiki	95.5	5.0	86.6	3.9	75.6	3.9
70	ふさおとめ	Fusaotome	94.4	4.2	86.1	3.5	77.1	3.8
71	双葉	Futaba	114.5	3.7	104.2	4.1	87.6	3.8
72	凱旋糯	Gaisenmochi	113.4	3.7	105.0	4.4	88.3	3.5
75	銀坊主	Ginbouzu	114.2	4.2	103.4	3.6	84.4	5.7
78	五百万石	Gohyakumangoku	92.4	4.7	84.6	3.1	72.6	4.4
79	ゴロピカリ	Goropikari	111.5	3.3	99.2	2.7	82.2	5.0
81	ハバタキ	Habataki	100.0	4.4	90.9	3.8	82.7	4.5
82	はえぬき	Haenuki	97.2	4.8	87.6	3.5	77.4	5.1
86	ハナエチゼン	Hanaechizen	93.2	3.9	84.4	2.8	75.2	5.2
87	はなの舞	Hananomai	92.3	4.9	84.6	4.1	74.5	4.6
89	晴るる	Haruru	106.0	3.7	93.3	2.9	79.4	4.9
91	八束穂	Hassokuho	103.8	3.8	93.3	3.8	84.0	3.3
92	初星	Hatsuboshi	97.2	5.0	87.9	3.5	78.2	4.3
93	ハツニシキ	Hatsunishiki	92.1	4.1	83.4	4.3	75.8	4.6
94	ハツシモ	Hatsushimo	122.4	4.4	111.0	3.1	93.1	4.9
95	初雫	Hatsushizuku	73.5	7.1	69.9	3.5	63.1	5.1
96	はやまさり	Hayamasari	68.6	5.5	62.9	4.5	59.1	5.4
100	ヒメノモチ	Himenomochi	92.5	4.3	83.7	4.8	74.2	4.6
101	日の出	Hinode	95.9	4.6	87.5	3.3	78.0	4.5
102	ヒノヒカリ	Hinohikari	120.6	3.4	108.3	3.3	91.5	6.1
103	平山	Hirayama	96.4	4.0	88.8	3.2	79.3	4.7
105	ひとめぼれ	Hitomebore	98.8	4.4	90.9	2.9	81.8	5.2
106	冷立稲	Hiyadachito	89.3	4.4	79.4	3.3	67.5	5.2
107	ヒヨクモチ	Hiyokumochi	125.2	4.4	114.9	4.9	98.3	2.8
108	ほほほの穂	Hohohonoho	93.1	3.7	84.7	3.2	72.9	4.6
114	北陸193号	Hokuriku 193	109.4	4.0	104.8	3.3	94.8	5.0
115	宝満神社米	Homanjinjamai	132.1	3.8	118.3	4.7	100.0	4.2
116	ホウネンワセ	Hounenwase	92.5	4.2	85.2	3.9	76.3	4.2
121	ほしのゆめ	Hoshinoyume	74.2	4.1	68.7	3.2	62.3	4.5
123	細稈	Hosogara	88.7	3.9	81.5	4.2	70.6	5.2
124	ホウヨク	Houyoku	121.1	4.0	111.6	4.7	95.5	2.8

(Continued)

Cultivar No.[a]	Cultivar name (Kanji and Kana in Japanese)	Cultivar name (listed alphabetically)	DTH[b]					
			April		May		June	
			Mean	SD	Mean	SD	Mean	SD
132	IR 36	IR 36	111.1	3.9	105.1	3.4	95.0	5.2
133	IR 58	IR 58	105.9	4.3	98.6	3.1	90.4	6.6
135	入間錦	Irumanishiki	93.6	4.3	85.5	3.0	77.1	3.8
136	石白	Ishijiro	109.4	2.9	99.1	3.5	81.0	5.1
138	いただき	Itadaki	107.5	4.1	93.7	2.6	78.9	4.6
139	Italica Livorno	Italica Livorno	78.0	4.0	70.6	3.2	63.3	5.4
142	Jarjan	Jarjan	109.7	4.7	101.4	4.1	92.9	3.6
143	上州	Joshuu	105.1	3.8	90.3	2.4	75.0	6.0
144	十石	Jukkoku	114.4	3.4	104.2	3.5	88.3	4.6
145	かばしこ	Kabashiko	116.4	3.1	104.6	3.4	86.3	4.2
146	嘉平	Kahei	104.2	4.4	95.0	2.6	86.1	3.3
147	亀治	Kameji	119.1	4.2	108.3	4.5	91.4	4.7
148	亀の尾	Kamenoo	94.5	4.4	85.0	2.9	72.3	4.2
149	亀の尾4号	Kamenoo 4	93.7	4.5	85.6	3.7	77.2	4.3
150	金子b	Kaneko b	92.7	4.1	86.3	3.8	78.6	3.6
151	関東212号	Kanto 212	96.4	5.4	86.0	3.0	75.1	4.2
153	唐法師	Karahoshi	95.2	4.0	85.5	3.4	76.6	5.3
154	Kasalath	Kasalath	103.0	4.2	93.9	4.1	86.1	3.0
157	Khau Mac Kho	Khau Mac Kho	118.1	4.0	110.4	4.7	98.9	3.8
158	喜峰	Kihou	97.2	4.7	87.0	3.7	78.0	5.9
159	金南風	Kinmaze	112.0	4.0	103.4	3.7	90.8	2.8
161	キヌヒカリ	Kinuhikari	103.0	3.9	90.3	2.9	77.8	6.2
162	きぬむすめ	Kinumusume	114.6	3.7	103.1	3.3	84.6	4.6
164	きらら397	Kirara 397	75.5	3.2	69.7	3.0	62.6	4.2
165	キタアケ	Kitaake	73.0	3.9	67.4	2.9	61.4	4.6
168	キタヒカリ	Kitahikari	74.1	5.2	67.3	3.2	62.6	3.4
171	キヨニシキ	Kiyonishiki	93.2	4.8	83.6	5.6	73.1	5.0
173	黄金晴	Koganebare	113.1	3.1	99.4	3.4	81.4	5.7
174	コガネマサリ	Koganemasari	117.6	3.5	107.2	3.4	89.4	5.3
175	こがねもち	Koganemochi	100.4	3.8	88.6	3.3	76.9	4.6
180	コシヒカリ	Koshihikari	104.2	3.8	91.2	2.2	78.3	4.9
182	コシホマレ	Koshihomare	97.8	4.3	88.4	3.1	78.9	4.1
183	こしいぶき	Koshiibuki	98.2	4.5	88.8	2.9	79.1	3.5
184	越路早生	Koshijiwase	92.8	4.4	83.4	3.2	73.1	5.1
190	京都旭	Kyotoasahi	121.4	3.8	111.6	4.6	95.8	3.8
194	LTH	Lijiangxintuanheigu	96.3	4.3	91.3	1.7	81.1	3.8
196	まなむすめ	Manamusume	98.5	4.7	88.6	3.3	78.9	3.4
197	万作	Mansaku	97.9	3.4	88.4	2.9	77.4	3.4
199	祭り晴	Matsuribare	114.1	3.3	100.4	3.4	82.1	5.8
200	目黒糯	Meguromochi	110.6	4.0	102.3	3.2	87.0	4.5
201	めんこいな	Menkoina	96.6	4.3	87.9	3.9	78.3	3.7
206	密陽23号	Milyang 23	105.4	4.7	98.3	3.8	90.0	5.8
208	ミネアサヒ	Mineasahi	100.3	4.6	91.0	3.2	79.5	3.5
210	ミレニシキ	Mirenishiki	109.0	3.8	97.4	2.7	79.4	5.3
211	ミズハタモチ	Mizuhatamochi	94.4	4.7	84.9	3.3	74.1	4.8
212	ミズホチカラ	Mizuhochikara	113.2	3.3	108.8	4.4	99.5	1.1
221	森多早生	Moritawase_a	92.4	4.2	82.1	4.2	71.3	5.4
222	森田早生	Moritawase_b	92.1	4.4	82.8	3.5	71.0	4.7
223	Muha	Muha	103.4	3.6	98.1	4.0	89.7	4.7
224	むつほまれ	Mutsuhomare	90.5	4.5	83.0	4.2	71.9	4.8
225	Naba	Naba	125.1	4.2	119.0	4.1	107.4	2.1
226	名古屋白	Nagoyashiro	98.9	4.4	91.1	3.6	81.6	3.4
227	ナゴユタカ	Nagoyutaka	99.7	4.5	89.8	4.3	80.4	4.8
228	中生新千本	Nakateshinsenbon	111.8	3.8	103.8	3.9	90.1	3.2
229	ななつぼし	Nanatsuboshi	76.7	3.5	70.0	3.0	66.6	4.3
232	ナツヒカリ	Natsuhikari	91.0	4.2	82.4	3.5	71.7	4.4
235	ニホンマサリ	Nihonmasari	108.8	3.4	96.6	2.7	78.8	5.6
238	にこまる	Nikomaru	123.8	3.9	111.6	3.8	93.0	4.2
239	日本晴	Nipponbare	112.5	3.2	99.4	2.8	81.8	6.2
241	ニシホマレ	Nishihomare	120.8	3.7	111.6	4.3	94.6	3.3
243	農林１号	Nourin 1	91.6	3.9	84.0	3.7	75.3	3.8
245	農林18号	Nourin 18	127.4	4.0	116.7	3.6	99.4	2.7
246	農林22号	Nourin 22	111.8	3.9	101.8	3.5	85.7	5.5
247	農林29号	Nourin 29	117.2	3.7	107.0	4.5	88.6	5.0
248	農林６号	Nourin 6	113.4	3.4	103.9	4.2	87.0	4.1
249	農林8号	Nourin 8	116.4	3.2	106.3	3.7	88.8	5.7
250	能登ひかり	Notohikari	95.5	4.5	85.6	2.9	75.2	4.5
251	大場	Ouba	103.4	3.2	91.1	2.7	75.2	5.8
252	オオチカラ	Oochikara	99.6	5.7	92.0	2.7	81.9	2.5
254	おいらん	Oiran	102.9	4.2	91.9	2.8	83.3	4.0

(Continued)

Cultivar No.[a]	Cultivar name (Kanji and Kana in Japanese)	Cultivar name (listed alphabetically)	DTH[b]					
			April		May		June	
			Mean	SD	Mean	SD	Mean	SD
255	岡穂	Okabo	112.1	4.6	103.8	3.5	87.7	4.3
256	おきにいり	Okiniiri	96.5	4.8	86.9	3.3	76.3	5.4
257	オッカモドシ	Okkamodoshi	106.3	3.6	96.4	2.3	86.5	2.9
258	雄町	Omachi	124.9	4.8	112.3	3.5	94.1	4.1
261	オトメモチ	Otomemochi	92.0	4.2	84.2	3.2	77.6	6.0
262	奥羽197号	Ouu 197	97.3	4.4	89.9	3.4	79.9	2.8
263	奥羽292号	Ouu 292	94.0	4.9	85.0	3.2	74.4	4.7
264	オワリハタモチ	Owarihatamochi	95.3	4.1	89.4	3.6	80.3	4.5
267	Qiu Zhao Zong	Qiu Zhao Zong	85.4	3.9	75.6	3.1	65.7	4.0
268	雷電	Raiden	127.3	3.8	113.2	4.1	94.6	4.4
269	レイホウ	Reihou	119.7	4.4	109.7	3.1	95.6	3.3
270	レイメイ	Reimei	92.3	4.4	85.8	3.3	77.8	4.6
272	陸羽132号	Rikuu 132	94.2	4.8	87.2	3.5	76.9	3.8
273	陸羽20号	Rikuu 20	101.0	4.1	91.2	2.5	76.7	4.8
274	陸稲陸羽2号	Rikutorikuu 2	94.7	4.3	86.3	4.1	77.4	4.8
275	幸風	Sachikaze	120.6	3.4	107.9	3.1	91.0	6.0
276	さきひかり	Sakihikari	113.2	3.4	103.3	2.9	83.6	5.3
278	ササニシキ	Sasanishiki	95.1	4.1	85.4	3.4	75.2	4.9
279	さとじまん	Satojiman	110.6	3.3	96.9	2.9	82.2	5.2
280	関取	Sekitori	92.5	4.0	86.4	2.9	77.0	4.1
281	関山	Sekiyama	89.6	3.7	79.4	3.6	66.5	5.4
282	戦捷	Senshou	94.4	4.0	86.8	2.7	77.1	4.4
285	七面鳥糯	Shichimenchomochi	117.8	4.8	111.0	5.1	98.9	2.2
288	しまひかり	Shimahikari	77.4	5.2	69.1	3.9	60.3	5.0
292	神力	Shinriki	122.2	5.1	112.9	4.1	96.3	4.2
293	神力糯	Shinrikimochi	121.4	3.6	113.1	4.1	98.6	3.5
294	信州	Shinshuu	95.2	4.0	87.3	3.4	77.3	4.8
296	新山吹	Shinyamabuki	118.2	4.0	106.6	2.8	87.4	6.1
297	新山田穂2号	Shinyamadaho 2	115.4	3.9	109.4	3.6	90.7	3.5
298	白稲（ケモミ）	Shiroine(Kemomi)	114.9	3.9	106.1	4.5	88.6	5.0
299	庄内29号	Shonai 29	96.9	4.8	86.5	3.1	76.9	4.6
301	そらち	Sorachi	79.3	4.5	71.9	6.7	66.5	6.6
310	Tadukan	Tadukan	118.4	5.0	106.1	2.8	97.4	4.4
311	台中65号	Taichung 65	104.5	5.0	98.7	3.9	90.0	5.0
313	タカナリ	Takanari	104.7	3.9	98.4	4.2	90.0	5.1
316	竹成	Takenari	115.7	4.8	108.7	3.6	93.1	3.9
321	唐干	Touboshi	109.2	4.1	100.3	3.9	91.7	4.6
323	トドロキワセ	Todorokiwase	94.5	4.5	87.1	3.8	77.1	4.1
330	巴まさり	Tomoemasari	85.3	4.6	78.1	4.3	68.1	5.1
332	トヨハタモチ	Toyohatamochi	88.0	4.1	80.8	4.4	72.3	5.1
334	東山38号	Tozan 38	118.3	4.0	106.1	3.0	87.3	4.9
335	つがるロマン	Tsugaruroman	91.9	4.4	83.8	2.6	73.2	4.7
337	月の光	Tsukinohikari	113.3	3.5	99.6	2.8	81.2	6.2
341	つや姫	Tsuyahime	103.1	4.0	89.9	2.9	76.9	5.5
344	Tupa 121-3	Tupa 121-3	98.6	4.1	91.0	3.9	84.6	4.9
345	浦三1号	Urasan 1	94.8	4.6	84.1	2.7	75.1	4.4
346	早生関取F	Wasesekitori F	91.4	3.9	83.1	2.9	74.1	5.9
347	渡舟	Wataribune	118.3	3.6	109.3	3.5	94.0	3.7
348	ヤマビコ	Yamabiko	114.2	3.0	102.6	3.7	83.3	6.5
349	山田化	Yamadabake	103.7	4.5	93.3	3.5	83.4	5.2
350	山田錦	Yamadanishiki	120.0	4.0	110.7	3.8	94.1	3.7
356	ヤマセニシキ	Yamasenishiki	92.3	4.0	84.3	3.4	75.9	5.2
361	ゆきひかり	Yukihikari	68.1	5.5	62.6	4.4	55.2	4.4
363	ゆきまる	Yukimaru	68.6	5.4	65.0	4.5	58.2	4.2
365	ゆきの精	Yukinosei	99.5	3.9	88.3	3.2	78.8	3.3
368	ゆめあかり	Yumeakari	89.2	3.7	83.2	3.0	72.2	4.1
371	ユメヒカリ	Yumehikari	126.8	4.1	117.9	5.2	101.5	2.9
372	ゆめひたち	Yumehitachi	104.5	4.2	92.0	2.5	77.9	4.7
373	ゆめのはたもち	Yumenohatamochi	95.4	4.7	87.5	3.4	80.3	5.0
376	夢つくし	Yumetsukushi	105.2	3.7	91.2	2.7	77.7	4.9

[a] See Supplemental Table 1.

[b] Days-to-heading.

Supplemental Table 3. List of the 22 cultivars and their heading dates used in fitting a prediction model (model_April)

Cultivar No.[a]	Cultivar name (Kanji and Kana in Japanese)	Cultivar name (listed alphabetically)	DTH[b]		
			Cultivation years		
			2018	2019	2020
18	あきさかり	Akisakari	104	109	109
58	笑みの絆	Eminokizuna	100	108	106
118	ほしじるし	Hoshijirushi	106	114	112
128	イクヒカリ	Ikuhikari	98	106	107
141	泉348	Izumi 348	111	113	110
177	恋の予感	Koinoyokan	127	126	123
178	恋初めし	Koisomeshi	115	118	118
207	みなちから	Minachikara	100	110	112
213	みずほの輝き	Mizuhonokagayaki	107	112	113
217	萌えみのり	Moeminori	92	104	99
219	モミロマン	Momiroman	111	115	114
233	なつほのか	Natsuhonoka	106	113	113
283	せとのかがやき	Setonokagayaki	111	116	115
300	Silewah	Silewah	107	109	108
302	水原258号	Suweon 258	107	115	112
305	たちはるか	Tachiharuka	128	129	125
312	台農67号	Tainung 67	106	109	110
333	とよめき	Toyomeki	96	106	102
336	つきあかり	Tsukiakari	91	102	99
351	やまだわら	Yamadawara	105	112	107
354	ヤマヒカリ	Yamahikari	112	117	117
377	ゆみあずさ	Yumiazusa	93	104	102

[a] See Supplemental Table 1.

[b] Days-to-heading.

Supplemental Table 4. List of the previously identified functional mutations and of corresponding assays in this study

Gene name	Chr.[a]	Position_Start[b] (bp)	Position_End[b] (bp)	Mutation type[c] (size)	CDS[d] position	A.A. substitution[e]	Reference	Assay[f]	Representative cultivars[g]
LOC_Os01g62780	1	36,355,847	36,355,847	SNP [G>A]	328	V110I	Yano *et al.* (2016)	FA5414	Nishihomare, Yumehikari
OsMADS51	1	40,348,975	40,359,723	Deletion (10749 bp)	(Intron)	-	Chen *et al.* (2018)	FA6346	Takanari, Hokuriku 193
DTH2	2	30,096,330	30,096,330	SNP [A>G]	25	R9G	Wu *et al.* (2013)	FA5543[†]	IR 24, Hokuriku 193
	2	30,098,026	30,098,026	SNP [T>G]	955	Y319D	Wu *et al.* (2013)	FA5542	Hayamasari, Kitaake
Hd6	3	31,512,460	31,512,460	SNP [A>T]	271	Stop91K	Takahashi *et al.* (2001)	FA5408	Kasalath, Yumehikari
Hd16	3	33,001,571	33,001,571	SNP[G>C]	476	G159A	Kwon *et al.* (2014)	FA5723	Binxianludao[§], Fengrunhandaozi[§]
	3	33,002,789	33,002,789	SNP[G>A]	991	A331T	Hori *et al.* (2013)	FA5391	Koshihikari, Hanaechizen
Hd17	6	2,235,191	2,235,191	SNP [A>G]	1673	L558S	Matsubara *et al.* (2012)	FA5392	Koshihikari, Kasalath
RFT1	6	2,928,178	2,928,178	SNP [G>A]	313	E105K	Ogiso-Tanaka *et al.* (2013)	FA5490	Nona Bokra, Bei Khe
Hd3a	6	2,939,866	2,939,867	Insertion (4939 bp) in promoter region	(Upstream)	-	Kim *et al.* (2018)	FA6345	IR 36, Davao 1
Hd1	6	9,336,368	9,336,369	Insertion (4424 bp) in promoter region	(Upstream)	-	Fujino *et al.* (2009)<br>Goretti *et al.* (2017)	FA6347	Italica Livorno
	6	9,336,867	9,336,868	Insertion (36 bp)	333—334	P112ARRHQRVPVAPLP	Yano *et al.* (2000)	FA5656, Hd1-ID006[‡]<br>FA5499[†]	Ginbouzu, Hoshinoyume
	6	9,337,102	9,337,102	Deletion (1 bp)	568	Y191FS	Takahashi *et al.* (2009)	FA5748	Basilanon, Tadukan
	6	9,337,242	9,337,284	Deletion (43 bp)	708—750	P237FS	Yano *et al.* (2000)	FA5515, Hd1-ID004[‡]	Hitomebore, Sasanishiki
	6	9,338,005	9,338,006	Deletion (2 bp)	835—836	F279FS	Yano *et al.* (2000)	FA5508	Kasalath, Hanaechizen
	6	9,338,031	9,338,032	Insertion (1901 bp)	860—861	P288FS	Doi *et al.* (2004)	FA5746	Taichung 65, Tainung 67
	6	9,338,224	9,338,227	Deletion (4 bp)	1053—1056	K352FS	Takahashi *et al.* (2009)	FA5747	Davao 1, Takanari
	6	9,338,243	9,338,243	SNP [C>T]	1072	R358Stop	Takahashi *et al.* (2009)	FA5749	Khau Mac Kho
Ghd7	7	-	-	Locus deletion (null)	(Null)	(Null)	Xue *et al.* (2008)	"No Call" on all markers	Qiu Zhao Zong, Italica Livorno
	7	9,154,664	9,154,664	SNP [C>A]	157	E53Stop	Xue *et al.* (2008)	FA5530	Hoshinoyume, Hayamasari
	7	9,155,047	9,155,048	Insertion (1901 bp) in promoter region	(Upstream)	-	Asano *et al.* (2009)	FA6352, Ghd7-InDel_ProTn[‡]<br>FA5734[†]	Aikoku, Sorachi
PRR37	7	29,617,674	29,617,674	Deletion (1 bp)	245	R82FS	Yan *et al.* (2013)	FA5778	Toboushi, Kaneko b
	7	29,627,358	29,627,365	Deletion (8 bp)	1515—1522	K505FS	Koo *et al.* (2013)	FA5779	Davao 1, Qiu Zhao Zong
	7	29,628,481	29,628,481	SNP [T>C]	2110	Y704H	Koo *et al.* (2013)	FA5780	Bei Khe, Taporuri
	7	29,628,484	29,628,484	SNP [C>T]	2113	Q705Stop	Koo *et al.* (2013)	FA5781	Kasalath
	7	29,628,500	29,628,500	SNP [T>C]	2129	L710P	Koo *et al.* (2013)	FA5436	Kitaake, Hayamasari
Hd18	8	2,388,554	2,388,554	SNP [T>C]	673	T225A	Shibaya *et al.* (2016)	FA5400	Hayamasari, Kamenoo
DTH8	8	4,332,835	4,333,950	Deletion (1116 bp)	Missing since 790	G264FS	Yan *et al.* (2011)	Hd5_1116bpDel[‡],<br>FA5450[†], FA6349[†]	Bei Khe, Deng Pao Zhai
	8	4,334,417	4,334,417	Deletion (1 bp)	323	K108FS	Wei *et al.* (2010)	FA5777	IR 24, Habataki
	8	4,334,627	4,334,645	Deletion (19 bp)	95—113	A32FS	Wei *et al.* (2010)	FA5453[†], Hd5_19bpDel_1[‡]	Bouzu 6, Hayamasari
	8	4,335,990	4,335,990	SNP [A>C] in promoter region	(Upstream)	-	Wang *et al.* (2019)	FA6355	Kasalath, Tupa 121-3
Ehd1	10	17,077,589	17,077,589	SNP[C>T]	655	G219R	Doi *et al.* (2004)	FA5455	Taichung 65, Tainung 67

[a] Chromosome number.

[b] Position on the 'Nipponbare' IRGSP-1.0 reference genome (Kawahara *et al.* 2013). "-" at the *Ghd7* locus, the precise region of the mutation was not confirmed.

[c] Insertion and deletion sizes are relative to the 'Nipponbare' genome.

[d] Coding sequence.

[e] A.A., amino acid; FS, frameshift; Stop, stop codon. "-", change in transcriptional or translational activity caused by a large insertion, deletion, or SNP mutations.

[f] [†], Alternative assays developed to indirectly identify the mutation; [‡], insertion and deletion markers (see Supplemental Table 5 for details).

[g] [§], Cultivars found in the publicity available SNP database TASUKE+ (https://agrigenome.dna.affrc.go.jp/tasuke/ricegenomes/, Kumagai *et al.* 2019).

(**Continued**)

Supplemental Literature Cited

Asano, S., H. Saito, T. Asami, Y. Okumoto and T. Tanisaka (2009) A novel loss-of function allele at *E1* locus found in rice cultivars for Hokkaido. Breed Res 11 (Suppl. 2): 59 (in Japanese with English summary).

Chen, J.Y., H.W. Zhang, H.L. Zhang, J.Z. Ying, L.Y. Ma and J.Y. Zhuang (2018) Natural variation at *qHd1* affects heading date acceleration at high temperatures with pleiotropism for yield traits in rice. BMC Plant Biol 18: 112.

Fujino, K., Y. Matsuda, N. Iwata, Y. Nonoue and M. Yano (2009) Allelic variation of Hd1 gene controlling heading date in rice. Breed Res 11 (Suppl. 2): 60 (in Japanese with English summary).

Goretti, D., D. Martignago, M. Landini, V. Brambilla, J. Gómez-Ariza, N. Gnesutta, F. Galbiati, S. Collani, H. Takagi, R. Terauchi *et al.* (2017) Transcriptional and post-transcriptional mechanisms limit Heading date 1 (Hd1) function to adapt rice to high latitudes. PLoS Genet 13: e1006530.

Kim, S.R., G. Torollo, M.R. Yoon, J. Kwak, C.K. Lee, G.D. Prahalada, I.R. Choi, U.S. Yeo, O.Y. Jeong, K.K. Jena *et al.* (2018) Loss-of-function alleles of *heading date 1* (*Hd1*) are associated with adaptation of temperate *japonica* rice plants to the tropical region. Front Plant Sci 9: 1827.

Kwon, C.T., S.C. Yoo, B.H. Koo, S.H. Cho, J.W. Park, Z. Zhang, J. Li, Z. Li and N.C. Paek (2014) Natural variation in *Early flowering1* contributes to early flowering in *japonica* rice under long days. Plant Cell Environ 37: 101–112.

Takahashi, Y., K.M. Teshima, S. Yokoi, H. Innan and K. Shimamoto (2009) Variations in Hd1 proteins, *Hd3a* promoters, and *Ehd1* expression levels contribute to diversity of flowering time in cultivated rice. Proc Natl Acad Sci USA 106: 4555–4560.

Wang, P., Y. Xiong, R. Gong, Y. Yang, K. Fan and S. Yu (2019) A key variant in the cis-regulatory element of flowering gene *Ghd8* associated with cold tolerance in rice. Sci Rep 9: 9603.

Yan, W., H. Liu, X. Zhou, Q. Li, J. Zhang, L. Lu, T. Liu, C. Zhang, Z. Zhang, G. Shen *et al.* (2013) Natural variation in *Ghd7.1* plays an important role in grain yield and adaptation in rice. Cell Res 23: 969–971.

Yan, W.H., P. Wang, H.X. Chen, H.J. Zhou, Q.P. Li, C.R. Wang, Z.H. Ding, Y.S. Zhang, S.B. Yu, Y.Z. Xing *et al.* (2011) A major QTL, *Ghd8*, plays pleiotropic roles in regulating grain productivity, plant height, and heading date in rice. Mol Plant 4: 319–330.

Supplemental Table 5. Detailed information on the insertion and deletion (indel) markers used in this study

Marker name	Target gene[a]	Target mutation[a]	Primer sequence (5' to 3')		Chr.	Position of 5' end of forward and reverse primer[b]		Product size[c] (bp)
			Forward	Reverse		Start	End	
122500InDel-1[d]	*Ef-cd*	Deletion (36 bp)	TGGAGGTAGGTGGAAAAATGA	TGCATTGGAAGTATACAAAGAAAA	3	1,257,813	1,258,011	199/163
Hd1-ID006	*Hd1*	Insertion (36 bp)	GTGCGTGGCGTGCGACGT	CTCAGCGAGGACGGAGGTGGCC	6	9,336,825	9,336,906	82/118
Hd1-ID004	*Hd1*	Deletion (43 bp)	GCAGAGAAGGAAGGGAGCGAGTG	TCTGTGTAAGCACTGACGCCGG	6	9,337,213	9,337,346	134/91
Ghd7-InDel_ProTn	*Ghd7*	Insertion (1901 bp) in promoter region	GGAATCCATCATCATCGTGGCGA	GGCCAATGAGGAGTCGCCAAAT	7	9,154,724	9,155,177	454/2355
Hd5_1116bpDel	*DTH8*	Deletion (1116 bp)	CCCGCGTTGCTAGGGTTCGTTAT	GGGTGATGGAGTTCGAGGGCATC	8	4,332,502	4,333,973	1472/356
Hd5_19bpDel_1	*DTH8*	Deletion (19 bp)	GAACCTGTCCTGCTCCTTCG	CTTGCTGAGCCCGGTGG	8	4,334,554	4,334,713	160/141

Chr., chromosome number.

[a] See Supplemental Table 4 for further information on these mutations, except for *Ef-cd* .

[b] Position on the 'Nipponbare' IRGSP-1.0 reference genome (Kawahara *et al.* 2013).

[c] 'Nipponbare' allele / other allele.

[d] Marker developed by Fang *et al.* (2019).

Supplemental Table 6. Heading date–related genes targeted for the extraction of natural variations

Target locus name	No.	Chr.	Locus ID		No. of development sites	A set of 96-plex assays, HDA1[c]	Representative literature		
			RAP (Os)[a]	MSU (LOC_Os)[b]			Author (Year)	PMID	DOI
OsGI	1	1	Os01g0182600	LOC_Os01g08700.1	9	Yes (4)	Hayama *et al.* (2002)	12040096	10.1093/pcp/pcf059
RDD1	2	1	Os01g0264000	LOC_Os01g15900.1	1	-	Iwamoto *et al.* (2009)	19210638	10.1111/j.1365-3040.2009.01954.x
OsDof4	3	1	Os01g0277500	LOC_Os01g17000.1	2	-	Wu *et al.* (2017a)	29052517	10.1186/s12870-017-1109-0
OsSAMS3	4	1	Os01g0293000	LOC_Os01g18860.1	-	-	Li *et al.* (2011)	21757254	10.1016/j.jplph.2011.05.020
OsSAMS2	5	1	Os01g0323600	LOC_Os01g22010.1	-	-	Li *et al.* (2011)	21757254	10.1016/j.jplph.2011.05.020
OsELF3-2	6	1	Os01g0566100	LOC_Os01g38530.1	-	-	Murakami *et al.* (2007)	17132630	10.1093/pcp/pcl043
OsATG7	7	1	Os01g0614900	LOC_Os01g42850.1	-	-	Kurusu *et al.* (2014)	24674921	10.4161/auto.28279
Dlf1	8	1	Os01g0626400	LOC_Os01g43650.1	1	-	Cai *et al.* (2014)	25036785	10.1371/journal.pone.0102529
OsLFL1	9	1	Os01g0713600	LOC_Os01g51610.1	-	-	Peng *et al.* (2007)	17592727	10.1016/j.bbrc.2007.06.041
OsCCT01	10	1	Os01g0835700	LOC_Os01g61900.1	-	-	Zhang *et al.* (2015)	25563494	10.1038/srep07663
LOC_Os01g62780	11	1	Os01g0846450	LOC_Os01g62780.1	6	Yes (3)	Yano *et al.* (2016)	27322545	10.1038/ng.3596
OsABF1	12	1	Os01g0867300	LOC_Os01g64730.1	-	-	Hossain *et al.* (2010)	20039193	10.1007/s11103-009-9592-9
OsMADS51	13	1	Os01g0922800	LOC_Os01g69850.1	3	Yes (3)	Kim *et al.* (2007)	17951465	10.1104/pp.107.103291
OsHY2	14	1	Os01g0949400	LOC_Os01g72090.1	-	-	Saito *et al.* (2011)	20700573	10.1007/s00122-010-1426-2
OsLUX	15	1	Os01g0971800	LOC_Os01g74020.1	-	-	Murakami *et al.* (2007)	17132630	10.1093/pcp/pcl043
OsVIL2	16	2	Os02g0152500	LOC_Os02g05840.1	5	Yes (1)	Yang *et al.* (2013)	23083333	10.1111/tpj.12057
OsUbDKgamma4	17	2	Os02g0290500	LOC_Os02g18840.1	3	-	Song *et al.* (2017)	28254780	10.1105/tpc.16.00728
OsCTR2	18	2	Os02g0527600	LOC_Os02g32610.2	-	-	Wang *et al.* (2013)	24006427	10.1093/jxb/ert272
RCN2	19	2	Os02g0531600	LOC_Os02g32950.1	-	-	Nakagawa *et al.* (2002)	12148532	10.1046/j.1365-313X.2002.01255.x
Oscyt-inv1	20	2	Os02g0550600	LOC_Os02g34560.1	-	-	Jia *et al.* (2008)	18317796	10.1007/s00425-008-0718-0
OsCOL4	21	2	Os02g0610500	LOC_Os02g39710.1	3	Yes (1)	Lee *et al.* (2010)	20409004	10.1111/j.1365-313X.2010.04226.x
LTG1	22	2	Os02g0622100	LOC_Os02g40860.1	-	-	Lu *et al.* (2014)	24635058	10.1111/tpj.12487
OsCRY2	23	2	Os02g0625000	LOC_Os02g41550.1	-	-	Hirose *et al.* (2006)	16760221	10.1093/pcp/pcj064
DTH2	24	2	Os02g0724000	LOC_Os02g49230.1	8	Yes (2)	Wu *et al.* (2013)	23388640	10.1073/pnas.1213962110
OsBBX8	25	2	Os02g0731700	LOC_Os02g49880.1	-	-	Huang *et al.* (2012)	23118960	10.1371/journal.pone.0048242
PPS	26	2	Os02g0771100	LOC_Os02g53140.1	1	-	Tanaka *et al.* (2011)	21705640	10.1105/tpc.111.083436
OsGRF1	27	2	Os02g0776900	LOC_Os02g53690.1	-	-	Luo *et al.* (2005)	None	10.1111/j.1744-7909.2005.00071.x
HDR1	28	2	Os02g0793900	LOC_Os02g55080.1	-	-	Sun *et al.* (2016)	26954091	10.1371/journal.pgen.1005927
Ehd4	29	3	Os03g0112700	LOC_Os03g02160.1	6	Yes (1)	Gao *et al.* (2013)	23437005	10.1371/journal.pgen.1003281
Ef-cd	30	3	Os03g0122500	None	1	Yes (1)	Fang *et al.* (2019)	31451662	10.1073/pnas.1815030116
OsMADS50	31	3	Os03g0122600	LOC_Os03g03070.1<br>LOC_Os03g03100.1	9	Yes (3)	Lee *et al.* (2004)	15144377	10.1111/j.1365-313X.2004.02082.x
OsCCT10	32	3	Os03g0139500	LOC_Os03g04620.2	-	-	Zhang *et al.* (2015)	25563494	10.1038/srep07663
Se14	33	3	Os03g0151300+<br>Os03g0151400	LOC_Os03g05680.1+<br>LOC_Os03g05690.1	-	-	Yokoo *et al.* (2014)	24759811	10.1371/journal.pone.0096064
OsDof12	34	3	Os03g0169600	LOC_Os03g07360.1	-	-	Li *et al.* (2008)	18796165	10.1186/1471-2199-9-80
OsSUT1	35	3	Os03g0170900	LOC_Os03g07480.2	-	-	Ishimaru *et al.* (2001)	11673635	10.1093/pcp/pce148
OsMADS1	36	3	Os03g0215400	LOC_Os03g11614.1	-	-	Jeon *et al.* (2000)	10852934	10.1105/tpc.12.6.871
OsHAP5C	37	3	Os03g0251350	LOC_Os03g14669.1	-	-	Yang *et al.* (2017)	None	10.1016/j.cj.2016.06.014
OsPRR73	38	3	Os03g0284100	LOC_Os03g17570.4	-	-	Murakami *et al.* (2003)	14634161	10.1093/pcp/pcg135
DTH3b	39	3	Os03g0298800	LOC_Os03g18720.1	6	Yes (1)	Chen *et al.* (2015)	None	10.1007/s11032-015-0401-7
SDG718	40	3	Os03g0307800	LOC_Os03g19480.1	-	-	Liu *et al.* (2014)	25400654	10.3389/fpls.2014.00591
OsPhyB	41	3	Os03g0309200	LOC_Os03g19590.1	-	-	Takano *et al.* (2005)	16278346	10.1105/tpc.105.035899
Ospdhk1	42	3	Os03g0370000	LOC_Os03g25400.1	-	-	Mukherjee *et al.* (2012)	None	10.2135/cropsci2011.07.0352
OsPIPK1	43	3	Os03g0701800	LOC_Os03g49510.1	-	-	Ma *et al.* (2004)	15159629	10.1023/B:PLAN.0000028796.14336.24
OsCOL10	44	3	Os03g0711100	LOC_Os03g50310.1	-	-	Tan *et al.* (2016)	26872834	10.1093/pcp/pcw025
OsPhyA	45	3	Os03g0719800	LOC_Os03g51030.1	-	-	Takano *et al.* (2001)	11251094	10.1105/tpc.13.3.521
OsWDR5a	46	3	Os03g0725400	LOC_Os03g51550.1	-	-	Jiang *et al.* (2018a)	29440594	10.1104/pp.17.01749
OsPhyC	47	3	Os03g0752100	LOC_Os03g54084.1	2	-	Takano *et al.* (2005)	16278346	10.1105/tpc.105.035899
OsMADS14	48	3	Os03g0752800	LOC_Os03g54160.1	-	-	Lim *et al.* (2000)	11197326	10.1023/A:1026517111843
OsMADS34	49	3	Os03g0753100	LOC_Os03g54170.1	-	-	Lin *et al.* (2014)	24372518	10.1111/nph.12657
Hd6	50	3	Os03g0762000	LOC_Os03g55389.1	6	Yes (3)	Takahashi *et al.* (2001)	11416158	10.1073/pnas.111136798
Hd16	51	3	Os03g0793500	LOC_Os03g57940.1	6	Yes (3)	Hori *et al.* (2013)	23789941	10.1111/tpj.12268
SPIN1	52	3	Os03g0815700	LOC_Os03g60110.1	-	-	Vega-Sánchez *et al.* (2008)	18586868	10.1105/tpc.108.058610
OsIDS1	53	3	Os03g0818800	LOC_Os03g60430.1	-	-	Lee and An (2012)	22003982	10.1111/j.1365-313X.2011.04804.x

(**Continued**)

ETR2	54	4	Os04g0169100	LOC_Os04g08740.1	-	-	Wuriyanghan *et al.* (2009)	19417056	10.1105/tpc.108.065391
OsRR1	55	4	Os04g0442300	LOC_Os04g36070.1	-	-	Jain *et al.* (2006)	16472405	10.1186/1471-2229-6-1
RFL	56	4	Os04g0598300	LOC_Os04g51000.1	-	-	Rao *et al.* (2008)	18305171	10.1073/pnas.0709059105
HAF1	57	4	Os04g0648800	LOC_Os04g55510.1	-	-	Yang *et al.* (2015)	26296966	10.1105/tpc.15.00320
qHD5	58	5	Os05g0121600	LOC_Os05g03040.1	-	-	Sun *et al.* (2017)	27677631	10.1007/s00122-016-2787-y
OsSAMS1	59	5	Os05g0135700	LOC_Os05g04510.1	-	-	Li *et al.* (2011)	21757254	10.1016/j.jplph.2011.05.020
OsBBX14	60	5	Os05g0204600	LOC_Os05g11510.1	-	-	Bai *et al.* (2016)	27095397	10.1016/j.plantsci.2016.02.017
SERF1	61	5	Os05g0420300	LOC_Os05g34730.1	-	-	Schmidt *et al.* (2014)	24046061	10.1093/mp/sst131
OsEXP4	62	5	Os05g0477600	LOC_Os05g39990.1	-	-	Choi *et al.* (2003)	12782731	10.1105/tpc.011965
OsHAPL1	63	5	Os05g0494100	LOC_Os05g41450.1	-	-	Zhu *et al.* (2017)	28043949	10.1093/jxb/erw468
OsLF	64	5	Os05g0541400	LOC_Os05g46370.1	2	-	Zhao *et al.* (2011)	21549224	10.1016/j.nbt.2011.04.006
nl1	65	5	Os05g0578900	LOC_Os05g50270.1	-	-	Wang *et al.* (2009)	19337211	10.1038/cr.2009.36
NRRa	66	5	Os05g0595300	LOC_Os05g51690.1	-	-	Zhang *et al.* (2013)	23253602	10.1093/mp/sss157
OsHDT1	67	5	Os05g0597100	LOC_Os05g51830.1	1	Yes (1)	Li *et al.* (2011)	21760907	10.1371/journal.pone.0021789
Hd17	68	6	Os06g0142600	LOC_Os06g05060.1	6	Yes (2)	Matsubara *et al.* (2012)	22399582	10.1093/pcp/pcs028
RFT1	69	6	Os06g0157500	LOC_Os06g06300.1	8	Yes (1)	Ogiso-Tanaka *et al.* (2013)	24098411	10.1371/journal.pone.0075959
Hd3a	70	6	Os06g0157700	LOC_Os06g06320.1	9	Yes (4)	Kojima *et al.* (2002)	12407188	10.1093/pcp/pcf156
OsCOL16	71	6	Os06g0264200	LOC_Os06g15330.1	-	-	Wu *et al.* (2017b)	28554475	10.1016/j.plantsci.2017.04.004
Hd1	72	6	Os06g0275000	LOC_Os06g16370.1	29	Yes (13)	Yano *et al.* (2000)	11148291	10.1105/tpc.12.12.2473
SDG711	73	6	Os06g0275500	LOC_Os06g16390.1	-	-	Liu *et al.* (2014)	25400654	10.3389/fpls.2014.00591
Se5	74	6	Os06g0603000	LOC_Os06g40080.1	8	Yes (3)	Izawa *et al.* (2000)	10849355	10.1046/j.1365-313X.2000.00753.x
OsFTIP1	75	6	Os06g0614000	LOC_Os06g41090.1	1	Yes (1)	Song *et al.* (2017)	28254780	10.1105/tpc.16.00728
OsMADS15	76	7	Os07g0108900	LOC_Os07g01820.2	-	-	Wang *et al.* (2010)	20107517	10.1371/journal.pgen.1000818
SNB	77	7	Os07g0235800	LOC_Os07g13170.1	-	-	Lee and An (2012)	22003982	10.1111/j.1365-313X.2011.04804.x
Ghd7	78	7	Os07g0261200	LOC_Os07g15770.1	18	Yes (7)	Xue *et al.* (2008)	18454147	10.1038/ng.143
OsMADS18	79	7	Os07g0605200	LOC_Os07g41370.1	3	Yes (2)	Fornara *et al.* (2004)	15299121	10.1104/pp.104.045039
OsCOL13	80	7	Os07g0667300	LOC_Os07g47140.1	-	-	Sheng *et al.* (2016)	27405463	10.1007/s11103-016-0506-3
PRR37	81	7	Os07g0695100	LOC_Os07g49460.1	12	Yes (8)	Koo *et al.* (2013)	23713079	10.1093/mp/sst088
Ehd3	82	8	Os08g0105000	LOC_Os08g01420.1	1	Yes (1)	Matsubara *et al.* (2011)	21284756	10.1111/j.1365-313X.2011.04517.x
Hd18	83	8	Os08g0143400	LOC_Os08g04780.1	6	Yes (3)	Shibaya *et al.* (2016)	27318280	10.1093/pcp/pcw105
OsLHY	84	8	Os08g0157600	LOC_Os08g06110.2	1	Yes (1)	Ogiso *et al.* (2010)	20007447	10.1104/pp.109.148908
DTH8	85	8	Os08g0174500	LOC_Os08g07740.1	16	Yes (6)	Wei *et al.* (2010)	20566706	10.1104/pp.110.156943
GF14c	86	8	Os08g0430500	LOC_Os08g33370.1	-	-	Purwestri *et al.* (2009)	19179350	10.1093/pcp/pcp012
OsK4	87	8	Os08g0484600	LOC_Os08g37800.1	-	-	Sun *et al.* (2016)	26954091	10.1371/journal.pgen.1005927
OsCOL15	88	8	Os08g0536300	LOC_Os08g42440.1	-	-	Wu *et al.* (2018)	29154991	10.1016/j.bbrc.2017.11.095
OsNTL5	89	8	Os08g0562200	LOC_Os08g44820.1	-	-	Guo *et al.* (2018)	29774039	10.3389/fpls.2018.00555
OsFCA	90	9	Os09g0123200	LOC_Os09g03610.1	1	-	Lee *et al.* (2005)	16240176	10.1007/s11103-005-8105-8
OsTrx1	91	9	Os09g0134500	LOC_Os09g04890.1	1	-	Choi *et al.* (2014)	24420930	10.1104/pp.113.228049
OsCO3	92	9	Os09g0240200	LOC_Os09g06464.1	-	-	Kim *et al.* (2008)	18449564	10.1007/s00425-008-0742-0
OsEMF2b	93	9	Os09g0306800	LOC_Os09g13630.1	-	-	Xie *et al.* (2015)	None	10.1007/s11105-014-0733-1
SDG724	94	9	Os09g0307800	LOC_Os09g13740.1	-	-	Sun *et al.* (2012)	22892321	10.1105/tpc.112.101436
OsRRMh	95	9	Os09g0516300	LOC_Os09g34070.1	1	-	Liu and Cai (2013)	23621499	10.1111/jipb.12056
OsFD1	96	9	Os09g0540800	LOC_Os09g36910.1	-	-	Tsuji *et al.* (2013)	23324168	10.1093/pcp/pct005
SIP1	97	9	Os09g0560900	LOC_Os09g38790.3	-	-	Jiang *et al.* (2018b)	29611871	10.1111/nph.15122
Ehd2	98	10	Os10g0419200	LOC_Os10g28330.1	2	Yes (1)	Matsubara *et al.* (2008)	18790997	10.1104/pp.108.125542
Ehd1	99	10	Os10g0463400	LOC_Os10g32600.1	6	Yes (2)	Doi *et al.* (2004)	15078816	10.1101/gad.1189604
OsMADS56	100	10	Os10g0536100	LOC_Os10g39130.1	-	-	Ryu *et al.* (2009)	19558411	10.1111/j.1365-3040.2009.02008.x
JMJ706	101	10	Os10g0577600	LOC_Os10g42690.1	-	-	Sun and Zhou (2008)	18765808	10.1073/pnas.0805901105
RCN1	102	11	Os11g0152500	LOC_Os11g05470.1	-	-	Nakagawa *et al.* (2002)	12148532	10.1046/j.1365-313X.2002.01255.x
LOC_Os11g08410	103	11	Os11g0187200	LOC_Os11g08410.1	7	Yes (1)	Yano *et al.* (2016)	27322545	10.1038/ng.3596
OsFKF1	104	11	Os11g0547000	LOC_Os11g34460.1	-	-	Han *et al.* (2015)	25850808	10.1111/pce.12549
OsCCT41	105	12	Os12g0262400	LOC_Os12g16160.1	1	-	Zhang *et al.* (2015)	25563494	10.1038/srep07663
OsVIL1	106	12	Os12g0533500	LOC_Os12g34850.1	-	-	Jeong *et al.* (2016)	26795142	10.1007/s00299-015-1931-5
spl11	107	12	Os12g0570000	LOC_Os12g38210.1	-	-	Vega-Sánchez *et al.* (2008)	18586868	10.1105/tpc.108.058610
					218	(83)			

Chr., chromosome number; PMID, PubMed ID; DOI, Digital Object Identifier.

[a] Rice Annotation Project (http://rapdb.dna.affrc.go.jp/, Sakai *et al.* 2013).

[b] Michigan State University Rice Genome Annotation Project (http://rice.uga.edu/).

[c] "Yes" indicates inclusion in a set of the final 96-plex assays, HDA1. The number in parentheses is the number of assays.

(**Continued**)

Supplemental Literature Cited

Bai, B., J. Zhao, Y. Li, F. Zhang, J. Zhou, F. Chen and X. Xie (2016) *OsBBX14* delays heading date by repressing florigen gene expression under long and short-day conditions in rice. Plant Sci 247: 25–34.

Cai, Y., X. Chen, K. Xie, Q. Xing, Y. Wu, J. Li, C. Du, Z. Sun and Z. Guo (2014) Dlf1, a WRKY transcription factor, is involved in the control of flowering time and plant height in rice. PLoS One 9: e102529.

Choi, D., Y. Lee, H.T. Cho and H. Kende (2003) Regulation of expansin gene expression affects growth and development in transgenic rice plants. Plant Cell 15: 1386–1398.

Choi, S.C., S. Lee, S.R. Kim, Y.S. Lee, C. Liu, X. Cao and G. An (2014) Trithorax group protein *Oryza sativa* Trithorax1 controls flowering time in rice via interaction with early heading date3. Plant Physiol 164: 1326–1337.

Guo, S., S. Dai, P.K. Singh, H. Wang, Y. Wang, J.L.H. Tan, W. Wee and T. Ito (2018) A membrane-bound NAC-like transcription factor OsNTL5 represses the flowering in *Oryza sativa* . Front Plant Sci 9: 555.

Han, S.H., S.C. Yoo, B.D. Lee, G. An and N.C. Paek (2015) Rice FLAVIN-BINDING, KELCH REPEAT, F-BOX 1 (OsFKF1) promotes flowering independent of photoperiod. Plant Cell Environ 38: 2527–2540.

Hirose, F., T. Shinomura, T. Tanabata, H. Shimada and M. Takano (2006) Involvement of rice cryptochromes in de-etiolation responses and flowering. Plant Cell Physiol 47: 915–925.

Hossain, A., Y. Lee, J.-I. Cho, C.-H. Ahn, S.-K. Lee, J.-S. Jeon, H. Kang, C.-H. Lee, G. An and P.B. Park (2010) The bZIP transcription factor OsABF1 is an ABA responsive element binding factor that enhances abiotic stress signaling in rice. Plant Mol Biol 72: 557–566.

Huang, J., X. Zhao, X. Weng, L. Wang and W. Xie (2012) The rice B-box zinc finger gene family: genomic identification, characterization, expression profiling and diurnal analysis. PLoS One 7: e48242.

Ishimaru, K., T. Hirose, N. Aoki, S. Takahashi, K. Ono, S. Yamamoto, J. Wu, S. Saji, T. Baba, M. Ugaki *et al.* (2001) Antisense expression of a rice sucrose transporter OsSUT1 in rice (*Oryza sativa* L.). Plant Cell Physiol 42: 1181–1185.

Iwamoto, M., K. Higo and M. Takano (2009) Circadian clock- and phytochrome-regulated Dof-like gene, *Rdd1* , is associated with grain size in rice. Plant Cell Environ 32: 592–603.

Jain, M., A.K. Tyagi and J.P. Khurana (2006) Molecular characterization and differential expression of cytokinin-responsive type-A response regulators in rice (*Oryza sativa*). BMC Plant Biol 6: 1.

Jeon, J.S., S. Jang, S. Lee, J. Nam, C. Kim, S.H. Lee, Y.Y. Chung, S.R. Kim, Y.H. Lee, Y.G. Cho *et al.* (2000) *leafy hull sterile1* is a homeotic mutation in a rice MADS box gene affecting rice flower development. Plant Cell 12: 871–884.

Jeong, H.J., J. Yang, L.H. Cho and G. An (2016) OsVIL1 controls flowering time in rice by suppressing *OsLF* under short days and by inducing *Ghd7* under long days. Plant Cell Rep 35: 905–920.

Jia, L., B. Zhang, C. Mao, J. Li, Y. Wu, P. Wu and Z. Wu (2008) OsCYT-INV1 for alkaline/neutral invertase is involved in root cell development and reproductivity in rice (*Oryza sativa* L.). Planta 228: 51–59.

Jiang, P., S. Wang, H. Jiang, B. Cheng, K. Wu and Y. Ding (2018a) The COMPASS-like complex promotes flowering and panicle branching in rice. Plant Physiol 176: 2761–2771.

Jiang, P., S. Wang, H. Zheng, H. Li, F. Zhang, Y. Su, Z. Xu, H. Lin, Q. Qian and Y. Ding (2018b) SIP1 participates in regulation of flowering time in rice by recruiting OsTrx1 to *Ehd1* . New Phytol 219: 422–435.

Kim, S.K., C.H. Yun, J.H. Lee, Y.H. Jang, H.Y. Park and J.K. Kim (2008) *OsCO3* , a *CONSTANS-LIKE* gene, controls flowering by negatively regulating the expression of *FT* -like genes under SD conditions in rice. Planta 228: 355–365.

Kurusu, T., T. Koyano, S. Hanamata, T. Kubo, Y. Noguchi, C. Yagi, N. Nagata, T. Yamamoto, T. Ohnishi, Y. Okazaki *et al.* (2014) OsATG7 is required for autophagy-dependent lipid metabolism in rice postmeiotic anther development. Autophagy 10: 878–888.

Lee, D.Y. and G. An (2012) Two AP2 family genes, *SUPERNUMERARY BRACT* (*SNB*) and *OsINDETERMINATE SPIKELET 1* (*OsIDS1*), synergistically control inflorescence architecture and floral meristem establishment in rice. Plant J 69: 445–461.

Lee, J.H., Y.S. Cho, H.S. Yoon, M.C. Suh, J. Moon, I. Lee, D. Weigel, C.H. Yun and J.K. Kim (2005) Conservation and divergence of FCA function between *Arabidopsis* and rice. Plant Mol Biol 58: 823–838.

Li, D., C. Yang, X. Li, G. Ji and L. Zhu (2008) Sense and antisense *OsDof12* transcripts in rice. BMC Mol Biol 9: 80.

Li, W., Y. Han, F. Tao and K. Chong (2011) Knockdown of SAMS genes encoding S-adenosyl-l-methionine synthetases causes methylation alterations of DNAs and histones and leads to late flowering in rice. J Plant Physiol 168: 1837–1843.

Lim, J., Y.H. Moon, G. An and S.K. Jang (2000) Two rice MADS domain proteins interact with *OsMADS1* . Plant Mol Biol 44: 513–527.

Lin, X., F. Wu, X. Du, X. Shi, Y. Liu, S. Liu, Y. Hu, G. Theißen and Z. Meng (2014) The pleiotropic *SEPALLATA* -like gene *OsMADS34* reveals that the 'empty glumes' of rice (*Oryza sativa*) spikelets are in fact rudimentary lemmas. New Phytol 202: 689–702.

Liu, D. and X. Cai (2013) *OsRRMh* , a *Spen* -like gene, plays an important role during the vegetative to reproductive transition in rice. J Integr Plant Biol 55: 876–887.

Liu, X., C. Zhou, Y. Zhao, S. Zhou, W. Wang and D.X. Zhou (2014) The rice enhancer of zeste [E(z)] genes *SDG711* and *SDG718* are respectively involved in long day and short day signaling to mediate the accurate photoperiod control of flowering time. Front Plant Sci 5: 591.

Lu, G., F.Q. Wu, W. Wu, H.J. Wang, X.M. Zheng, Y. Zhang, X. Chen, K. Zhou, M. Jin, Z. Cheng *et al.* (2014) Rice *LTG1* is involved in adaptive growth and fitness under low ambient temperature. Plant J 78: 468–480.

Luo, A.D., L. Liu, Z.S. Tang, X.Q. Bai, S.Y. Cao and C.C. Chu (2005) Down-regulation of *OsGRF1* gene in rice *rhd1* mutant results in reduced heading date. J Integr Plant Biol 47: 745–752.

Ma, H., S.P. Xu, D. Luo, Z.H. Xu and H.W. Xue (2004) *OsPIPK1* , a rice phosphatidylinositol monophosphate kinase, regulates rice heading by modifying the expression of floral induction genes. Plant Mol Biol 54: 295–310.

Mukherjee, R., S. Gayen, A. Chakraborty, J. Bhattacharyya, M.K. Maiti, A. Basu and S.K. Sen (2012) Double-stranded RNA-mediated downregulation of *pdhk* gene expression to shorten maturation time of a late maturing native *indica* rice cultivar, Badshahbhog. Crop Sci 52: 1743–1753.

Murakami, M., M. Ashikari, K. Miura, T. Yamashino and T. Mizuno (2003) The evolutionarily conserved *Os* PRR quintet: rice pseudo-response regulators implicated in circadian rhythm. Plant Cell Physiol 44: 1229–1236.

Murakami, M., Y. Tago, T. Yamashino and T. Mizuno (2007) Comparative overviews of clock-associated genes of *Arabidopsis thaliana* and *Oryza sativa* . Plant Cell Physiol 48: 110–121.

Nakagawa, M., K. Shimamoto and J. Kyozuka (2002) Overexpression of *RCN1* and *RCN2* , rice *TERMINAL FLOWER 1/CENTRORADIALIS* homologs, confers delay of phase transition and altered panicle morphology in rice. Plant J 29: 743–750.

Peng, L.T., Z.Y. Shi, L. Li, G.Z. Shen and J.L. Zhang (2007) Ectopic expression of OsLFL1 in rice represses *Ehd1* by binding on its promoter. Biochem Biophys Res Commun 360: 251–256.

Purwestri, Y.A., Y. Ogaki, S. Tamaki, H. Tsuji and K. Shimamoto (2009) The 14-3-3 protein GF14c acts as a negative regulator of flowering in rice by interacting with the florigen Hd3a. Plant Cell Physiol 50: 429–438.

Rao, N.N., K. Prasad, P.R. Kumar and U. Vijayraghavan (2008) Distinct regulatory role for *RFL* , the rice *LFY* homolog, in determining flowering time and plant architecture. Proc Natl Acad Sci USA 105: 3646–3651.

Ryu, C.H., S. Lee, L.H. Cho, S.L. Kim, Y.S. Lee, S.C. Choi, H.J. Jeong, J. Yi, S.J. Park, C.D. Han *et al.* (2009) *OsMADS50* and *OsMADS56* function antagonistically in regulating long day (LD)-dependent flowering in rice. Plant Cell Environ 32: 1412–1427.

Saito, H., Y. Okumoto, Y. Yoshitake, H. Inoue, Q. Yuan, M. Teraishi, T. Tsukiyama, H. Nishida and T. Tanisaka (2011) Complete loss of photoperiodic response in the rice mutant line X61 is caused by deficiency of phytochrome chromophore biosynthesis gene. Theor Appl Genet 122: 109–118.

Schmidt, R., J.H. Schippers, D. Mieulet, M. Watanabe, R. Hoefgen, E. Guiderdoni and B. Mueller-Roeber (2014) SALT-RESPONSIVE ERF1 is a negative regulator of grain filling and gibberellin-mediated seedling establishment in rice. Mol Plant 7: 404–421.

(**Continued**)

Sheng, P., F. Wu, J. Tan, H. Zhang, W. Ma, L. Chen, J. Wang, S. Zhu, X. Guo, X. Zhang *et al.* (2016) A *CONSTANS-like* transcriptional activator, *OsCOL13*, functions as a negative regulator of flowering downstream of *OsphyB* and upstream of *Ehd1* in rice. Plant Mol Biol 92: 209–222.

Sun, B., X.D. Zhan, Z.C. Lin, W.X. Wu, P. Yu, Y.X. Zhang, L.P. Sun, L.Y. Cao and S.H. Cheng (2017) Fine mapping and candidate gene analysis of *qHD5*, a novel major QTL with pleiotropism for yield-related traits in rice (*Oryza sativa* L.). Theor Appl Genet 130: 247–258.

Sun, C., J. Fang, T. Zhao, B. Xu, F. Zhang, L. Liu, J. Tang, G. Zhang, X. Deng, F. Chen *et al.* (2012) The histone methyltransferase SDG724 mediates H3K36me2/3 deposition at *MADS50* and *RFT1* and promotes flowering in rice. Plant Cell 24: 3235–3247.

Sun, Q. and D.X. Zhou (2008) Rice jmjC domain-containing gene *JMJ706* encodes H3K9 demethylase required for floral organ development. Proc Natl Acad Sci USA 105: 13679–13684.

Sun, X., Z. Zhang, J. Wu, X. Cui, D. Feng, K. Wang, M. Xu, L. Zhou, X. Han, X. Gu *et al.* (2016) The *Oryza sativa* regulator HDR1 associates with the kinase OsK4 to control photoperiodic flowering. PLoS Genet 12: e1005927.

Takano, M., H. Kanegae, T. Shinomura, A. Miyao, H. Hirochika and M. Furuya (2001) Isolation and characterization of rice phytochrome A mutants. Plant Cell 13: 521–534.

Takano, M., N. Inagaki, X. Xie, N. Yuzurihara, F. Hihara, T. Ishizuka, M. Yano, M. Nishimura, A. Miyao, H. Hirochika *et al.* (2005) Distinct and cooperative functions of phytochromes A, B, and C in the control of deetiolation and flowering in rice. Plant Cell 17: 3311–3325.

Tan, J., M. Jin, J. Wang, F. Wu, P. Sheng, Z. Cheng, X. Zheng, L. Chen, M. Wang, S. Zhu *et al.* (2016) *OsCOL10*, a *CONSTANS-like* gene, functions as a flowering time repressor downstream of *Ghd7* in rice. Plant Cell Physiol 57: 798–812.

Tanaka, N., H. Itoh, N. Sentoku, M. Kojima, H. Sakakibara, T. Izawa, J. Itoh and Y. Nagato (2011) The *COP1* ortholog *PPS* regulates the juvenile-adult and vegetative-reproductive phase changes in rice. Plant Cell 23: 2143–2154.

Tsuji, H., H. Nakamura, K. Taoka and K. Shimamoto (2013) Functional diversification of FD transcription factors in rice, components of florigen activation complexes. Plant Cell Physiol 54: 385–397.

Vega-Sánchez, M.E., L. Zeng, S. Chen, H. Leung and G.L. Wang (2008) SPIN1, a K homology domain protein negatively regulated and ubiquitinated by the E3 ubiquitin ligase SPL11, is involved in flowering time control in rice. Plant Cell 20: 1456–1469.

Wang, K., D. Tang, L. Hong, W. Xu, J. Huang, M. Li, M. Gu, Y. Xue and Z. Cheng (2010) *DEP* and *AFO* regulate reproductive habit in rice. PLoS Genet 6: e1000818.

Wang, L., H. Yin, Q. Qian, J. Yang, C. Huang, X. Hu and D. Luo (2009) *NECK LEAF 1*, a GATA type transcription factor, modulates organogenesis by regulating the expression of multiple regulatory genes during reproductive development in rice. Cell Res 19: 598–611.

Wang, Q., W. Zhang, Z. Yin and C.K. Wen (2013) Rice CONSTITUTIVE TRIPLE-RESPONSE2 is involved in the ethylene-receptor signalling and regulation of various aspects of rice growth and development. J Exp Bot 64: 4863–4875.

Wu, Q., X. Liu, D. Yin, H. Yuan, Q. Xie, X. Zhao, X. Li, L. Zhu, S. Li and D. Li (2017a) Constitutive expression of *OsDof4*, encoding a C_2-C_2 zinc finger transcription factor, confesses its distinct flowering effects under long- and short-day photoperiods in rice (*Oryza sativa* L.). BMC Plant Biol 17: 166.

Wu, W., X.M. Zheng, D. Chen, Y. Zhang, W. Ma, H. Zhang, L. Sun, Z. Yang, C. Zhao, X. Zhan *et al.* (2017b) *OsCOL16*, encoding a CONSTANS-like protein, represses flowering by up-regulating *Ghd7* expression in rice. Plant Sci 260: 60–69.

Wu, W., Y. Zhang, M. Zhang, X. Zhan, X. Shen, P. Yu, D. Chen, Q. Liu, S. Sinumporn, K. Hussain *et al.* (2018) The rice CONSTANS-like protein OsCOL15 suppresses flowering by promoting *Ghd7* and repressing *RID1*. Biochem Biophys Res Commun 495: 1349–1355.

Wuriyanghan, H., B. Zhang, W.H. Cao, B. Ma, G. Lei, Y.F. Liu, W. Wei, H.J. Wu, L.J. Chen, H.W. Chen *et al.* (2009) The ethylene receptor ETR2 delays floral transition and affects starch accumulation in rice. Plant Cell 21: 1473–1494.

Xie, S., M. Chen, R. Pei, Y. Ouyang and J. Yao (2015) *OsEMF2b* acts as a regulator of flowering transition and floral organ identity by mediating H3K27me3 deposition at *OsLFL1* and *OsMADS4* in rice. Plant Mol Biol Report 33: 121–132.

Yang, W., Z. Lu, Y. Xiong and J. Yao (2017) Genome-wide identification and co-expression network analysis of the *OsNF-Y* gene family in rice. Crop J 5: 21–31.

Yang, Y., D. Fu, C. Zhu, Y. He, H. Zhang, T. Liu, X. Li and C. Wu (2015) The RING-finger ubiquitin ligase HAF1 mediates heading date 1 degradation during photoperiodic flowering in rice. Plant Cell 27: 2455–2468.

Yokoo, T., H. Saito, Y. Yoshitake, Q. Xu, T. Asami, T. Tsukiyama, M. Teraishi, Y. Okumoto and T. Tanisaka (2014) *Se14*, encoding a JmjC domain-containing protein, plays key roles in long-day suppression of rice flowering through the demethylation of H3K4me3 of *RFT1*. PLoS One 9: e96064.

Zhang, L., Q. Li, H. Dong, Q. He, L. Liang, C. Tan, Z. Han, W. Yao, G. Li, H. Zhao *et al.* (2015) Three CCT domain-containing genes were identified to regulate heading date by candidate gene-based association mapping and transformation in rice. Sci Rep 5: 7663.

Zhang, Y., G. Zhang, N. Xiao, L. Wang, Y. Fu, Z. Sun, R. Fang and X. Chen (2013) The rice *'nutrition response and root growth'* (*NRR*) gene regulates heading date. Mol Plant 6: 585–588.

Zhao, X.L., Z.Y. Shi, L.T. Peng, G.Z. Shen and J.L. Zhang (2011) An atypical HLH protein OsLF in rice regulates flowering time and interacts with OsPIL13 and OsPIL15. N Biotechnol 28: 788–797.

Zhu, Y.J., Y.Y. Fan, K. Wang, D.R. Huang, W.Z. Liu, J.Z. Ying and J.Y. Zhuang (2017) *Rice Flowering Locus T 1* plays an important role in heading date influencing yield traits in rice. Sci Rep 7: 4918.

Supplemental Table 7. Putative QTLs for days-to-heading identified using all RILs from a cross between 'Kanto 209' (K209) and 'Koshihikari Aichi SBL' (KoASBL).

Year	RILs	R/qtl function[a]	QTL name	LOD peak[b]			LOD thresholds at 5% significance level	Genetic effects[c]			Nearest marker[g]	Chr.	Position (cM)	LOD value	Physical position[h] (bp)	Related genes		This study
				Chr.	Position (cM)	LOD value		AE[d]	D[e]	PVE[f] (%)						Known gene	Reference	Gene or QTL serial
2011	F_4, F_5	CIM	*qDTH1-1*	1	39.2	3.05 †	4.49	-0.71	0.38	3.13	aa01005142	1	39.2	3.05	4,838,836	*OsGI*	Hayama *et al.* (2002)	QTL_01
			qDTH7-1	7	0.0	3.07 †		-0.69	0.18	2.79	aa07000615	7	0.0	3.07	2,651,062	*OsMADS15*	Wang *et al.* (2010)	QTL_07
			qDTH8-1	8	28.0	35.52		-3.30	-1.54	61.70	aa08000727	8	27.4	34.02	1,798,321	*Hd18*	Shibaya *et al.* (2016)	[24] *Hd18*
			qDTH8-2	8	88.0	3.60 †		-0.57	0.07	1.74	aa08004016	8	87.3	3.59	14,786,388	Unknown	Unknown	QTL_09
		MIM	*qDTH1-2*	1	642.0	-	-	-0.04	-0.17	9.42	aa01010816	1	642.0	-	37,507,621	*LOC_Os01g62780–OsMADS51*	Yano *et al.* (2016), Kim *et al.* (2007)	QTL_02
			qDTH7-1	7	0.0	-	-	-1.06	1.14	6.42	aa07000615	7	0.0	-	2,651,062	*OsMADS15*	Wang *et al.* (2010)	QTL_07
			qDTH8-1	8	26.0	-	-	-3.14	-0.87	72.68	aa08000727	8	27.4	-	1,798,321	*Hd18*	Shibaya *et al.* (2016)	[24] *Hd18*
			qDTH8-2	8	84.0	-	-	-0.65	-0.42	2.75	aa08002627	8	85.1	-	10,336,136	Unknown	Unknown	QTL_09
			qDTH1-2	1	642.0	-	-	3.70 (Both 'K209')	-	4.63	-	-	-	-	-	-	-	-
			qDTH8-1	8	26.0			-2.65 (Both 'KoASBL')										
			qDTH7-1	7	0.0	-	-	4.64 (Both 'K209')	-	3.07	-	-	-	-	-	-	-	-
			qDTH8-1	8	26.0			-3.76 (Both 'KoASBL')										
2012	F_5, F_6	CIM	*qDTH1-1*	1	18.0	4.02 †	4.40	-0.33	-0.90	1.75	aa01000889	1	15.1	3.59	1,842,582	*OsGI*	Hayama *et al.* (2002)	QTL_01
			qDTH1-2	1	643.6	4.75		-0.60	0.69	3.86	aa01010816	1	643.6	4.75	37,507,621	*LOC_Os01g62780–OsMADS51*	Yano *et al.* (2016), Kim *et al.* (2007)	QTL_02
			qDTH7-1	7	10.0	3.12 †		-0.43	-0.12	1.60	aa07001067	7	12.2	2.80	3,654,626	*OsMADS15*	Wang *et al.* (2010)	QTL_07
			qDTH7-2	7	106.0	5.43		-0.57	-0.26	2.26	aa07001934	7	114.7	4.28	19,241,445	Unknown	Unknown	QTL_08
			qDTH8-1	8	27.1	38.37		-2.46	-0.55	50.68	aa08000774	8	27.1	38.37	2,229,664	*Hd18*	Shibaya *et al.* (2016)	[24] *Hd18*
			qDTH8-2	8	88.0	8.51		-0.84	-0.60	5.38	aa08004016	8	84.7	8.50	14,786,388	Unknown	Unknown	QTL_09
			qDTH9	9	90.0	3.78 †		0.41	-0.64	0.88	ac09000263	9	105.0	3.19	15,013,511	Unknown	Unknown	Not supported
		MIM	*qDTH1-2*	1	642.0	-	-	-0.38	1.18	6.75	aa01010816	1	643.6	-	37,507,621	*LOC_Os01g62780–OsMADS51*	Yano *et al.* (2016), Kim *et al.* (2007)	QTL_02
			qDTH6	6	58.0	-	-	0.20	-0.65	1.31	aa06001119	6	57.9	-	27,389,348	Unknown	Unknown	Not supported
			qDTH7-1	7	0.0	-	-	-0.60	0.83	7.38	aa07000615	7	0.0	-	2,651,062	*OsMADS15*	Wang *et al.* (2010)	QTL_07
			qDTH7-2	7	104.0	-	-	-0.60	-0.18	3.13	ac07000440	7	94.8	-	16,970,876	Unknown	Unknown	QTL_08
			qDTH8-1	8	24.0	-	-	-2.10	-0.45	60.75	aa08000727	8	25.6	-	1,798,321	*Hd18*	Shibaya *et al.* (2016)	[24] *Hd18*
			qDTH8-2	8	86.0	-	-	-1.21	-0.13	8.45	aa08004016	8	84.7	-	14,786,388	Unknown	Unknown	QTL_09
			qDTH1-2	1	642.0	-	-	1.69 (Both 'K209')	-	2.09	-	-	-	-	-	-	-	-
			qDTH8-2	8	86.0			-1.49 (Both 'KoASBL')										
			qDTH7-1	7	0.0	-	-	3.13 (Both 'K209')	-	4.69	-	-	-	-	-	-	-	-
			qDTH8-1	8	24.0			-2.27 (Both 'KoASBL')										

LOD, logarithm of odds; Chr., chromosome number.

[a] QTL analysis was performed using R/qtl v. 1.47.9 (Broman *et al.* 2003) with composite interval mapping (CIM) and multiple interval mapping (MIM).

[b] Peaks above the threshold at the 5% significance level were considered major QTLs, and †peaks above LOD 3 as minor ones. The two framed loci were expected to interact epistatically with each other.

[c] Genetic effects were calculated with the *fitqtl* function of R/qtl.

[d] Additive effect of 'KoASBL' allele AE for the interaction between two QTLs is shown when both the 'K209' and 'KoASBL' alleles are homozygous.

[e] Dominance effect of 'KoASBL' allele.

[f] Percentage of phenotypic variance explained by the QTL.

[g] Marker closest to the QTL peak.

[h] Position on the 'Nipponbare' IRGSP-1.0 reference genome (Kawahara *et al.* 2013).

Supplemental Table 8. Detailed information about HDA1, a set of 96-plex SNP genotyping assays for the Fluidigm genotyping platform

Target gene or QTL	Assay name	Serial number in this set	Serial number for each locus	Chr.	Target mutation position[a] (bp)	Allele 1[b] (FAM)	Allele 2[b] (HEX)		Direction[c]	Appropriate PCR cycle conditions[d]			Allele 1-specific primer[e]	Allele 2-specific primer[e]	Locus-specific primer	Specific target amplification primer
										Std.	Plus 6	Plus 12				
OsGI	FA5546	1	1	1	4330799	T	C		-	◯	◯	×	GTGGATGCGCTTTGTGACA	GTGGATGCGCTTTGTGACG	AGCTGAAGCCTTGGTCGGT	TCTTTGTTTTTCATGGGTAGGTTGT
OsGI	FA5547	2	2	1	4331676	G	T		+	◯	◯	◯	ATTGTGTAGCTCGTTACTGATACCAG	CTATTGTGTAGCTCGTTACTGATACCAT	AGGACTCCATAAAGATAGTCATACCTGGA	GAATCGCTGAGCTAATGCTATTGT
OsGI	FA5549	3	3	1	4333310	C	T		-	◯	◯	×	TTTCCGAATACTCTCCCAACCG	TTTTCCGAATACTCTCCCAACCA	CCTTCGGCATGTCTCAGTGGA	CTGATACGTCTGCTGCTTTACTT
OsGI	FA5554	4	4	1	4340906	T	C		+	◯	◯	×	GCCCCACCCATTGGCTAATT	CCCCACCCATTGGCTAATC	CCAAATTTCGACCTAATCACAATTCACACA	CCATTATTGTCGTGGTGGGC
QTL_01	FA0057	5	1	1	5590233	A	G	†	-	◯	◯	×	TCCTCTTGAATCCCTTCAATTCCAT	TCCTCTTGAATCCCTTCAATTCCAC	GGTGTGTGGTACCTGGTACCT	TCAATTCCCCTGTCACATCCT
LOC_Os01g62780	FA5414	6	1	1	36355847	G	A		+	◯	◯	×	TGTTGCCGGATATGTGCAATTTG	GTGTTGCCGGATATGTGCAATTTA	GCTCACATACTGAAGAACTGGAACTCT	TTGGTTGAATTGTGTCAGGTGT
LOC_Os01g62780	FA5420	7	2	1	36357690	G	A		-	◯	◯	×	TGTAAATTCTCTGGCCCCGC	CTGTAAATTCTCTGGCCCCGT	AGTCTCAGCGAGGCCATCTG	GGATTATGAACTTGTCTCCCAACT
LOC_Os01g62780	FA5421	8	3	1	36357921	A	C		+	◯	◯	×	AGGCATCCAATGGGACACAA	GGCATCCAATGGGACACAC	GTTCCACTGTTTGGAGCTGTGT	CCAAACAGTAGGGCACCAAAATTA
QTL_02	FA0118	9	1	1	37507621	T	C	†	-	◯	◯	×	GCTCCATCAAATTCTACAGACCACA	CTCCATCAAATTCTACAGACCACG	GCTACGCATTAGTGTTTTGTACGGT	CAACACGAAGAGCTTGCTCC
OsMADS51	FA5571	10	1	1	40343339	A	G		+	◯	◯	×	AGCAATTTAAGGGTAATGCTCTTCTCTA	GCAATTTAAGGGTAATGCTCTTCTCTG	AATGGCATGGCAGGTCAAATGAA	TGATGTGCAAACGGAGCAATT
OsMADS51	FA5572	11	2	1	40348092	A	C		-	◯	◯	×	ATAATTTATCACATATATGGTCAGCATATTATGTGT	ATAATTTATCACATATATGGTCAGCATATTATGTGG	TGTTTTATGAATACTAATATGTATTTTATACGCACGTAAG	TCATTCATAATTTATCACATATATGGTCAGCA
OsMADS51	FA6346	12	3	1	40348975–40359723	10749bp	DEL	‡	-	◯	◯	×	TGCATGTACTTTCATCCGTACGA (No TAG) [P1]<br>TGCATGTACTTTCATCCGTACGA [P4]	TGTTGTGAGACCAACTATGACACA (No TAG) [P2]<br>TGTTGTGAGACCAACTATGACACA [P5]	GGATATTCATTGCAACTTACAGGCA [P3]	(Not set)
OsVIL2	FA5712	13	1	2	2880308	A	G		-	◯	◯	◯	AAATCATCTGGTGGTGGCGT	ATCATCTGGTGGTGGCGC	GACTGATGGTTGGCTGTGTTGG	TATCTCTTCAGAGTTGTGACTGGC
QTL_3	FA2420	14	1	2	11579446	G	T	†	-	◯	◯	◯	CTCCTCCCCTGCTTCGTC	CCTCCTCCCCTGCTTCGTA	CAAGCGCCGCATCGCTA	CGCTTCTTCATCATCATCGTCAT
OsCOL4	FA5568	15	1	2	23996520–23996521	DEL	A		-	◯	◯	×	ATTCTTAGATAAAATTTAGACAAAATTCAAATGCTG	ATTCTTAGATAAAATTTAGACAAAATTCAAATGCTT	GGTGGGTCCCGGACCA	TCTTATATTCTTAGATAAAATTTAGACAAAATTCAAATGC
DTH2	FA5542	16	1	2	30098026	T	G		-	◯	◯	×	TGACGATACCCCACAATCTTGAAAATA	GACGATACCCCACAATCTTGAAAATC	TCCTTCTCTGGTTTGACTGGTGAAA	TCACCCATAAGGATCATTGACGA
DTH2	FA5543	17	2	2	30100200	A	G		-	◯	◯	×	TGGTTCCATGCATGCATAGCT	GGTTCCATGCATGCATAGCC	CGGACCGGGAATCCTGGATG	AGGAAACGCGGGTGGT
Ehd4	FA5478	18	1	3	719949	A	C		-	◯	◯	◯	CCCCGATTAGCATTACCAGCAT	CCCCGATTAGCATTACCAGCAG	AGGCGTTAGGGAACACCAAGA	TTCCGCTGGCTGCCC
Ef-cd	FA6351	19	1	3	1270518–1270519	DEL	T		+	◯	◯	◯	GTGTCATTGCATGCCTAGCA	GGTGTCATTGCATGCCTAGCT	AGCTGGATCTCTGCGTTGAGAA	CATGGACGACAATACGGGTAAG
OsMADS50	FA5462	20	1	3	1270709	G	A		+	◯	◯	×	GGTCCGGGTTCTCATCTTCG	GGTCCGGGTTCTCATCTTCA	TCAGCCTCCCTTGTCTGCTC	TCGTTGGTGGTGTTGATGTTAC
OsMADS50	FA5466	21	2	3	1276141	G	T		-	◯	◯	◯	GGAAAAGTGGCACAAGGTTAAGAAC	GGAAAAGTGGCACAAGGTTAAGAAA	GCAATTGTTCCTATATGTTTGCTTCTAATGAGA	GTTGGAATACCCATAGTGGGAAA
OsMADS50	FA5705	22	3	3	1283280–1283281	DEL	TTC		+	◯	◯	◯	ACAAGTCACGTTCAGTAGTTAATGC	CCAACAAGTCACGTTCAGTAGTTC	AGAATCCATGGACTGGTTGTGACA	CCGTGTAGATATGCGTCACTTAG
QTL_04	FA0192	23	1	3	1763199	A	G	†	+	◯	◯	◯	CGTACCAAGTCCCATGTATGCA	CGTACCAAGTCCCATGTATGCG	GCTTGGGGTCCACAGCC	TCAGAGCATTACTAGTTAATTAATGCCAC
QTL_05	FA0193	24	1	3	2196417	T	C	†	+	◯	◯	◯	GTGTGTGTTGCGGCTGT	GTGTGTGTTGCGGCTGC	CGGTTATTTATACCAACCGGGACCAAA	GTGAAGGTGTGCGACGT
DTH3b	FA5647	25	1	3	10481041	C	T		+	◯	◯	◯	TCGGTTGCAAGACAGTCAGATC	TCGGTTGCAAGACAGTCAGATT	TGCCCTGTGCAGCGAGTA	CTTTTGGAGCAGTAGAACCATCTT
Hd6	FA5410	26	1	3	31510021	A	G		-	◯	◯	×	CCATATTGAGCTTGATCCTCAGCTT	CCATATTGAGCTTGATCCTCAGCTC	CATGGCAACGTACCTCCCAA	CACTAAATGCTTATTTGAACAAGTACCAT
Hd6	FA5411	27	2	3	31511704	T	G		-	◯	◯	×	CCCTTAATCCTGTGTGTTAAGTTTGTTTAAA	CCCTTAATCCTGTGTGTTAAGTTTGTTTAAC	GGGCCATAAACAGAAGGAAAATATTGTAAAGA	AGTAGCATCATTTGCCCTTAATCC
Hd6	FA5408	28	3	3	31512460	A	T		-	◯	◯	×	TGTGGAGGTCCAAACATTGTGT	TGTGGAGGTCCAAACATTGTGA	CTAGGAGTCTTAGAATGTTGATCTCTGACA	CAGAATCTTTGTGGAGGTCCAA
Hd16	FA5385	29	1	3	33001200	C	A		+	◯	◯	◯	GTGAGTGCCTATTTGCTTGGC	GGTGAGTGCCTATTTGCTTGGA	TCCAGCCATTCAGTGCCTCA	GCTTGAGAGGTGAGTGCC
Hd16	FA5723	30	2	3	33001571	G	C		+	◯	◯	◯	GAAGTTGGGTAAAGGTGGATTTGG	AAGTTGGGTAAAGGTGGATTTGC	AAATGAAACCTCTTGCGCATCTGG	TCCCCAGAGTATATAACTGATAGGA
Hd16	FA5391	31	3	3	33002789	G	A		-	◯	◯	◯	TGGGCATGGACGCTAGC	GTGGGCATGGACGCTAGT	AACTTACCTTTTTCTTTTCGACATTTTACAGGG	CTGCTACCTGTACGACCTAAGT
QTL_06	FA1067	32	1	5	75314	G	C	†	-	◯	◯	◯	ACGATGTTGCAAACAAAATACTCGTC	ACGATGTTGCAAACAAAATACTCGTG	CAACTGCACTTAGCCATGCCA	GTAAAGCAAGCTGAAGGAACGAT
OsHDT1	FA5714	33	1	5	29754410	A	T		+	◯	◯	◯	CACATACACATACCTTCTTGGGAGTA	ACATACACATACCTTCTTGGGAGTT	TTCTGAGGATGATTCCAGCGAAGA	TATAAGTGCACAGAAACAAGCAGA
Hd17	FA5392	34	1	6	2235191	A	G		+	◯	×	×	GGCTTATAGACAAGGCCTTCCA	GGCTTATAGACAAGGCCTTCCG	TGGCTTGTCCCTGTCATGTCT	GGGCACGGACCAGAATAAG
Hd17	FA5395	35	2	6	2238719	G	A		-	◯	◯	◯	TGCTAAATTGGTACTAGTAGGCGAC	CTGCTAAATTGGTACTAGTAGGCGAT	GATTTGCAAGGCGTTTAGCTCCA	GTAGTGTGTGCGACTGCTAA
RFT1	FA5490	36	1	6	2928178	G	A		-	◯	◯	×	GCTCTCGTAGCACATCACCTC	GCTCTCGTAGCACATCACCTT	GAATAACGTAGATAATTAAATTGATGCAGGGCA	CCATGGTTGGCCTTGGG
Hd3a	FA6345	37	1	6	2939866–2939867	DEL	4939bp INS	‡	+	◯	◯	◯	CTGACCGAGCTAAGAGAGAGAGAG (No TAG) [P1]<br>CTGACCGAGCTAAGAGAGAGAGAG [P4]	TCGTTTACTTCATATTTCTATACGTCGT (No TAG) [P2]<br>TCGTTTACTTCATATTTCTATACGTCGT [P5]	CATGTCCATGTCCAACTTTCACTTTA [P3]	(Not set)
Hd3a	FA5482	38	2	6	2940504	T	G		-	◯	◯	◯	ACCTTCAGGTACACACCACAAAA	CCTTCAGGTACACACCACAAAC	GACTTTAAAATGCATTAATTTGATGTTTGTCATGGT	GCATCTGGGTCTACCATCACC
Hd3a	FA5485	39	3	6	2942192	C	G		-	◯	◯	×	TCGGCGAAGTCCTTGGTG	TCGGCGAAGTCCTTGGTC	TCAGACAGTGTACGCGCCC	CGAGCCGAGGTTGTAGAGC
Hd3a	FA5487	40	4	6	2942201	C	T		-	◯	◯	×	GGTTGTAGAGCTCGGCGAAG	GGTTGTAGAGCTCGGCGAAA	CCCGGGTGGCGTCAGA	GACCGGCGAGCCGAG
Hd1	FA5499	41	1	6	9333525	A	G		+	◯	◯	◯	CCGAGCCGGTGGCA	CCGAGCCGGTGGCG	GGATCGAGGTCCACTTCATCCA	AGCTTGACTCTGTACATGAGGG
Hd1	FA6347	42	2	6	9336368–9336369	DEL	4424bp INS	‡	+	◯	◯	◯	TCCCTAGCTCCTTCCAAAAAACAC (No TAG) [P1]<br>TCCCTAGCTCCTTCCAAAAAACAC [P4]	CTGCAAGTAGAAAGCGTCGGAG (No TAG) [P2]<br>CTGCAAGTAGAAAGCGTCGGAG [P5]	GTATGCGTCTTCTCCTGTTCCTC [P3]	(Not set)
Hd1	FA5656	43	3	6	9336867–9336868	DEL	36bp INS		-	×	◯	×	GGCCGGGAGCGGGTT	GGGACGCGCTGGTGG	CGTGGCGTGCGACGTG	CTCAGCGAGGACGGAGG
Hd1	FA5748	44	4	6	9337102	G	DEL		+	◯	◯	×	GATGAGTACTTTGATCTTGTCGGG	CGATGAGTACTTTGATCTTGTCGGTA	ATCTTGGTTGTTTTCGATGCGGTT	CAACGGCATGTATTTTGGTGAAG
Hd1	FA5515	45	5	6	9337242–9337284	43bp	DEL		+	◯	◯	◯	GGGAGCGAGTGTGTGGTA	GGGAGCGAGTGTGTGGTT	GGCGGCCTGGTCTGCT	CAAAAGGAGTTTGCAGAGAAGGA
Hd1	FA5508	46	6	6	9338005–9338006	TT	DEL		-	◯	◯	◯	CCCGCCTCCATTGATGAGAA	CCCGCCTCCATTGATGAGAG	CAGTGACCATTTGCCGATTCCA	CACCGTGCTGTCTGGTACTATA
Hd1	FA5746	47	7	6	9338031–9338032	DEL	1901bp INS		+	×	◯	◯	GCGGGTATAGTACCAGACAGC	TGTGTGTGTCTCCGATCTTGG	CCTGTCCATGGAGCTGAAGTGA	TCTTTAGGCAGACCAGTTGACT
Hd1	FA5509	48	8	6	9338068	A	C		+	◯	◯	×	CGGTGATAGATATGCCAAATTCCAGA	GGTGATAGATATGCCAAATTCCAGC	TGATTGCTCCAGCAGGTGTCA	ACCAGACAGCACGGTGA
Hd1	FA6350	49	9	6	9338183–9338185	AAG	DEL		+	×	◯	◯	CTCAGGTACAGGGAGAAGAAGAAG	CAGGTACAGGGAGAAGAAGGC	CCCTTGATCCGGGGTCGTG	TCCATGGACAGGGAGGC
Hd1	FA5747	50	10	6	9338224–9338227	AAAG	DEL		-	◯	◯	◯	TGCCTCTGCATACGCCTTT	CCTCTGCATACGCCTTGTTTC	AGGCCAGGAAGTTTGAGAAGACAA	TTCGATCTGCACATCTGATCTCTT
Hd1	FA5749	51	11	6	9338243	C	T		-	◯	×	×	CCCTTGATCCGGGGTCG	GCCCTTGATCCGGGGTCA	AGGCCAGGAAGTTTGAGAAGACAA	TTCGATCTGCACATCTGATCTCTT
Hd1	FA5511	52	12	6	9339368	T	C		+	◯	◯	×	CTCAGTGCATTCCTCTTCCCTT	TCAGTGCATTCCTCTTCCCTC	CAAAAGGCAATAAGATCCATGCCACT	AGGAAGCTCTTTCTCATTCTCAGT
Hd1	FA5512	53	13	6	9341481	C	T		-	◯	◯	◯	CGTAAGGGTGAAAATGGTCACG	TCGTAAGGGTGAAAATGGTCACA	TGGCTCTCGATCCACGGG	GACGTAGCCGCATTCCTC
Se5	FA5638	54	1	6	23855144	A	T		-	◯	◯	×	TGAGTCATAATTACTTGAACGTCCACAT	TGAGTCATAATTACTTGAACGTCCACAA	AGTAGTTTCAATTTTCCATCTAAAAAGAATATTACAGATT	GTATGGAAGTATGGAACAATGAGTCA
Se5	FA5639	55	2	6	23855920	G	T		-	◯	◯	◯	TCTTTCTGGAATCCGCCTTGTC	ATCTTTCTGGAATCCGCCTTGTA	AAGGGTGTAACAAAGACTCAAGGGTTA	AGGAAATCCTTGAGTCTTTATAACAGAATC
Se5	FA5644	56	3	6	23861465	T	A		+	◯	◯	×	ACTGACATTTTGTTGTCTCTCGCT	CTGACATTTTGTTGTCTCTCGCA	AAAGTTTTACTTGGATCAGCCAATACAGC	GGTCGATTTGTGTTATCCCTATATTTACT

(Continued)

Target gene or QTL	Assay name	Serial number in this set	Serial number for each locus	Chr.	Target mutation position[a] (bp)	Allele 1[b] (FAM)	Allele 2[b] (HEX)		Direction[c]	Appropriate PCR cycle conditions[d]			Allele 1-specific primer[e]	Allele 2-specific primer[e]	Locus-specific primer	Specific target amplification primer
										Std.	Plus 6	Plus 12				
OsFTIP1	FA5724	57	1	6	24557073	A	C		-	○	○	○	CGTTCGAGGAGCAGCTGT	CGTTCGAGGAGCAGCTGG	TCCTTGCGCGGCGTCA	GGAACGAGGACCTGGTGTT
QTL_07	FA0419	58	1	7	3654626	T	C	†	-	○	○	×	AATAAACAAACAATTGCTGGAGTGAGAA	ATAAACAAACAATTGCTGGAGTGAGAG	CAGTGGTTGGGTTAACAACAAACTGA	GGCACTCTCTGAAGTTCCAAATAAAC
Ghd7	FA5519	59	1	7	9150835	A	G		-	○	○	○	CAGCAGTTTGCAGTTGGACT	CAGCAGTTTGCAGTTGGACC	AGTATCGATTATAAGAATTTCTCTATAATTTCATGGTAGA	AGTATTGGCTACTCAGTTGATCCA
Ghd7	FA5525	60	2	7	9152479	G	C		-	○	○	○	GGTCGCTTCGCCAAAGAAC	GGTCGCTTCGCCAAAGAAG	GGCGGTGCGACAGCTT	GAGGCCCCGAGTGAGA
Ghd7	FA5526	61	3	7	9152655	T	A		+	○	○	×	GGGTTCAAGCTCTCCCCAT	GGGTTCAAGCTCTCCCCAA	TCGATGCCCAAGGAGATGGT	CGCCAACCACCGTGTTT
Ghd7	FA5530	62	4	7	9154664	C	A		+	×	○	×	GTTGCCGAAGAACTGGAACTC	CGTTGCCGAAGAACTGGAACTA	GGATCGGCGCCCCGG	CGCCGTCGTTGCCGA
Ghd7	FA6352	63	5	7	9155047–9155048	DEL	1901bp INS		+	×	○	○	GTTGGGGATGAGGGAAAGGTAG	CACTCTCGTGTTTGTGTGTGTC	GTTGCAATGGGGATGGCCAATG	GACAGGGCAAGTTGGGGATGAG
Ghd7	FA5533	64	6	7	9156042	C	G		+	○	○	○	CTTTCGATATACCGTGGTTGCTC	CTTTCGATATACCGTGGTTGCTG	CGCGCCACACCCTCAATCTA	CATTGCCATAAGGTTACCTTTCGA
Ghd7	FA5734	65	7	7	9156348	G	A		+	×	○	×	GTCCCTGACGGATGGTGG	AGTCCCTGACGGATGGTGA	GTTTCGGCAAGGGGATGTCTAAAC	AAACGTGTTTGCCAGGCGAAA
QTL_08	FA0444	66	1	7	16622242	A	G		-	○	○	×	CGGTATCGTGTTTAGTAGTACTTAAAACACT	GGTATCGTGTTTAGTAGTACTTAAAACACC	CAATACTATCTTATTTTTACTCTCACACTCCGGAT	CGGCGCGGTATCGTGT
OsMADS18	FA6353	67	1	7	24791829	T	C		+	○	○	×	GGTGAACAATTGGACACACTAACAAT	GTGAACAATTGGACACACTAACAAC	ATTCCAGTTGATGTTCCAATTGCTG	TGGTCTATCTACTGCTCAAGGC
OsMADS18	FA6354	68	2	7	24792084	A	C		-	○	○	×	AAACAATATGGTATAAACAGACTGCTACATT	ACAATATGGTATAAACAGACTGCTACATG	GGTTACCCTCAATGTGGCTCCT	GGCAAGAAGAGTTAGTTGTCTGA
PRR37	FA5437	69	1	7	29617569	G	C		+	×	○	×	TCTCCGGTGTGCAGCG	TCTCCGGTGTGCAGCC	TTCTGGATGAAGCGCTCCCA	CCGGATGGCGGACCAA
PRR37	FA5778	70	2	7	29617674	G	DEL		+	○	○	×	GTCAGTGCCCTGCTTCGT	GTCAGTGCCCTGCTTCACA	ACAGAAGTGAGAAGGTAGAAGGAGG	AGCGATGACTCCACCAGG
PRR37	FA5433	71	3	7	29623803	G	A		+	○	○	○	GCAATGACGATGATGACGACG	AGCAATGACGATGATGACGACA	TGCCATCTCTTGCATTAAGTCCCA	GGATGAATCCAATAATAACAATGGCAG
PRR37	FA5441	72	4	7	29626909	G	A		+	○	○	○	ACAGGCAGCAAGAGCCG	ACAGGCAGCAAGAGCCA	TCCCTTCTGGCACTTTGGAGG	GGCCAAAAACATGGATGCAC
PRR37	FA5779	73	5	7	29627358–29627365	GAACGTTG	DEL		-	○	○	○	ATTTGTACTAGGCTTTGTCACAACG	CTCCTTATTTGTACTAGGCTTTGTCATTT	TGCTGCACCAATAAAACAAGGTTCT	AGGGTGACATTACTCTCTCCTTAT
PRR37	FA5780	74	6	7	29628481	T	C		+	○	○	○	GCCATTTTCAGGTGCGGT	GCCATTTTCAGGTGCGGC	TGCTGTTGTTGGTCTTGCACAG	GGTTTCTTTCTGATGGCTGTCT
PRR37	FA5781	75	7	7	29628484	C	T		-	○	○	×	GCCAGCCTCTTTCTGCTCTG	GCCAGCCTCTTTCTGCTCTA	CTGTCTGCCATTTTCAGGTGCG	GTTGTTGGTCTTGCACAGCTT
PRR37	FA5436	76	8	7	29628500	T	C		-	○	○	×	CCGCTGCTCGGCCA	CCGCTGCTCGGCCG	CTGCCATTTTCAGGTGCGGTA	TGTCCGCGGACCCTTG
Ehd3	FA5562	77	1	8	274129	T	G		+	○	○	×	GGGAAGCTTGGGATCTTCTTCT	GGGAAGCTTGGGATCTTCTTCG	AGCTCGAGCTGGTGAAGACA	CCTCTCTTTCTTCAGCTAATTCAATTTTTATG
Hd18	FA5401	78	1	8	2381725	C	T		+	○	○	○	GGAATGTACCATTGGTTTCCATCTC	AGGAATGTACCATTGGTTTCCATCTT	ACATCCACACGAATGTGTCCTCT	ACCAATATGCAATGAGATCAGGAATG
Hd18	FA5404	79	2	8	2387949	T	C		+	○	○	×	CCCCAACCTCTGGATTGCT	CCCCAACCTCTGGATTGCC	TTGAACCGGAGTTGCCAGAGA	GCGACTTTGTTCAACAACCCA
Hd18	FA5400	80	3	8	2388554	T	C		-	○	○	×	TCATCACCGGCATCCACA	TCATCACCGGCATCCACG	CCGAGCTGCCTGGCGA	TTGAGCTCGGCGGCA
OsLHY	FA5725	81	1	8	3372323	T	C		+	○	○	○	GCAAGAAACTCGGAAACAAGATAATTT	GCAAGAAACTCGGAAACAAGATAATTC	GCGACACTTCTCCTTTTCCACTCT	CCAATGGATAATGCACAGAAGGAA
DTH8	FA5449	82	1	8	4332710	G	A		+	×	○	○	TCGTCCCAACGCTGCTG	TTTCGTCCCAACGCTGCTA	AGCTGCCAACGATGCTGTG	TGTCTCACTAACGATGGTGACT
DTH8	FA5450	83	2	8	4333856	T	C		+	○	○	×	CGTGTGCCTAGCTAGCTAATTAGTAT	CGTGTGCCTAGCTAGCTAATTAGTAC	CTCCATGGCGTGCAATGGT	AGTAATAATAAAACTACGCGTGTGCC
DTH8	FA6349	84	3	8	4333870–4333871	AA	TC		+	×	○	○	CTAGCTAGCTAATTAGTATTCCTCTTTAGTCTA	GCTAGCTAATTAGTACTCCTCTTTAGTCTT	TTCGCCGGCCACCTCC	AGCATCAACAATTAATTAACGCGG
DTH8	FA5777	85	4	8	4334417	T	DEL		+	×	○	○	TTCTCGCGCTGGCACTT	TCTCGCGCTGGCACTG	TGTCGGAGTTCATCAGCTTCGT	GTCGCCGTTGATGGTCTTC
DTH8	FA6355	86	5	8	4335990	A	C		-	○	○	○	CCTTTGTCCTGCCTTTTCCTTTT	CCTTTGTCCTGCCTTTTCCTTTG	TGCTCTCACTTCACTTGTGATGGA	GTGGAAAAGGGGATAAGAGAAGC
DTH8	FA5453	87	6	8	4359634–4359635	DEL	AT		-	○	○	○	GTGAGGTACGTCCTGCAAATG	GGTGAGGTACGTCCTGCAAATA	CTTGAGCTGTGGCTCTGTGC	TTTTACTTATTAAAAGCATCAGGTGAGGT
QTL_09	FA2167	88	1	8	12331561	T	C	†	+	○	×	×	TCCAATTTTCTAGAAATGCAATTATTATGGGAT	TCCAATTTTCTAGAAATGCAATTATTATGGGAC	ACTTGTAATATAATTCCTTGTACTAACAATTACTACCTCC	GTTTGTTGTTAATCTGTCTCCAATTTTCTA
QTL_09	FA0494	89	1	8	14786388	T	C		+	○	○	○	TCACCTGAATAGAATCATGCTTGTCT	CACCTGAATAGAATCATGCTTGTCC	TTGCTTGCCGTCATCAGCC	GTATTGGTGTTGCACTCACCT
Ehd2	FA5569	90	1	10	14739998	C	T		-	○	○	×	GCAACTTCGCCGCCG	TGCAACTTCGCCGCCA	GTCGGTGGCGCAGCA	CCGCCTGCCAGGGTT
Ehd1	FA5455	91	1	10	17077589	C	T		-	○	○	×	GCAGCAGTGAACCACCTCG	GCAGCAGTGAACCACCTCA	ACTGCAAAGAAGTTTAATTTGATCACTCACTG	CAGTTGCACCGTCAGTTCAT
Ehd1	FA5458	92	2	10	17078199	A	G		-	○	○	×	CGTGACGAGACATTGGCCT	CGTGACGAGACATTGGCCC	GAGCAGCCCATTGTGACGG	AGTTCAACCCTATCAGAAATAATATCTTTCG
QTL_10	FA0603	93	1	10	22389675	T	G	†	+	○	○	×	CTGAGGAGATATTTCTCTTCAGACTCTTT	CTGAGGAGATATTTCTCTTCAGACTCTTG	GGCTACTGTCATCATTATTTCTGAAATTCCTG	TGAGTCATGTCCTTCTGAGGAG
LOC_Os11g08410	FA5425	94	1	11	4432316	A	G		-	○	○	○	GGTTCGTTCTGTTTCTTTTAACTCTTCT	GGTTCGTTCTGTTTCTTTTAACTCTTCC	GGCTCAGAGGTAAGGTAACACCA	GGAATATGAGTGAATTCGGTTCGT
QTL_11	FA1674	95	1	12	4000543	G	C	†	+	○	○	×	CGCCGCCTCTGCCG	CGCCGCCTCTGCCC	GCAGGGAGGCGCGTCA	CCCGGAGGAGTTCTTCGTC
QTL_12	FA1759	96	1	12	24387429	T	G	†	+	○	○	×	AATTGCTGCATCAGTGATTTTCTGT	ATTGCTGCATCAGTGATTTTCTGG	AGAGTACAGAATGTTATGTGCGCAGT	ACTGAGAAATTTCTCAACGAATTGCT

Chr., chromosome number; Std., standard; INS, insertion; DEL, deletion.

[a] Position on the 'Nipponbare' IRGSP-1.0 reference genome.

[b] The 'Nipponbare' allele corresponds to "Allele 1" and to "Allele 2" when labeled with †. Only nucleotide bases of < 11 bp are shown.

[‡] Special design suitable for a large insertion or deletion as in Supplemental Fig. 3; the P1–P5 labels in the primer sequences are used in this figure.

[c] Allele-specific primers were designed on the plus ("+") or minus ("−") strand of the 'Nipponbare' IRGSP-1.0 reference genome.

[d] The scatter plot is (○) or is not (×) appropriate for determining the allele type.

[e] Tag sequences for the fluorescent label are not shown.

Supplemental Table 9. Summary of multiple linear regression analyses for constructing prediction models

Gene and QTL number[a]	Locus name	Allele No.[b]	Model_April				Model_May				Model_June			
			Regression coefficient	Standard error	*P*-value[c]	VIF	Regression coefficient	Standard error	*P*-value[c]	VIF	Regression coefficient	Standard error	*P*-value[c]	VIF
	(Intercept)		113.4843	1.1511	***		99.8745	1.1597	***		81.7631	1.0736	***	
[1]	*OsGI*	A02	-4.6483	1.9924	*	1.39								
		A04	-4.2526	2.073	*	3.54								
[2]	*LOC_Os01g62780*	A02	5.1406	1.2414	***	2.68	4.6585	1.1728	***	2.57	4.5948	0.9899	***	2.83
QTL_02	*FA0118*	A02	-2.4149	0.9493	*	2.02	-2.4981	0.7989	**	1.54	-2.2996	0.6718	***	1.68
[3]	*OsMADS51*	A02	7.9345	2.7978	**	2.75								
[6]	*DTH2*	A02	3.1864	1.5081	*	2.65					2.6883	1.0708	*	2.22
[7]	*Ehd4*	A02	13.3356	3.0375	***	5.49	10.7888	2.2143	***	3.14	8.8396	2.1112	***	4.41
[11]	*Hd6*	A02	3.4404	1.0723	**	2.48	4.7940	1.1042	***	2.82	5.9021	0.9226	***	3.05
		A04					3.4211	1.5826	*	1.50	5.2811	1.2860	***	1.53
[12]	*Hd16*	A02									9.4666	1.5951	***	3.17
		A03	-6.2275	1.1697	***	1.46	-6.1121	1.1336	***	1.47	-3.3948	0.9202	***	1.50
QTL_06	*FA1067*	A02	2.5645	1.0095	*	1.43	2.0700	0.9662	*	1.41	1.8586	0.7849	*	1.44
[13]	*OsHDT1*	A02	-3.7943	1.1158	***	2.81	-3.0261	0.9325	**	2.10	-2.7235	0.8560	**	2.74
[14]	*Hd17*	A02	3.0052	0.9881	**	2.23	3.1860	0.9042	***	2.01	3.4887	0.7533	***	2.15
[16]	*Hd3a*	A05	-11.7437	4.1877	**	1.59								
[17]	*Hd1*	A02					2.7948	1.0636	**	2.19	2.3700	0.8677	**	2.25
		A03	-30.7753	3.9289	***	3.44	-23.5766	3.1642	***	2.40	-17.0354	2.6633	***	2.63
		A04	-12.9099	1.0384	***	1.85	-9.3718	1.0170	***	1.91	-4.0750	0.8365	***	1.99
		A05	-8.9102	2.9483	**	1.17								
		A08	-11.4945	3.9266	**	1.40								
		A09	-15.8677	1.883	***	1.69	-12.6043	1.7005	***	1.48	-7.1530	1.5025	***	1.78
		A11	-16.0911	1.8799	***	2.10	-14.2098	1.7603	***	1.98	-9.3725	1.4663	***	2.13
		A12	-15.7177	1.8191	***	1.44	-14.4271	1.8300	***	1.56	-11.5948	1.4926	***	1.61
		A13	-33.9317	4.9432	***	1.11	-31.0935	4.7484	***	1.10	-19.6898	4.0962	***	1.27
[18]	*Se5*	A02									-2.4158	1.2496	.	1.65
		A04	25.7959	3.3872	***	2.06	23.6960	3.1785	***	1.95	17.8597	2.5932	***	2.00
QTL_07	*FA0419*	A02	-1.8079	1.0394	.	2.41								
[20]	*Ghd7*	A06	-29.9798	1.8217	***	1.71	-25.9098	1.8845	***	1.97	-16.9589	1.5633	***	2.09
		A07	-15.2161	2.3200	***	1.43	-16.9922	2.2617	***	1.46	-15.9093	1.8428	***	1.50
QTL_08	*FA0444*	A02	-2.0729	0.8433	*	1.40	-1.8939	0.7910	*	1.33	-1.5357	0.6535	*	1.40
[22]	*PRR37*	A02	-2.1164	1.0488	*	1.94	-2.1093	0.9842	*	1.83	-1.8312	0.7975	*	1.86
		A03	-5.5023	1.1339	***	1.58	-6.0575	1.0976	***	1.59	-5.2978	0.8893	***	1.61
[24]	*Hd18*	A02	2.2804	0.7685	**	1.27	1.6897	0.7360	*	1.25	2.5818	0.6167	***	1.35
QTL_10	*FA0603*	A02									1.1190	0.6577	.	1.63
QTL_11	*FA1674*	A02	-2.3745	0.8267	**	1.54	-1.7864	0.8079	*	1.58	-1.7992	0.6559	**	1.61
	Multiple R-squared			0.9062				0.8943				0.8961		
	Adjusted R-squared			0.8895				0.8799				0.8791		
	Residual standard error			4.6762				4.5120				3.6287		
	F-statistic[c]			54.3996*** (DF = 30; 169)				61.7219*** (DF = 24; 175)				52.6951*** (DF = 28; 171)		
	RMSE			4.2985				4.2206				3.3553		

VIF, variance inflation factor.

[a] See Tables 1 and 2.

[b] See Supplemental Fig. 4.

[c] ***p < 0.001; **p < 0.01; *p < 0.05; ·p < 0.1.
